# Engineering an efficient and tight d-amino acid-inducible gene expression system in *Rhodosporidium*/*Rhodotorula* species

**DOI:** 10.1186/s12934-015-0357-7

**Published:** 2015-10-26

**Authors:** Yanbin Liu, Chong Mei John Koh, Si Te Ngoh, Lianghui Ji

**Affiliations:** Biomaterials and Biocatalysts Group, Temasek Life Sciences Laboratory, 1 Research Link, National University of Singapore, Singapore, 117604 Singapore

**Keywords:** d-amino acid oxidase, Oleaginous yeast, *Rhodosporidium* and *Rhodotorula*, Inducible gene expression system, Promoter and intron

## Abstract

**Background:**

*Rhodosporidium* and *Rhodotorula* are two genera of oleaginous red yeast with great potential for industrial biotechnology. To date, there is no effective method for inducible expression of proteins and RNAs in these hosts.

**Results:**

We have developed a luciferase gene reporter assay based on a new codon-optimized *LUC2* reporter gene (Rt*LUC2*), which is flanked with *CAR2* homology arms and can be integrated into the *CAR2* locus in the nuclear genome at >90 % efficiency. We characterized the upstream DNA sequence of a d-amino acid oxidase gene (*DAO1*) from *R. toruloides* ATCC 10657 by nested deletions. By comparing the upstream DNA sequences of several putative *DAO1* homologs of *Basidiomycetous* fungi, we identified a conserved DNA motif with a consensus sequence of AGGXXGXAGX_11_GAXGAXGG within a 0.2 kb region from the mRNA translation initiation site. Deletion of this motif led to strong mRNA transcription under non-inducing conditions. Interestingly, *DAO1* promoter activity was enhanced about fivefold when the 108 bp intron 1 was included in the reporter construct. We identified a conserved CT-rich motif in the intron with a consensus sequence of TYTCCCYCTCCYCCCCACWYCCGA, deletion or point mutations of which drastically reduced promoter strength under both inducing and non-inducing conditions. Additionally, we created a selection marker-free *DAO1*-null mutant (∆dao1e) which displayed greatly improved inducible gene expression, particularly when both glucose and nitrogen were present in high levels. To avoid adding unwanted peptide to proteins to be expressed, we converted the original translation initiation codon to ATC and re-created a translation initiation codon at the start of exon 2. This promoter, named P_*DAO1*-*in1m1*_, showed very similar luciferase activity to the wild-type promoter upon induction with d-alanine. The inducible system was tunable by adjusting the levels of inducers, carbon source and nitrogen source.

**Conclusion:**

The intron 1-containing *DAO1* promoters coupled with a *DAO1* null mutant makes an efficient and tight d-amino acid-inducible gene expression system in *Rhodosporidium* and *Rhodotorula* genera. The system will be a valuable tool for metabolic engineering and enzyme expression in these yeast hosts.

**Electronic supplementary material:**

The online version of this article (doi:10.1186/s12934-015-0357-7) contains supplementary material, which is available to authorized users.

## Background

**Fig. 1:**
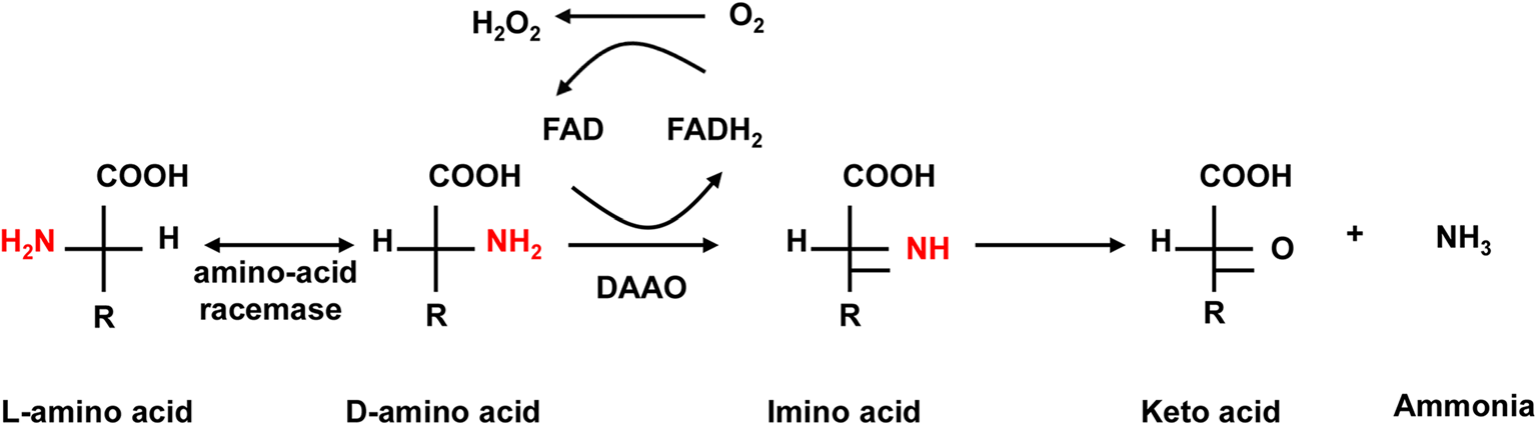
Reactions catalyzed by d-amino acid oxidase. Imino acid is believed be hydrolyzed non-enzymatically to the corresponding keto acid and ammonia. l-amino acids may be converted to d-amino acids by l-amino acid racemase

*Rhodosporidium* (teleomorph) or *Rhodotorula* (anamorph) are phylogenetically highly related yeast and are excellent producers of oil (triacyglyceride) and carotenoid [1, 2]. Dry biomass yield of more than 100 g/L can be readily produced within a week with more than 60 % oil content [3–5]. To take advantage of its high metabolic flux and cell mass production, we have been developing it as a synthetic biology platform. Genetic tools reported include *Agrobacterium tumefaceins*-mediated transformation, constitutive promoter set for gene expression [6–9] and high efficiency gene knockout [7–9]. However, there is no effective means for inducible gene expression to date.

d-Amino acid oxidase [d-amino acid:oxygen oxidoreductase (deaminating)], DAAO (EC 1.4.3.3) is a FAD-dependent oxidoreductase that catalyzes stereospecifically the oxidative deamination of d-amino acids to α-keto acids, ammonia and hydrogen peroxide (Fig. 1). DAAOs have been widely identified, ranging from bacteria, fungi to humans [10]. It is best known for the use in cephalosporin synthesis [11]. DAAO has been used as a marker of peroxisome in many eukaryotic organisms [12, 13]. *R. gracilisDAO1* mRNA transcription has been reported to be inducible by d-alanine (70 mM) [14], with the Dao1 protein accumulated to about 0.3 % of total soluble intracellular proteins after induction [15]. To date, the *DAO1* gene organization and genetic basis of transcriptional regulation remain unknown.

We report here the cloning and characterization of *R. toruloidesDAO1* and the creation of an efficient d-alanine inducible gene expression system for this industrially important yeast.

## Results

### Organization of a d-amino acid oxidase gene *DAO1*

**Fig. 2:**
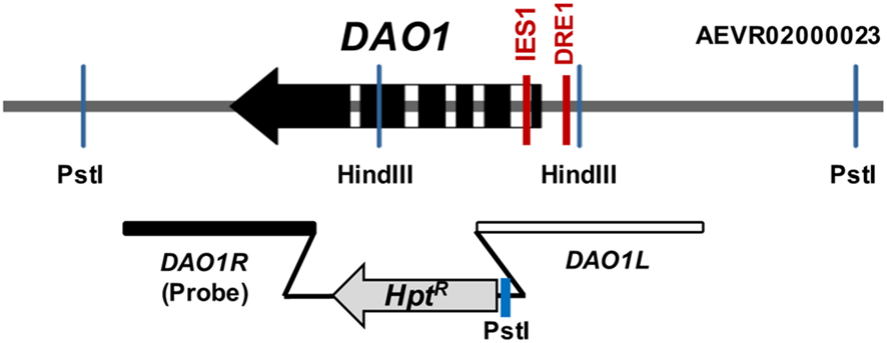
Organization of *DAO1* gene. **a** Schematic diagram of Rt*DAO1* gene. Probe 2 (*DAO1R*) were used for verification of *DAO1* gene deletion in Southern blot analysis. DRE1 and IES1 indicate the position of the d-amino acid responsive element 1 and intronic enhancing element, respectively. *Hpt* ^*R*^: hygromycin resistance cassette. *DAO1R* and *DAO1L*: homology arms used for *DAO1* knockout

Previous studies showed that *R. toruloides* ATCC 10657 and *R. glutinis* ATCC 204091 genes share high sequence homology [6, 16]. Till now, two *RhodotorulaDAO1* sequences have been deposited with GenBank (accession numbers: DM380716 and Z71657) [17]. BLASTn search of *R. glutinis* ATCC 204091 genome identified a homologous gene (EGU13479.1) in scaffold #23. 5′ and 3′ RACE using total RNA of *R. toruloides* ATCC 10657 as template yielded a cDNA fragment of approximately 0.5 kb each (data not shown). The full-length cDNA was amplified by RT-PCR using oligonucleotide pair Rt332f/Rt333r (Table 1) (data not shown). The full-length cDNA (1183 nt) was predicted to encode an ORF of 368 aa with 29 nt 5′ UTR (untranslated region) and 47 nt 3′ UTR. As expected, the ORF is GC-rich with a GC content of 63.0 %. The sequence context (ACGCCATGC) of the putative translation initiation codon fits quiet well with the Kozak consensus of eukaryotes (CC(A/G)CCATGG) [18]. Comparison between the cDNA and genomic sequences revealed 6 exons separated by 5 introns (Fig. 2; Additional files 1, 2). The *DAO1* ORF utilizes 58 codons (Additional file 4). Similar to *GPD1* [6], codon utilization showed strong preference for cytosine at the Wobble position with the exceptions of alanine, arginine, serine and threonine, in which guanine was preferred. The *DAO1* mRNA contains no canonical polyadenylation signal (AATAAA) in the 3′ UTR. Similar to *GPD1*, a short region with TG repeats can be found 28 nt upstream of the polyadenylation site (Additional file 1). *DAO1* homologs of *R. toruloides* strain ATCC 10657 and ATCC 204091 differed by only two nucleotides in the coding region, both being silent mutations (encoding residue I_186_ and A_296_, respectively) (Additional file 1). The Dao1 enzyme was predicted to contain a highly conserved sequence motif (GXGXXG, where “X” indicates any amino acid) as required for FAD coenzyme binding [19]; amino acid residues that are critical for catalytic reaction (Y_223_, Y_238_ and R_285_) [20]; and a C-terminal SKL-tripeptide as the peroxisomal targeting signal (PTS1) [21]; Additional file 1).

**Table 1:** Oligonucleotides used

Name	Sequence (restriction enzyme site)^a^	PCR target
DAO1U1	5′-CACTTTGCTTGTCGAGGACCGTC-3′	5′RACE
DAO1L1	5′-ACGACCAGGTGGCGAAGTGATCT-3′	3′RACE
Rt332f	5′-GCTTGTACTGCTCGAACGAC-3′	cDNA
Rt333r	5′-CTGGTGAAATGCCCCAATAC-3′	cDNA
Rt290Sf	5′-TTT*actagt*CTTCCCGGTCTCGTATCGAG-3′ (*Spe*I)	P_*DAO1*-*in1*_ 2.2 kb
Rt315S	5′-TTT*actagt*ACTCCGCAATCTGCAGAGAC-3′ (*Spe*I)	P_*DAO1m2*-*in1*_ 1.7 kb
Rt314S	5′-TTT*actagt*CATGGTCTGATCGCTTGTGTG-3′ (*Spe*I)	P_*DAO1m3*-*in1*_ 1.2 kb
Rt120S	5′-TTT*actagt*GTGGCAGGTGTGCGTG-3′ (*Spe*I)	P_*DAO1m4*-*in1*_ 1.0 kb
Rt313S	5′-TTT*actagt*CGTTCGTGGGCTCAAGGAAG-3′ (*Spe*I)	P_*DAO1m5*-*in1*_ 0.7 kb
Rt117S	5′-TTT*actagt*CGACGACGGGAAGCTTCG-3′ (*Spe*I)	P_*DAO1m6*-*in1*_ 0.4 kb
Rt287Nr	5′-TTT*ccatgg*CAATCACTGTATAATCAAGAGCTG-3′ (*Nco*I)	P_*DAO1*-*in1*_ reverse
Rt309Nr	5′-TTT*ccatgg*CGTCGTTCGAGCAG-3′ (*Spe*I)	P_*DAO1*_ reverse
Rt311	5′-GAAGCTTCGGCACGAGCATG-3′	P_*DAO1m5*-*in1m1*_
Rt312	5′-ACAGTCATGCTCGTGCCGAAGCTTCGCAACCGCTCATCAGTACAC-3′	P_*DAO1m5*-*in1m1*_
SFGFPSEQ	5′-GGACAAACCACAACTAGAATGCAG	P_*DAO1m5*-*in1m1*_
35STer	5′-AAAGCATGCTAATTCGGGGGATCTGGAT	P_*DAO1m5*-*in1m1*_
Rt288f	5′-GTAGGTTACGCCGATCGAGTTG-3′	*DAO1* Probe
Rt289r	5′-GCTCGACCAACTGCTCTCTTTC-3′	*DAO1* Probe
Rt327r	5′-GGCGTCGTTCGAGCAGTAC-3′	P_*DAO1m5*-*in1m2*_
Rt328f	5′-CTGCTTGTACTGCTCGAACGACGCCATCCATTCACAGAAGCGCGTCGT-3′	P_*DAO1m5*-*in1m2*_
Rt329r	5′-GACGCACCGCCTGATCCGAG-3′	P_*DAO1m5*-*in1m2*_ P_*DAO1m5*-*in1m3*_
Rt330f	5′-TTGTCCTCGGATCAGGCGGTGCGTCTTTAAATATAATAAAAAAAAAAGACAGTTCTCGAGGAGGAGTAC-3′	P_*DAO1m5*-*in1m2*_
Rt331f	5′-TTGTCCTCGGATCAGGCGGTGCGTC(24 mer deletion)CAGTTCTCGAGGAGGAGTAC-3′	P_*DAO1m5*-*in1m3*_
Rt334f	5′-TTGTCCTCGGATCAGGCGGTGCGTCTTTCAATCTCCTCCCCACACCCGACAGTTCTCGAGGAGGAGTAC-3′	P_*DAO1m5*-*in1m4*_
Rt335f	5′-TTGTCCTCGGATCAGGCGGTGCGTCTTTCCCTCTAATCCCCACACCCGACAGTTCTCGAGGAGGAGTAC-3′	P_*DAO1m5*-*in1m5*_
Rt336f	5′-TTGTCCTCGGATCAGGCGGTGCGTCTTTCCCTCTCCTCAACACACCCGACAGTTCTCGAGGAGGAGTAC-3′	P_*DAO1m5*-*in1m6*_
Rt337f	5′-TTGTCCTCGGATCAGGCGGTGCGTCTTTCCCTCTCCTCCCCGCACCCGACAGTTCTCGAGGAGGAGTAC-3′	P_*DAO1m5*-*in1m7*_
Rt338f	5′-TTGTCCTCGGATCAGGCGGTGCGTCTTTCCCTCTCCTCCCCACACAAGACAGTTCTCGAGGAGGAGTAC-3′	P_*DAO1m5*-*in1m8*_
LUC2U	5′-GAAGTACTCGGCGTAGGTG-3′	In Rt*LUC2*, for amplification of P_*DAO1m1*-*in1m1~*_P_*DAO1m5*-*in1m8*_
DAO1f	5′-CTTCGTGCTAACCAAGCTCGT-3′	Probe and colony PCR of *DAO1*
DAO1r	5′-GTCTCAGGGTTGACGGACAAG-3′	Probe and colony PCR of *DAO1*
qDAO1f	5′-TCAAACCGTCCTCGTCAAGTC-3′	qPCR of *DAO1*
qDAO1r	5′-GTTGACGGACAAGTCCCAATC-3′	qPCR of *DAO1*
qACT1f	5′-TACCCAACTTGTCCCAACCTG-3′	qPCR of *ACT1*, reference gene
qACT1r	5′-CTCGTCTCCATCACCATCCTC-3′	qPCR of *ACT1*, reference gene
DAO1L-Sf	5′-AAA*gagctc*GACTCGTTGGGCAAAGTGAAG-3′ (*Sac*I)	Deletion of *DAO1*
DAO1L-Br	5′-AAA*ggatcc*GGAAGCGCACAAAGTCAATTC-3′ (*Bam*HI)	Deletion of *DAO1*
DAO1R-Hf	5′-TTT*aagctt*CAAAGGAGAAGGAGGTGACA-3′ (*Hin*dIII)	Deletion of *DAO1*
DAO1R-Str	5′-TTT*aggcct*GTCTATTTGCGGTGGAATGGA-3′ (*Stu*I)	Deletion of *DAO1*

^a^Sequences in lowercase and italics denote the recognition site for the restriction enzyme used (marked in brackets)

BLAST search using *R. toruloidesDAO1* as query identified several *DAO1* homologs from *Pucciniomycotina* and *Ustilagiomycotina* subphyla (Additional file 2A). These genes were predicted to contain 2–7 introns although the *Ustilago maydis* homolog appeared intron-less. Interestingly, members in the *Ustilagiomycotina* subphyla divide *Pucciniomycotina* into two subgroups, one consisting of *Rhodosporidium*, *Rhodotorula* and *Sporobolomyces* and the other consisting of *Puccinia* and *Melampsora* (Additional file 2B).

### Regulation of *DAO1* mRNA transcription

**Fig. 3:**
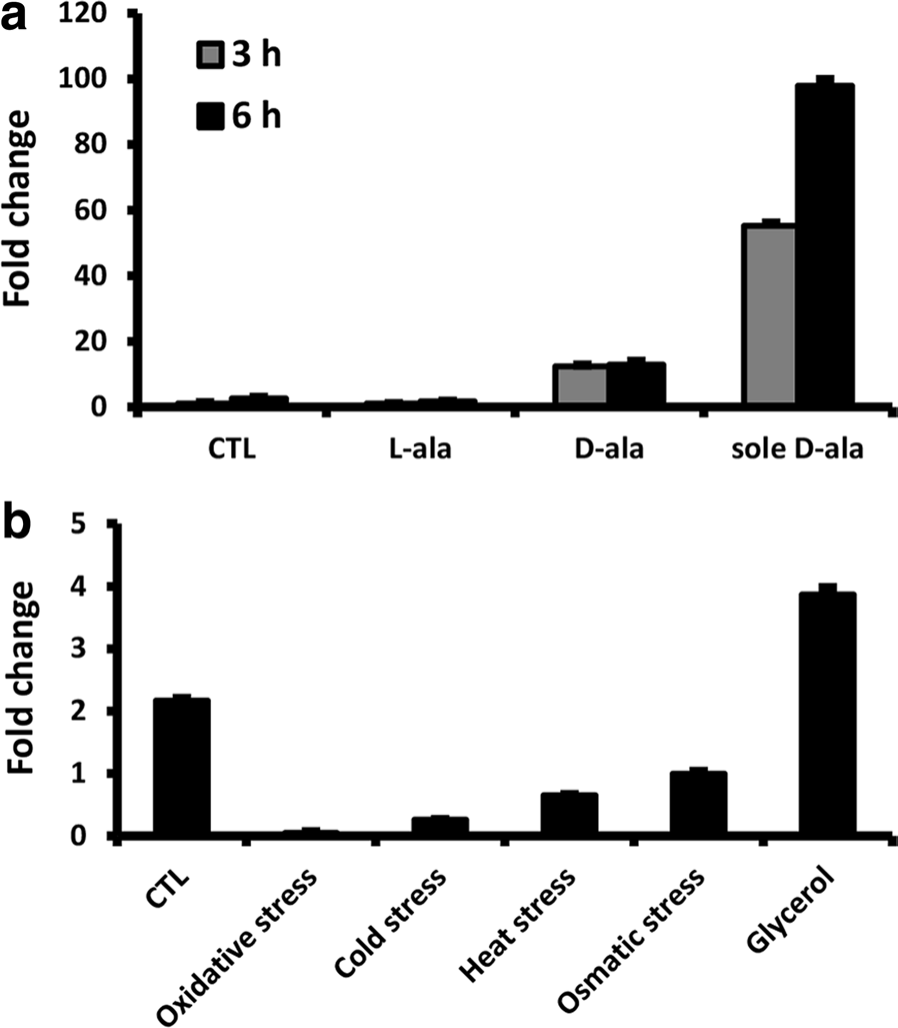
Transcription of native *DAO1* mRNA in *R. toruloides*. **a** Relative mRNA levels at 3rd and 6th hour when cells were cultured in MinABs medium supplemented with d- or l-alanine (70 mM). Carbon and nitrogen sources used: CTL—glucose and ammonium sulfate; l-ala—glucose and l-alanine; d-ala—glucose and d-alanine; sole d-ala—d-alanine only. The mRNA level at 6th hour in medium using d-alanine as the sole carbon and nitrogen source is set as 100 %. **b** Relative mRNA in cells cultured under various stress for 6 h. CTL: YPD broth at 28 °C; oxidative stress: YPD broth supplemented with 1 % H_2_O_2_ (w/v) and cultured at 28 °C; cold stress: YPD broth at 4 °C; heat stress: YPD broth at 37 °C; osmatic stress: YPD broth supplemented with 1 M KCl and cultured at 28 °C; glycerol: YPG broth (carbon source of glucose in YPD replaced with the same concentration of glycerol) and cultured at 28 °C

We compared *DAO1* mRNA levels in media supplemented with d-alanine and l-alanine by qRT-PCR. As expected, the mRNA transcripts were negligible when cells were cultured in minimal medium (MinABs) supplemented with l-alanine or ammonium sulfate as the sole nitrogen source (Fig. 3a). After 3 and 6 h of induction with 70 mM d-alanine, the native *DAO1* mRNA level was 12 and 18 folds higher than that of cells cultured with 70 mM l-alanine, respectively. Transcription was induced ~100 folds if both glucose and ammonium sulfate were omitted in the culture medium. These results suggest that the induction of *DAO1* expression is specific to d-alanine and carbon source (glucose) and inorganic nitrogen source strongly suppress mRNA transcription although glycerol appeared to be slightly stimulatory. In addition, *DAO1* transcription was found depressed by several stress stimuli (Fig. 3b).

### Bioinformatic analyses of *cis*-acting elements in *DAO1* promoter

Sequence analysis of *DAO1* upstream region revealed one putative CAAT box (CCAAT) at −645 from the translational start codon. No TATA box could be found near the transcriptional start point (tsp). However, a 15-nt pyrimidine-rich region (−44 to −30) (*ct* box) is located immediately upstream of tsp (Additional file 1).

**Fig. 4:**
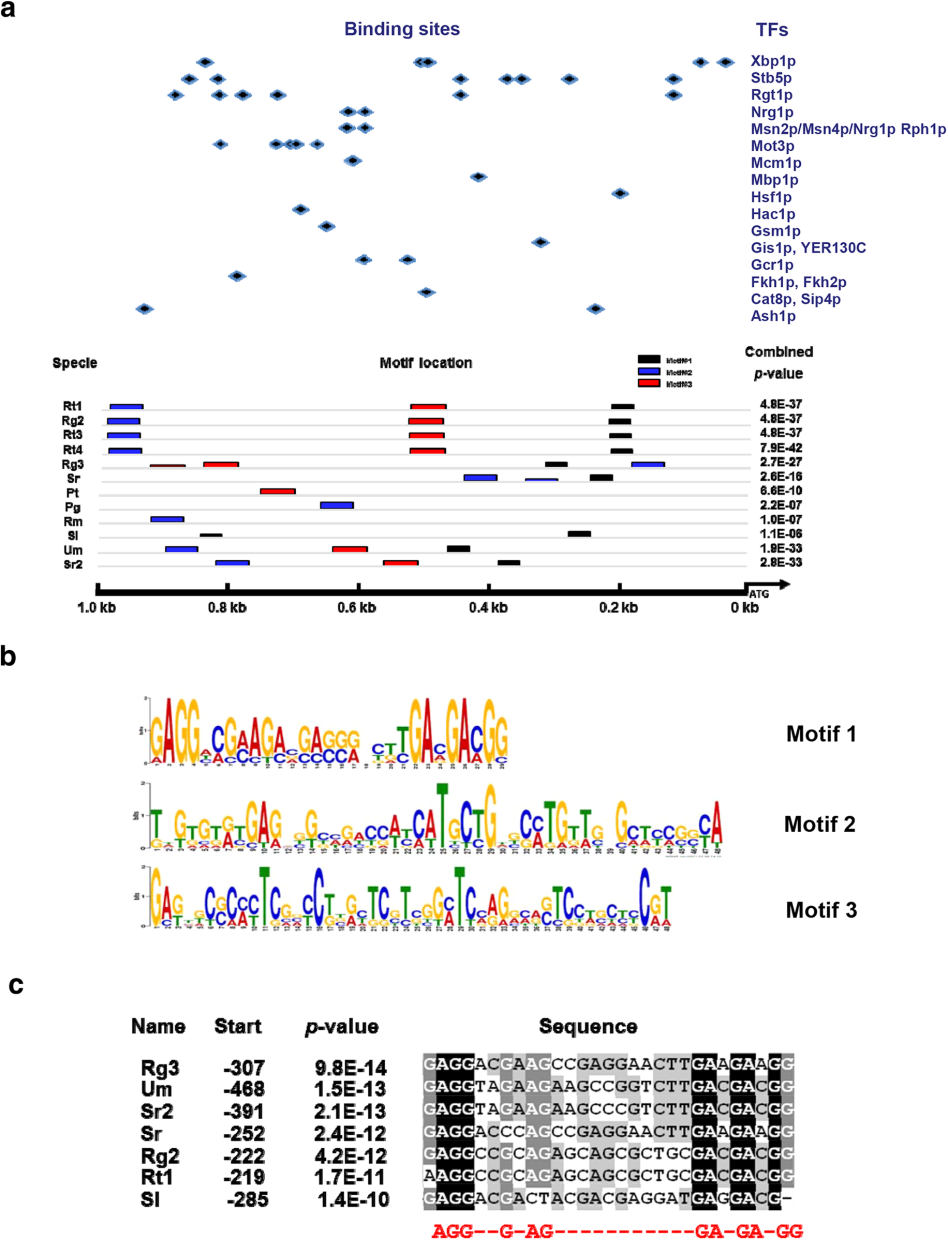
Analysis of upstream sequences of *DAO1* in *Pucciniomycotina*. **a** Localization of conserved DNA motifs in 1.0 kb upstream regions. Different transcription factor recognition sites were predicted based on the yeast transcription factor database YEASTRACT (http://www.yeastract.com). **b** Nucleotide sequence logos of the 3 DNA motifs indicated above. **c** Alignment motif 1 sequences of *Pucciniomycotina*. Abbreviations: *Rt1* *R. toruloides* ATCC 10657, *Rt3* *R. toruloides* MTCC 457, *Rt4* *R. toruloides* NP11, *Rg2* *R. glutinis* ATCC 204091, *Rg3* *R. graminis* WP1, *Sr* *S. roseus*, *Pt* *P. tritartic*, *Pg* *P. graminis*, *Rm* *R. minuta*; *Sl* *S. linerdae*, *Um* *U. maydis*, *Sr2* *S. reilianum*

Scanning the ~1.0 kb *DAO1* upstream sequences of several *Rhodosporidium* and *Rhodotorula* strains at YEASTRACT (Yeast Search for Transcriptional Regulators And Consensus Tracking, http://www.yeastract.com/) [22] identified several potential transcription factor binding sites with functions in stress response (Gis1p, Hac1p, Hsf1p, Mot3p, Msn2p/4p, Stb5p and Xbp1p), carbon source catabolism (Cat8p/Sip4p, Gcr1p, Nrg1p, Rgt1p), DNA synthesis (Mbp1p) and transcription repression (Ash1p) (Fig. 4a). In addition, analysis of upstream sequences (1.0 kb) of *DAO1* gene from several basidiomycetous fungi at the MEME server (http://meme.nbcr.net/meme/) identified three conserved sequence motifs (Fig. 4b). Motif 1 has a consensus sequence of AGGXXGXAGX_10_GAXGAXGG (where X represents any nucleotide) and is the most conserved amongst *Rhodsporidium* and *Rhodotorula* species (Fig. 4b, c).

### Functional dissection of *DAO1* promoter using luciferase reporter

**Fig. 5:**
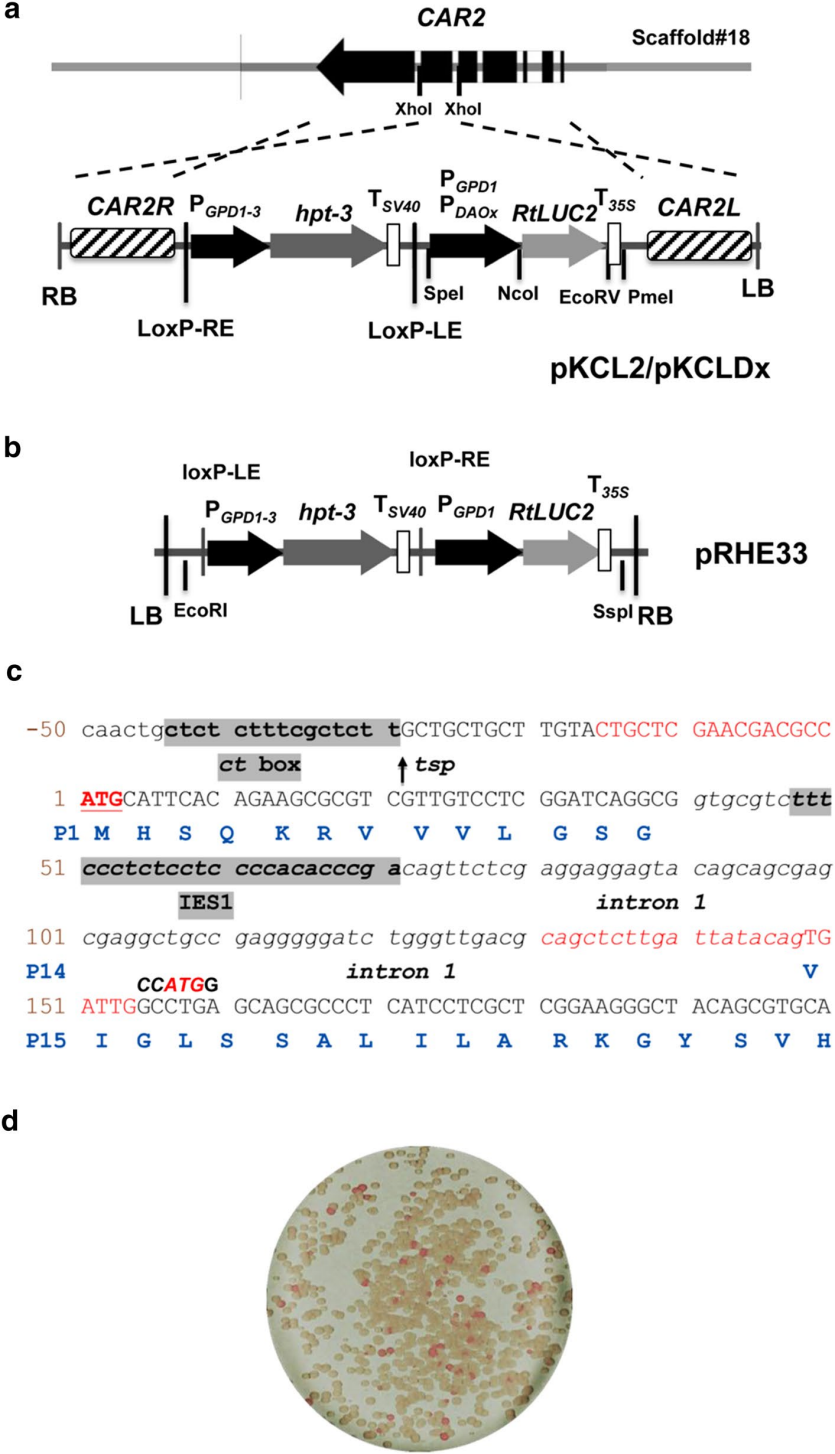
Rt*LUC2* reporter system. **a** Schematic diagram of *DAO1* gene structure and the T-DNA regions of reporter plasmid pKCL2 and pKCLDx. RB and LB: right and left borders *Agrobacterium tumefaceins* T-DNA; *CAR2L* and *CAR2R*: left and right homology arms of CAR2 locus that were used for locus-directed integration of Rt*LUC2* reporter constructs; P_*GPD1*-*3*_: *GPD1* promoter of *Rhodotorula graminis* WP1; *hpt*-3: codon-optimized hygromycin resistance gene; Rt*LUC2*: codon-optimized Luciferase gene; *loxP*-RE and *loxP*-LE: mutant recognition sites for Cre reccombinase [41], T_*35S*_: transcriptional terminator of *Cauliflower mosaic virus* gene 35S; *DAOx* indicates various *DAO1* promoter fragments in the reporters; **b** T-DNA regions of binary vector pRHE33. **c** Important features of the *DAO1* mRNA transcription. *tsp* transcriptional start point; Terminal sequence of P_*DAO1*-*in1*_ promoters are shown in the *box*. **d** Typical colony color phenotypes of pKCL2/pKCLDx transformants

Reporter assays were performed to define the minimal DNA sequence required to drive the d-amino acid inducible gene expression. *DAO1* upstream DNA fragments consisting of a series of nested deletions and site-specific mutations were used for gene reporter assay. Previously, a codon-adapted green fluorescent protein-encoding gene (Rt*GFP*) was used for reporter assay in *R. toruloides* using a large pool of randomly integrated T-DNA transformants [6]. Although effective, we found that the Rt*GFP* reporter is prone to background interference and showed large batch-to-batch variations (unpublished data). To overcome these problems, we used a codon-optimized firefly luciferase gene, Rt*LUC2* (GenBank accession number ACH53166), to compare the strength of various *DAO1* promoter fragments. Luciferase reporter cassettes were integrated at the *CAR2* locus using the ∆ku70e strain, which allows highly efficient site-specific integration of the reporter cassette [7]. As *CAR2* encodes a bifunctional enzyme phytoene synthase and lycopene cyclase that are essential for the biosynthesis of carotenoids in *R. toruloides*, site-specific integration at the locus lead to albino colony phenotype (Fig. 5) [7].

**Fig. 6:**
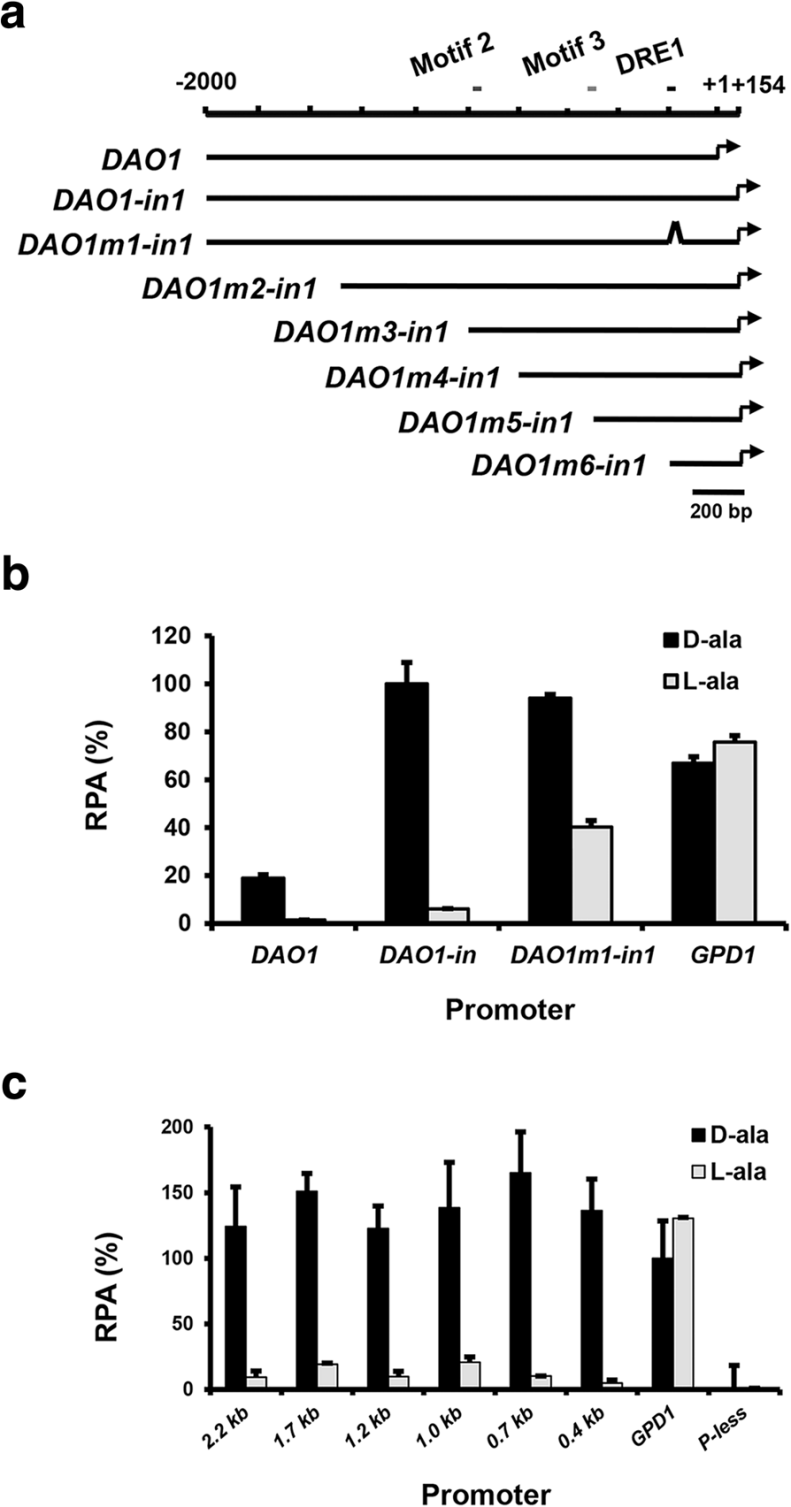
Functional dissection of *DAO1* promoter. **a** Schematic diagram of serial deletions of *DAO1* promoter knocked in at *CAR2* locus. **b** Effect of motif 1 and intron 1. Promoter activities were assayed with luciferase kit using cells cultured for 21 h in MinABs medium supplemented with d-alanine or l-alanine. **c** Relative promoter activities (RPA) of various deletions. *GPD1* promoter refers to the 795-bp promoter of glyceraldehyde-3-phosphate dehydrogenase gene. *P-less* promoter-less background control

Interestingly, inclusion of intron 1 in the reporter increased the promoter activity about fivefold, and this was independent of the presence of inducer d-alanine (Fig. 6a, b, compare P_*DAO1*_*and* P_*DAO1*-*in1*_). Deletion of the conserved motif 1 (DRE1) had little effect on the promoter strength when cultured under inducing conditions. In contrast, motif 1 deletion resulted in a 6.7-fold increase in promoter activity when cultured under non-inducing condition using l-alanine as sole nitrogen and carbon source (Fig. 6a, b, compare P_*DAO1m1*-in1_ and P_*DAO1*-in1_). This suggests motif 1 repressed *DAO1* expression under the non-inducing condition. Thus, we termed motif 1 as “d-amino acid responsive element 1 (DRE1)”. Notably, *DAO1* promoter drove stronger Rt*Luc2* expression than *GPD1* [6] did under non-induction conditions (Fig. 6b). An additional series of nested deletions of the 2.2 kb *DAO1*-*in1* promoter revealed that the 0.7 and 0.4 kb fragments were comparable to the full-length 2.2 kb promoter in terms of strength and stereospecificity (Fig. 6c).

**Fig. 7:**
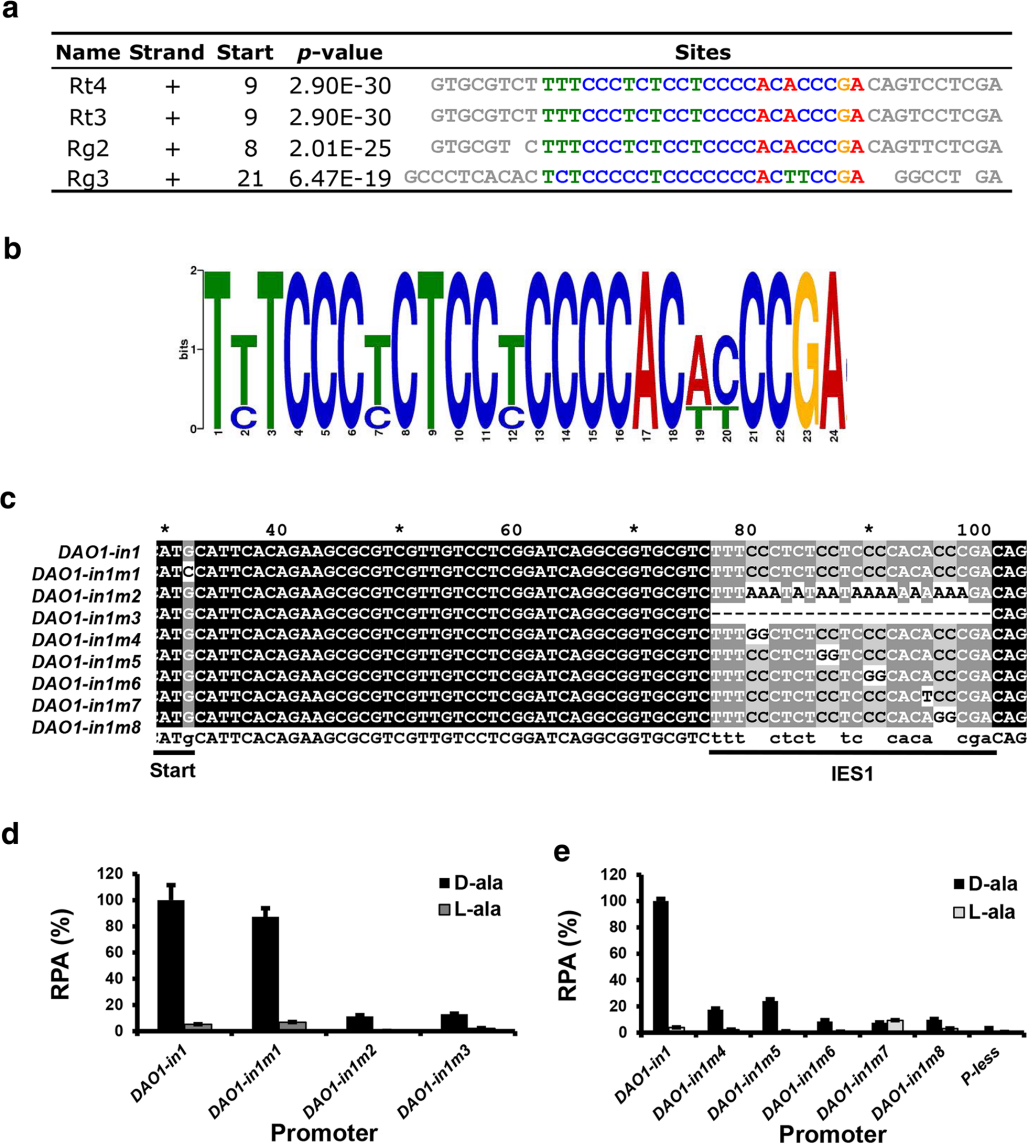
Functional characterization of intron 1 sequences of *DAO1*. **a** Alignment of the conserved DNA motifs in *DAO1* intron 1 of *Rhodospordium* and *Rhodotorula* species. **b** Nucleotide sequence logos of the DNA motifs shown above. *Rt3* *R. toruloides* MTCC 457, *Rt4* *R. toruloides* NP11, *Rg2* *R. glutinis* ATCC 204091, *Rg3* *R. graminis* WP1. **c** Sequences of various IES1 mutants used in P_*DAO1*-*in1m2*~*9*_. Translational start site and IES1 motif are *underlined*. **d**, **e** Relative strength of *DAO1* promoter mutants shown in **c**

As intron 1 of *DAO1* gene strongly enhanced the promoter activity, we sought to identify the *cis*-acting element involved. Analysis of the *DAO1* intron 1 sequences of several *Rhodosporidium*/*Rhodotorula* species at MEME server identified a conserved 24-bp CT-rich motif (Fig. 7a) with a consensus sequence of T(T/C)TCCC(T/C)CTCC(T/C)CCCCAC(A/T)(C/T)CCGA, which we named as intronic enhancer sequence 1 (IES1) (Fig. 7b). To demonstrate the function of IES1, seven mutants containing complete deletion or 1–2 nt mutations in IES1 were made in the 0.7 kb *DAO1m5*-*in1* promoter (Figs. 6, 7c). All above mutant promoters showed severely reduced strength when either d or l-alanine was used as the sole carbon and nitrogen source (Fig. 7d, e). These results support our assumption that IES1 is a general transcriptional enhancing element. Because the inclusion of intron 1 in the promoter added a 16-aa peptide to the N-termini of proteins expressed, we sought to create an improved promoter that yields clean protein translation, with no fusion of foreign peptide. We mutated the original translation initiation codon ACGCCATGC located in exon 1 to ACGCCATCC and re-created a new translation start codon (ATG) by changing the “TTGGCCTGA” sequence at the start of exon 2 to “TTGCCATGG”, which contains an *Nco*I site that allows seamless fusion of coding sequence of interest with the promoter. The promoter was named P_*DAO1*-*in1m1*_. Results confirmed that the modifications had little effect on the promoter strength and selectivity (Figs. 5c, 7d).

### Medium optimization for d-amino acid inducible gene expression

**Fig. 8:**
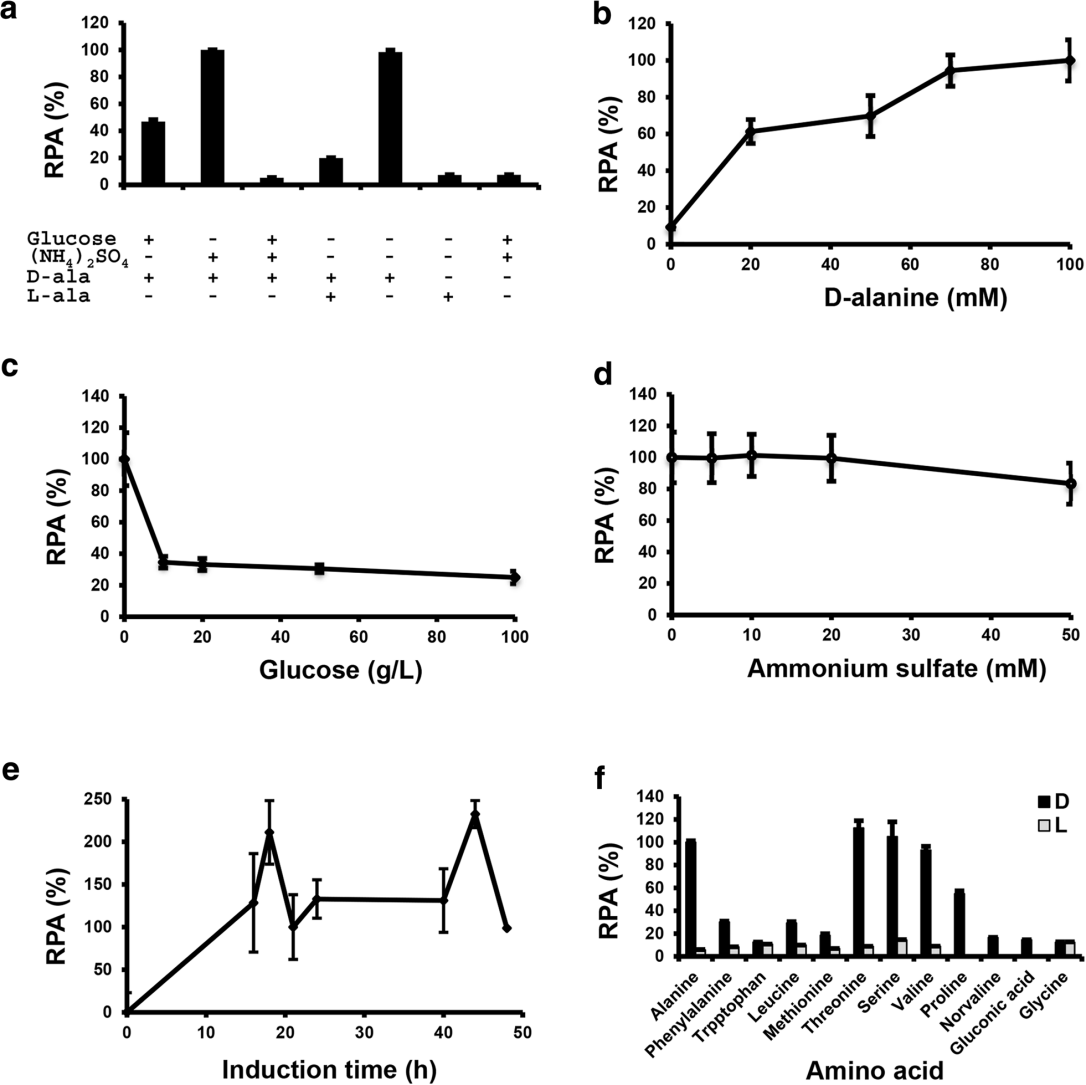
Effects of carbon and nitrogen sources on *DAO1* promoter activity. All assays were done in triplicates with a luciferase reporter strain containing the 2.2-kb P_*DAO1*-*in1*_ fused to Rt*LUC2* :T_*35S*_ and knocked in at *CAR2* locus. **a** Effect of carbon and nitrogen sources. Cells were cultured for 21 h in MinABs supplemented with the designated carbon and nitrogen sources. *G* glucose (10 g/L), *AS* ammonium sulfate (70 mM), *d* *-ala* d-alanine (70 mM), *l* *-ala* l-alanine (70 mM). **b** Effects of different concentrations of d-alanine. 10 g/L glucose and different concentrations of d-alanine were supplemented to the basal medium MinABs. **c** Effects of different concentrations of glucose. 70 mM d-alanine and different concentration of d-alanine were supplemented to basal medium MinABs. **d** Effects of different concentrations of ammonium sulfate. 10 g/L glucose, 70 mM d-alanine and different concentrations of ammonium sulfate were supplemented to basal medium MinABs. **e** Time course of promoter activities. 70 mM d-alanine was used as the sole carbon and nitrogen source. **f** Effects of different d-amino acids as the inducer. d and l represents d- and l-alanine, respectively

With the creation of luciferase reporter system, factors that influence gene expression could be identified. We chose strain KCLD2, which contains a copy of the 2.2-kb *DAO1*-*in1* promoter linked to Rt*LUC2* inserted into the *CAR2* locus, for medium optimization. Cells were cultured in MinABs medium supplemented with glucose and/or ammonium sulfate. The strongest luciferase expression was observed when 70 mM d-alanine was used as the sole carbon and nitrogen sources. l-alanine was a potent antagonist. An equal concentration of l-alanine reduced luciferase activity about fivefold at 21 h after induction when induction was made with 70 mM d-alanine (Fig. 8a). Ammonium sulfate (70 mM) alone had little effect while glucose at 10 g/L reduced the expression by half. Supplementation of both glucose (10 g/L) and ammonium sulfate (70 mM) led to a drastic reduction of promoter activity. d-alanine as low as 20 mM was effective in inducing the expression of luciferase although increasing the concentration further enhanced the expression (Fig. 8b). Glucose (10–100 g/L) and ammonium sulfate (5–50 mM) supplementation resulted in only marginal reduction of promoter activity (Fig. 8c, d).

The luciferase activity appeared to display two peaks, at about 18 and 44 h after induction (Fig. 8e). d-threonine, d-serine and d-valine were similarly effective while d-proline, d-leucine, d-phenylalanine, d-tryptophan and d-methionine were significantly less effective (Fig. 8f) [23].

### *DAO1* deletion further improved the inducible gene expression system

**Fig. 9:**
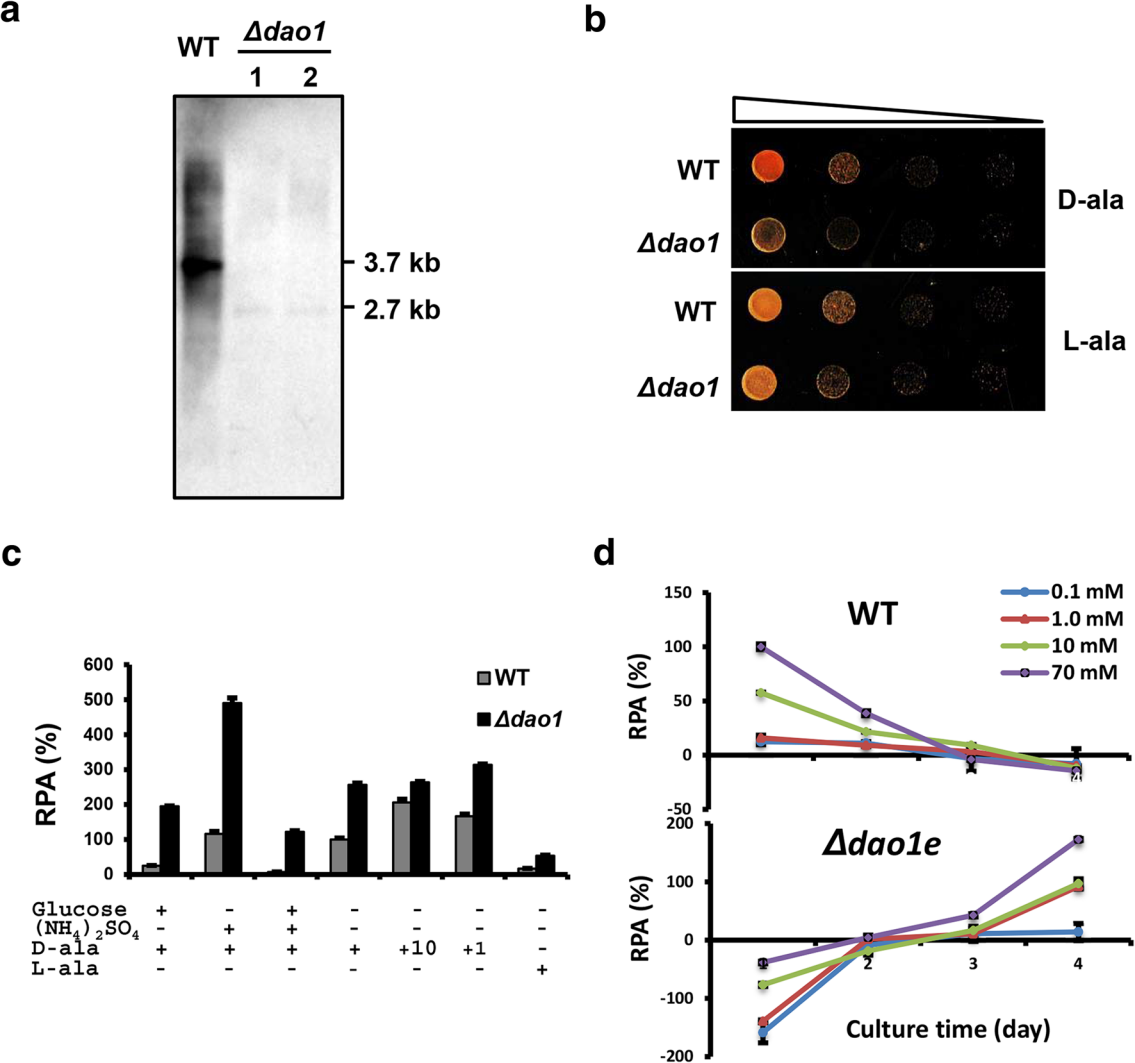
Effects of *DAO1* gene deletion. **a** Southern blot analysis of *DAO1* knockout mutants. Genomic DNA (2 µg) was digested with *Pst*I and hybridized against the digoxigenin-labeled probe of *DAO1R* (Probe 2 in Fig. 2a). **b** Growth of *DAO1* null mutant and WT in YNB medium with d-alanine and l-alanine as the sole carbon source. **c** Response of 2.2 kb P_*DAO1in1*_ in WT and *DAO1* knockout mutant (Δdao1e) in MinABs supplemented with glucose (10 g/L), ammonium sulfate (70 mM), l-alanine (l-ala, 70 mM). (+) indicates 70 mM d-alanine while other concentrations used are marked with +10 (mM) and +1 (mM), respectively. **d** Time course of 2.2 kb P_*DAO1*-*in1*_ activity in WT and Δdao1e strains. Strains were cultured in MinABs medium supplemented with various concentrations of d-alanine indicated

As Dao1 degrades d-amino acid inducers over time, we sought to test if *DAO1* mutation would improve the inducibility and stability of the expression system. We created a *DAO1* knockout mutant (∆dao1) in the *R. toruloides* Δku70e strain, in which the hygromycin selection cassette was removed by activating the *Cre*/*loxP* recombination system pre-integrated in the genome (our unpublished data). Two clean knockout mutants (∆dao1) were obtained and verified by Southern blotting (Fig. 9a). As expected, *DAO1* null mutant showed noticeable growth defect when cultured in medium with d-alanine as the sole carbon source. However, the defect was much milder when cultured in medium with l-alanine as the sole carbon source (Fig. 9b). The ∆dao1 strain grew essentially like wild-type in complete media such as YPD and Y4 medium (data not shown). Similarly, we deleted the selection cassette by activating the *Cre*/*lox*P system and the resultant strain (∆dao1e) showed significantly higher luciferase activity than wildtype when the P_*DAO1*-*in1*_::Rt*LUC2* cassette was inserted into the *CAR2* locus (Fig. 9c). Mostly importantly, the 2.2 kb *DAO1*-*in1* promoter showed much higher luciferase activity in ∆dao1e than wildtype background in several conditions tested (Fig. 9c). In media with high concentrations of both glucose and ammonium sulfate, *DAO1* deletion increased the reporter gene expression 17 folds when induced with 70 mM d-amino acid (Fig. 9c).

To demonstrate the application of *DAO1*-*in1* promoter in lipid production stage, strains KCLD2 and KCLD21 (with P_*DAO1*-*in1*_::Rt*LUC2* integrated at the *CAR2* locus in WT and ∆dao1e, respectively), were cultured in the lipid accumulation medium Y4 that allows high cell density culture and high oil accumulation [24]. While the promoter showed deceasing activity over the 4-day culture period in wildtype, it showed a steady increase of activity in ∆dao1e background (Fig. 9d), which appeared to be concomitant with carbon and nitrogen depletion (Additional file 3A and 3B). Interestingly, gene expression was repressible with d-alanine in ∆dao1e background during the initial 2-day culture. Importantly, effective induction could be achieved with d-alanine in levels as low as 1 mM. These results indicate that the *DAO1*-*in1* promoter is a useful tool for inducible expression of genes under lipid production conditions, particularly when a *DAO1* null mutant is used. The strength of gene expression could be tuned by adjusting the levels of inducer, carbon source and nitrogen source.

## Discussion

Promoters are key element for gene expression systems, either for modulation of biosynthetic pathways or production of recombinant proteins. Carbon source and nitrogen source regulated promoters have been widely used in fungi [25–27], for example, galactose-inducible promoters have been very successful in *Saccharomyces cerevisiae* [28]. While putative *GAL1*, *GAL4*, *GAL7* and *GAL10* homologs could be found in *Rhodosporidium* and *Rhodotorula* genomes, none of the promoters were effectively induced by galactose (data not shown).

The lack of an effective inducible gene expression system had been a major hurdle in making *Rhodosporidium* and *Rhodotorula* a competitive host for metabolic engineering and synthetic biology. This report specifically addresses this issue. Luciferase gene reporter assays of systemically truncated and site-specific mutations of *DAO1* upstream DNA fragments allowed us to engineer a d-amino acid inducible promoter that is robust and simple to use. The promoter can be as small as 0.4 kb and, after inactivating the original translation initiation codon in exon 1 and re-creating a new ATG is exon 2. Based on the ribosome scanning model [29], protein translation could be initiated only at TTGCCATGG in the mutant promoters, such as P_*DAO1*-*in1m1*_, and the protein produced should be free of unwanted peptide at the N-terminus because there is no other ATG triplet in the 5′ UTR in any possible frames. The D-amino acid inducible gene expression system can be enhanced by using a *DAO1* null mutant (∆dao1e), which allowed much stronger induction in media with carbon source and/or nitrogen source (Fig. 9). With the inactivation of degradation system for the d-amino acid inducer, ∆dao1e strain allowed significantly improved gene induction with much lower concentration of inducer needed and thus, reduced cost for its applications. We expect similar inducible gene expression system can be created for other fungi because Dao proteins are highly conserved. The d-amino acid inducible gene expression system reported here should find applications metabolic pathways, genome editing and enzyme expression in *Rhodosporidium* and *Rhodotorula* species and beyond.

CAAT box, TATA box and CT box are considered the “core promoter elements” of fungal promoters [30]. The GCCCAATCA motif (−647 to −638) shares high similarity to CAAT box consensus sequence (GCc/tCAATCT) found in eukaryotic promoter although it is located more distal to the transcriptional start point (tsp) than most fungal promoters. Similar to many genes of filamentous fungi [30], a CT-rich motif (CTCTCTTTCGCTCTT) is located immediately upstream of transcriptional start point of *DAO1* mRNA (Fig. 5 and Additional file 1). On the other hand, no TATA box in the proximity of tsp nor canonical polyadenylation signal (AATAAA) near the transcriptional termination site was found. Similarly to *DAO1*, *R. toruloides GPD1*, *KU70* and *KU80* genes also contain no TATA box or canonical polyadenylation signal [6, 7]. Because *Rhodosporidium* and *Rhodotorula* genomes are amongst the most GC-rich known, with more than 62 % CG content [31], it is intriguing how *cis*-acting elements and the corresponding transcription factors have been evolved during the evolution of these organisms. To the best of our knowledge, DRE1 and IES1 are the first functionally verified *cis*-acting elements reported in *Pucciniomycotina* to date. The location of an enhancing element in intron 1 was unexpected. Recently, intron 1 of *Yarrowia lipolyticaFBA1* gene was also reported to strongly enhance gene expression [32]. These suggest that intronic gene transcriptional enhancer may be more common than we currently know. The positive (IES1) and negative (DRE1) *cis*-acting elements identified here will be useful for engineering promoters with lower basal transcriptional level and stronger mRNA transcriptional activity using strategies that have been extensively applied in other eukaryotic organisms [33–35].

We noticed the basal expression level under non-inducing conditions (examples see l-alanine in Figs. 6b, 8a) remained high. It is possible that alanine racemase (EC 5.1.1.1, ALR), which converts l-alanine to d-alanine, played a role because a putative homolog of *Schizosaccharomyces pombe* alanine racemase (GenBank acc. no. AL023590) [34] could be found in the *R. toruloides* genome. This gene could be a target for further improvement of d-amino acid inducible expression in *Rhodosporidium* and *Rhodotorula.*

## Conclusion

The intron 1 containing *DAO1* promoter coupled with a *DAO1* null mutant makes an efficient and tight d-amino acid-inducible gene expression system in *Rhodosporidium* and *Rhodotorula* genera. The system will be a valuable tool for metabolic engineering and enzyme expression in these yeast hosts.

## Methods

### Strains, media, and culture conditions

*R. toruloides* strain ATCC 10657 was obtained from ATCC (USA). ∆ku70e is a derivative of *KU70* knockout mutant with hygromycin selection cassette removed by activation of Cre recombinase and allows highly efficient gene deletion by homologous recombination [7]. *R. toruloides* strains were cultured at 28 °C in YPD broth (1 % yeast extract, 2 % peptone, 2 % glucose, w/v) or on YPD agar. *A. tumefaciens* strain AGL2 [36] was cultured at 28 °C in either 2YT broth or 2YT agar medium (1.6 % tryptone, 1 % yeast extract, 0.5 % NaCl). *Escherichia coli* XL1-Blue was cultured in Luria–Bertani (LB) broth or on LB agar and used for routine DNA manipulations.

For gene induction studies, chemically-defined minimal medium MinAB [37] without carbon source and nitrogen source, named as MinABs here, was used as the basal medium, which was supplemented with carbon source and nitrogen source when desired. Unless indicated otherwise, cells were cultured in 250 ml flasks or 50 ml Falcon tubes with agitation (250 rpm) at 28 °C. For lipid accumulation, medium Y4 containing 100 g/L glucose, 15.7 g/L peptone, 15.7 g/L yeast extract, 12 g/L (NH4)_2_SO_4_, 1 g/L KH_2_PO_4_, 1.5 g/L MgSO_4_·7H2O (pH5.5) was used [24].

### Plasmid constructs

Oligonucleotides used are listed in Table 1. All DNA restriction and modification enzymes were sourced from New England Biolabs (NEB, USA). Plasmid pKCL2 (Fig. 5a) is a pPZP200 derivative [38] consisting of a hygromycin resistant cassette (P_*GPD1*-*3*_::*HPT*-*3*::T_*SV40*_) and a luciferase reporter cassette (P_*GPD1*_:Rt*LUC2::*T_*35S*_). P_*GPD1*-*3*_ and P_*GPD1*_ are the glyceraldehyde 3-phosphate promoter of *R. graminis* WP1 and *R. toruloides* ATCC 10657, with GenBank accession number of JQ806386 and JN208861, respectively [6]. *HPT*-*3* (JQ806387) and Rt*LUC2* (KR258785) are the codon-optimized synthetic genes encoding the *E. coli* hygromycin phosphotransferase and firefly luciferase (Luc2, ACH53166.1), respectively [6].

pKCL2 allows efficient site-specific integration of reporter gene cassette at the *CAR2* locus (Fig. 5a). To make this, a 2321 kb genomic DNA fragment of *CAR2* (396,844–399,540 nt of scaffold #18, AEVR02000018) was amplified by PCR using genomic DNA of *R. toruloides* ATCC 10657 as template and oligos Rt079 and C250r as primers. The PCR products were treated with T4 polynucleotide kinase and ligated with the *Sac*I (blunt-ended)/*Pme*I-double digested pEX2 plasmid [39] to create the intermediate plasmid pEX2CAR2kc. The 8.6-kb *Xho*I-linearized and blunt-ended pEX2CAR2kc was ligated with the P_*GPD1*-*3*_::*hpt*-*3::*T_*SV40*_-P_*GPD1*_:Rt*GFP::*T_*35S*_ double gene cassettes obtained by digestion of pRHE33 (Fig. 5b) with *Eco*RI and *Ssp*I followed by blunt-ending using T4 DNA polymerase in the presence of dNTPs. The Rt*GFP* gene in pKC2 was replaced with the synthetic 1.7 kb *Nco*I-*Eco*RV fragment of Rt*LUC2* to create pKCL2.

The 2.0 kb upstream region of *DAO1* was obtained by PCR amplification using oligos Rt290Sf and Rt309Nr as primers (Table 1), yielding a PCR fragment with 5′ *Spe*I and 3′ *Nco*I cutting sites, the latter of which (CCATGG) overlaps with the original predicted translational codon (ATG) of *DAO1*. The PCR products were double-digested with *Spe*I and *Nco*I before inserted into same sites of pKCL2 to create pKCLD1 (Fig. 5b). The 2.2 kb intron 1-containing promoter P_*DAO1*-*in1*_ was amplified with oligos Rt290Sf and Rt287Nr as primers. The *Spe*I–*Nco*I treated PCR products of P_*DAO1*-*in1*_ were inserted at the same sites in pKCL2 to create plasmid pKCLD2 (Fig. 5b). P_*DAO1*-*in1*_ includes the 108-nt intron 1 and 6-nt exon 2 sequence, with which a 16-aa peptide (MHSQKRVVVLGSGVIA) will be added to the N-terminus of any protein expressed (Fig. 5c).

The 2.2 kb P_*DAO1m1*-*in1*_ promoter (Fig. 6a), which has the d-amino acid responsive element 1 (DRE1) deleted, was created by fusion PCR: DNA sequence upstream and downstream of DRE1 was individually amplified using pKCLD2 as the template and oligo pair SFGFPSEQ/Rt312 and Rt311/35STer as primers, yielding PCR fragments of 2.1 and 1.3 kb in length, which were used as PCR templates at a molar ratio of 1:1 to make the DRE1-deleted 2.2 kb P_*DAO1m1*-*in1*_ promoter using oligos Rt290Sf and Rt287Nr as primers. After double-digestion with *Spe*I and *Nco*I, P_*DAO1m1*-*in1*_ fragment was inserted to pKCL2 at the same sites to create pKCLD3 (Fig. 5b). A promoter-less Rt*LUC2* reporter construct, pKCL20, was made by self-ligation of *Spe*I–*Nco*I digested and blunt-ended pKCL2.

Truncated promoters of approximately 1.7 kb (P_*DAO1m2*-*in1*_), 1.2 kb (P_*DAO1m3*-*in1*_), 1.0 kb (P_*DAO1m4*-*in1*_), 0.7 kb (P_*DAO1m5*-*in1*_) and 0.4 kb (P_*DAO1m6*-*in1*_) in length were amplified using oligo Rt287Nr as reverse primer and Rt315S, Rt314S, Rt120S, Rt313S and Rt117S as forward primer, respectively (Table 1). All PCR products were double-digested with *Spe*I and *Nco*I and followed by insertion to pKCL2 at the same sites to create plasmid pKCLD4 to pKCLD8, respectively (Figs. 5b, 6).

Promoter P_*DAO1m5*-*in1m1*_ contains the ATG to ATC point mutation at the translation initiation codon and “TTGGCCTGA” to “TTGCCATGG mutations in exon 2 (Fig. 5c). This shifts the translation initiation codon to exon 2, with the translation of first 16-aa peptide of Dao1 abolished. Oligo pair SFGFPSEQ/Rt327r and Rt328f/LUC2U were used amplify the 0.8 and 0.4 kb DNA mutant fragments, which were fused by PCR using oligos of Rt313S and Rt287Nr. The resultant 0.7 kb PCR product was digested with *Spe*I–*Nco*I and inserted to pKCL2 at the same sites to make pKCLD71. Similarly, oligo pair SFGFPSEQ/Rt329r was used to amplify the 0.8 kb upstream DNA fragment that was shared for P_*DAO1m5*-*in1m2*_ to P_*DAO1m5*-*in1m8*_ while the downstream fragment was amplified using the common reverse primer Luc2U coupled with forward primer Rt330f, Rt331f, Rt334f, Rt335f, Rt336f, Rt337f and Rt338f, respectively. The above PCR fragments were fused by PCR using oligos Rt313S and Rt287Nr followed by double digestion with *Spe*I and *Nco*I before insertion into pKCL2 to create plasmid pKCLD72 to pKCLD79, respectively (Fig. 5b).

*DAO1* knockout vector pKODAO1 was created first by amplifying the 5′ and 3′ flanking sequence (0.6 kb each) using oligo pair DAO1L-Sf/DAO1L-Br and DAO1R-Hf/DAO1R-Str as the primers. The PCR fragments were assembled by four-fragment ligation, consisting of *Sac*I/*Bam*HI-cut 5′ homology arm, *Hin*dIII/*Stu*I-cut 3′ homology arm, *Hin*dIII/*Bam*HI cut hygromycin resistance cassette obtained from pDX1PgpdRhptR [7] and *Sac*I/*Pme*I-cut dephosphorylated pEX2tk [39].

### Extraction of genomic DNA and total RNA

Genomic DNA was extracted using the MasterPure-Yeast DNA and RNA Purification Kits, respectively (Epicenter, USA). The concentrations of DNA or RNA samples were determined with NanoDrop^®^ ND-1000 Spectrophotometer (Nanodrop Technologies, USA) and the integrity of the extracted nucleic acids were checked by agarose gel electrophoresis. DNA and RNA concentrations of the samples were typically 100–1000 ng/µL.

### Rapid amplification of cDNA ends (RACE)

The 5′ and 3′ end of *DAO1* cDNA sequences were obtained by 5′ RACE and 3′ RACE using BD SMARTer™ RACE cDNA Amplification Kit (Clontech, USA) according to the manufacturer’s instruction. Oligos DAO1U and DAO1L (Table 1) were used as the specific primer for 5′ RACE and 3′ RACE, respectively. The full-length cDNA was cloned by RT-PCR using DNase I-treated total RNA as the template and Rt332 and Rt333 as the primers.

### Southern blot analysis

Genomic DNA (5 µg) was digested with restriction enzymes, separated in 0.8 % agarose gel and blotted to Hybond N^+^ membrane (GE Healthcare Life Sciences, USA). DNA probes were labeled with DIG High Prime DNA Labeling and Southern hybridization and detection were performed according to manufacturer’s instruction (Roche Diagnostics, USA).

### Quantitative reverse transcription PCR (qRT-PCR)

Total RNA was treated with DNase I (Roche Diagnostics, Germany) followed by precipitation with ethanol. cDNA was synthesized using the iScript™ Reverse Transcription Supermix for reverse transcription (Bio-Rad, USA) and real-time PCR was conducted in ABI PRISM 7900HT Sequence Detection System (Life Technologies, USA) using the ABI SYBR^®^ Select Master Mix (Life Technologies, USA). Real-time PCR conditions were as followed: an initial 50 °C for 2 min and 95 °C denaturation for 10 min followed with 40 cycles of denaturation at 95 °C for 15 s, annealing and amplification at 60 °C for 1 min. qRT-PCR analysis was done with biological triplicates. Data was acquired using the software SDS 2.4 (Life Technologies, USA) and the relative gene expression levels were calculated against the reference gene *ACT1* (GenBank accession number KR138696) using the 2^−∆∆Ct^ method facilitated with the RQ Manager software v1.2.1 (Life Technologies USA).

### Analysis of promoter activity

Binary T-DNA vectors were electroporated into *Agrobacterium tumefaciens* AGL2 and *A. tumefaciens*-mediated transformation (ATMT) of *R. toruloides* was performed as previously described [6]. Strains bearing the inserted T-DNA at the *CAR2* locus were identified by the albino phenotype followed by confirmation with Southern blotting.

Luciferase reporter strains were cultured in YPD broth to mid-exponential phase. Cells were washed twice with water and inoculated to various media at an optical density (OD_600_) of 0.5 and cultured at 30 °C with agitation (250 rpm). Cell cultures (2 mL) were harvested, washed and re-suspended in PBS buffer supplemented with 1 mM DTT, 3 mM β-ME and 1 mM PMSF (pH7.4). After addition of equal volume of glass beads (0.5 mm in diameter, Sigma-Aldrich, USA), cells were ruptured in a bead beater (FastPrep-24™ 5G, MP Biomedicals, Solon, OH, USA) with two cycles of beating (45 s) and cooling in ice-water bath (5 min). After centrifugation (4 °C, 14,000 rpm for 15 min), the supernatants were collected as crude enzyme preparation and total protein contents were determined by the Bradfort method [40] using Quick Start™ Bradford Protein Assay Kit (Bio-Rad, USA) with bovine serum albumin (BSA) as the standard.

Luciferase activity was determined by bioluminescence using Luciferase Assay System (Promega, USA). All data were measured and acquired with the Infinite M200 plate reader coupled with the iCycler software (version 3.0) (Tecan, Austria). Protein concentrations were measured at OD of 595 nm with 5 µL of samples or protein standard, which were mixed with 250 µl 1× dye reagent (Bio-Rad) and loaded to wells in a 96-well flat-bottom transparent plate (Nunc, Denmark). Luminescent values were measured after mixing 10 µL of samples with 100 µL of Luciferase Assay Reagent (Promega) in wells of a FluoroNunc 96-well plate (Thermo Fisher Scientific, Germany). The relative promoter activity (RPA) was calculated as followed: RPA = [(L_T_ − L_BLK_)/P_T_ − (L_N_ − L_BLK_)/P_N_]/[(L_P_ − L_BLK_)/P_P_ − (L_N_ − L_BLK_)/P_N_], where L_T_, L_N_, L_P_ and L_BLK_ represents the bioluminescent value of target promoter, promoter-less, full-length promoter and PBS buffer blank, respectively, and P_T_, P_P_ and P_N_ represents the protein concentration of the enzyme preparation derived from strain with the target promoter, full-length promoter and promoter less, respectively.

### GenBank accession numbers

All *DAO1* genes together with their 2 kb upstream sequences from Basidiomycotina, i.e. *Rhodosporidium toruloides* ATCC 10657, *Rhodotorula glutinis* ATCC 204091, *Rhodotorula graminis* WP1, *Rhodosporidium toruloides* MTCC 457, *Rhodosporidium toruloides* NP11, *Sporobolomyces reseus*, *Puccinia tritartica*, *Puccinia graminis*, *Melampsora laricis*-*populina*, *Rhodotorula minuta*, *Sporobolomyces linderae*, *Ustilagomaydis*, *Sporisoriumreilianum*, were deposited to GenBank under accession numbers KR183638-183695, respectively (Additional file 1). Actin encoding gene (*ACT1*) of *R. glutinis* ATCC 204091 is under GenBank accession number KR183696 (Additional file 5). The synthetic Rt*LUC2* gene (GenBank accession number KR258785) was made by Genscript, USA. All sequences are available in Additional files 4, 5, 6.

## Additional file

10.1186/s12934-015-0357-7  Structure of *R. toruloides DAO1* gene. Nucleotide positions are defined from the translational start codon “ATG” (in which the position of “A” is +1). Protein positions are labeled with the prefix “P”. The translational start codon within *Nco*I site (CCATGG) in the intron 1-containing promoters are and the ATG to ATC mutation are boxed. Exons are shown in capital letters and introns are in lowercase italics; the putative CAAT box is highlighted in grey; DRE1, *ct* box and IES1 are indicated with shaded text. The putative coenzyme binding consensus sequence (GXGXXG, where “X” is any amino acid) is underlined; the dimerization loop is double underlined; the amino acid residues H-bonded to the flavin O-2 atom (YQ) are in bold and italics; TG repeats upstream of the polyA site are highlighted in grey; the residues interacting with the -carboxy and -amino groups of the substrate (Y_223_, Y_238_ and R_285_) are boxed and highlighted in grey; the C-terminus peroxisomal targeting signal (SKL) is highlighted in grey. Abbreviations: *ct* box: pyrimidine-rich region; tsp: transcriptional start point; PolyA site: polyadenylation site.
10.1186/s12934-015-0357-7  Basidiomycetous *DAO1 *genes and phylogenic analysis of their proteins. (a) Schematic diagrams of *DAO1 *genes from *Pucciniomycotina *and *Ustilagiomycotina *subphyla. Introns are shown in white bars. (b) Phylogenetic tree analysis of putative D-amino acid oxidases from *Pucciniomycotina *and *Ustilagiomycotina *subphyla. The phylogenic tree was constructed by MEGA version 6 program (http://www.megasoftware.net/) using Neighbor-Joining algorithm and tested by Bootstrapping. The *DAO1 *sequences are listed in GenBank under accession numbers KR183638-183695.
10.1186/s12934-015-0357-7 Residual carbon and nitrogen sources during lipid accumulation. *R. toruloides* was cultured in the lipid accumulation medium Y4 [23] with some modifications  (see Materials and Methods) for 5 days. (a) Residual ammonium concentration in cell culture. (b) Residual glucose concentration in cell culture.
10.1186/s12934-015-0357-7  Codon usage in *R. toruloides DAO1*.
10.1186/s12934-015-0357-7 *DAO1 *gene sequences (Submitted GenBank sequence record ahead of release). (a) List of sequence ID, organisms and GenBank accession numbers of *DAO1 *and *ACT1 *genes. (b) Genome sequences of *DAO1 *genes (start at -2000). (c) Protein sequences of Dao1.
10.1186/s12934-015-0357-7 Sequence of the mutation promoter P_*DAO1m5-in1m1 *_(renamed as P_*DAO1int*_).

## Abbreviations

ATCC: American Type Culture Collection, USA
DAO1: d-amino acid oxidase gene
*GPD1*: glyceraldehyde 3-phosphate dehydrogenase gene
Rt*GFP*: a commercially synthesized green fluorescent protein (eGFP) gene according to the codon bias of *R. toruloides*
Rt*LUC2*: a commercially synthesized luciferase (Luc2) encoding gene according to the codon bias of *R. toruloides*
*CAR2*: bifunctional enzyme phytoene synthase and lycopene cyclase encoding gene
BLAST: Basic Local Alignment Search Tool (National Library of Medicine, National Institutes of Health, USA)
FAD: flavin adenine dinucleotide
MEME server: motif-based sequence analysis tools (National Institutes of Health, USA)
DRE1: d-amino acid responsive element 1
ISE1: intronic enhancer sequence 1
