# Erratum to: Engineering an efficient and tight d-amino acid-inducible gene expression system in *Rhodosporidium/Rhodotorula* species

**DOI:** 10.1186/s12934-017-0726-5

**Published:** 2017-06-14

**Authors:** Yanbin Liu, Chong Mei John Koh, Si Te Ngoh, Lianghui Ji

**Affiliations:** Biomaterials and Biocatalysts Group, Temasek Life Sciences Laboratory, 1 Research Link, National University of Singapore, Singapore, 117604 Singapore

## Erratum to: Microb Cell Fact (2015) 14:170 DOI 10.1186/s12934-015-0357-7

Unfortunately, the original article [1] contains two errors that are being corrected via this erratum.

1. In Fig. 9d legend, the culture medium used should be corrected from “MinABs” to “Y4” supplemented with various concentrations of d-alanine.
2. In the ‘Methods’ section, the GenBank accession numbers “KR183638-183695” should be corrected to “KR138683-138695”, and “KR183696” should be corrected to “KR138696”.
