# Supplementary material for: Engineering an efficient and tight d-amino acid-inducible gene expression system in Rhodosporidium/Rhodotorula species
Source: Microb Cell Fact. 2015 Oct 26;14:170. doi: 10.1186/s12934-015-0357-7 (PMC4624585; doi:10.1186/s12934-015-0357-7)
Supplement: Supplementary file 1 — 10.1186/s12934-015-0357-7 Structure of R. toruloides DAO1 gene. Nucleotide positions are defined from the translational start codon “ATG” (in which the position of “A” is +1). Protein positions are labeled with the prefix “P”. The translational start codon within NcoI site (CCATGG) in the intron 1-containing promoters are and the ATG to ATC mutation are boxed. Exons are shown in capital letters and introns are in lowercase italics; the putative CAAT box is highlighted in grey; DRE1, ct box and IES1 are indicated with shaded text. The putative coenzyme binding consensus sequence (GXGXXG, where “X” is any amino acid) is underlined; the dimerization loop is double underlined; the amino acid residues H-bonded to the flavin O-2 atom (YQ) are in bold and italics; TG repeats upstream of the polyA site are highlighted in grey; the residues interacting with the -carboxy and -amino groups of the substrate (Y223, Y238 and R285) are boxed and highlighted in grey; the C-terminus peroxisomal targeting signal (SKL) is highlighted in grey. Abbreviations: ct box: pyrimidine-rich region; tsp: transcriptional start point; PolyA site: polyadenylation site. [file 12934_2015_357_MOESM1_ESM.pdf]

```
-1000   ggtctgatcg cttgtgtgcg agaggtcgac catcatgccg agcctgttca gctccggtac aagctcgcgc ccgaaggcgg tcaggccgtt cccgtcgtgt

-900    acaggctcga taggagagcc gtccccggcg gaagaggcga aggctgtgag aaaaacccgt caacagagcg gaagaaggcc gagaggtcgc ctcgcctcac

-800    aagtgtggca ggtgtgcgtg agcgtaaggt accgcacgcc gagttgctga aagaggcgta ggatggccag cgagttcatc aggtgatgcg agctgtagga

-700    agagcgggtt agcgggagga tgtcggtaga gactgttcgt acaaacccct ctagcccaat caaggacgcg atcttcccct ccgcgaacgc actccgcacc
                                                                 CAAT box
-600    tcgtccgccg ttcgagcgag cgccatctcc tcaggatagt gctccaccat ccgatgaata aggtcgacgc tctcgagtgc ccactcgacc gcgttcgtgg

-500    gctcaaggaa gtcctccccc gtcggctgcg catcacacgg agcatacgca acatggaaca agcctcctac gcgtccttga cgcaacttcg ggaggtcgac

-400    atggcctgca agaccggtcg caagttcagg gaggacgtcg agcggtctcc ggcagaggtg gcgagcgacg taggggaggt cgacgtgtcc gtctatgagt

-300    gggtggcgtt tcaggatccc gcgggcctca gcgagcaagg actcgttggg caaagtgaag gtgtactgat gagcggttgc gaggccgcag agcagcgctg
                                                                                                             DRE1
-200    cgacgacggg aagcttcggc acgagcatga ctgtgagtag tagtccaagg agaacagcgc agagtcggca ggagggcaca tggaggcaga gcgtggggcg

-100    gaggaggcag atggggagtc gcgctggggg acgagagggt gccgctcgac caactgctct ctttcgctct tGCTGCTGCT TGTACTGCTC GAACGACGCC
                                                                     ct box     ↑ tsp
        C
  1     ATGCATTCAC AGAAGCGCGT CGTTGTCCTC GGATCAGGCG gtgcgtcttt ccctctcctc cccacacccg acagttctcg aggaggagta cagcagcgag
 P1      M  H  S  Q   K  R  V   V  V  L   G  S  G                CCATGG →     IES1
101     cgaggctgcc gagggggatc tgggttgacg cagctcttga ttatacagTG ATTGGCCTGA GCAGCGCCCT CATCCTCGCT CGGAAGGGCT ACAGCGTGCA
P14                                                   V   I  G  L  S   S  A  L   I  L  A   R  K  G  Y   S  V  H
201     CATTGTGGCC CGCGACTTGC CGGAGGACGT CTCGAGCCAG ACTTTCGCTT CGCCGTGGGC TGTAGGTCGT CTCGTTCGGG CTCTCAGATG TCCCTGTGAG
P32       I  V  A   R  D  L  P   E  D  V   S  S  Q   T  F  A  S   P  W  A
301     CTGACCACTT CCATGTGTGA CGCAGGGCGC GAACTGGACG CCGTTCATGT CTCTCACGGA CGGTCCTCGA CAAGCAAAGT GGGAAGAATT GACTTTgtgc
P52                           G  A   N  W  T   P  F  M  S   L  T  D   G  P  R   Q  A  K  W   E  E  L   T  L
401     gcttcccttc tgtgtctctt tttcacttcg tgctaaccaa gctcgtgcta cacagCAAGA AGTGGGTCGA GCTTGTACCG ACCGGCCAGG TCATGTGGCT
P76                                                          K   K  W  V  E   L  V  P   T  G  Q  V   M  W  L
501     CAAGGGGACG AGGCGGTTCG CGCAGAACGA GGATGGCTTG CTCGGGCATT GGTACAAGGA CATCACGCCC AACgtgggtt caccgctagt tctgctgctc
P91       K  G  T   R  R  F  A   Q  N  E   D  G  L   L  G  H  W   Y  K  D   I  T  P   N
601     ctcaacctca ccaggtcact gacgaccctc cttccgtcgc acgcagTACC GCCCCCTCCC TTCCTCCGAA TGTCCCCCCA ACTCGATCGG CGTAACCTAC
P115                                                  Y  R   P  L  P   S  S  E   C  P  P  N   S  I  G   V  T  Y
701     GACACCCTCT CCGTCCACGC ACCAAAGTAC TGCCAGTACC TCGCGAGGGG ATTACAGAAG CTTGGCGCGA CGTTTGAGAG ACGGACGGTC ACGTCGGTTG
P133     D  T  L  S   V  H  A   P  K  Y   C  Q  Y  L   A  R  G   L  Q  K   L  G  A  T   F  E  R   R  T  V   T  S  V
801     AGCAGGCGTT CGAGGGGGTG GATTTGGTGG TCAACGCTAC GGGTCTCGgt gcgtcgtacc ttgtctctca tgcgtcaagc agcttactaa cagtcctctc
P167    E  Q  A  F   E  G  V   D  L  V  V   N  A  T   G  L
901     gcagGCGCCA AGTCGATCGC TGGCATCGAC GACCAAGCCG CCGAGCCAAT CCGCGGTCAA ACCGTCCTCG TCAAGTCCGC CTGCAAGCGC TGCACGATGG
P182       G  A  K   S  I  A   G  I  D   D  Q  A  A   E  P  I   R  G  Q   T  V  L  V   K  S  A   C  K  R   C  T  M
1001    ACTCGTCCGA CCCCTCTTCT CCCGCGTATA TCATCCCACG ACCAGGTGGC GAAGTGATCT GCGGCGGGAC GTACGGCGTG GGCGATTGGG ACTTGTCCGT
P215    D  S  S  D   P  S  S   P  A  Y   I   I  P  R   P  G  G   E  V  I  C   G  G  T   Y  G  V   G  D  W  D   L  S
1101    CAACCCTGAG ACGGTCCAGC GGATCCTCAA GCACTGCCTG CGACTCGACC CGTCCATCTC GAGCGACGGC ACGATCGAGG GTATCGAAGT CCTCCGGCAC
P248    V N  P  E   T  V  Q  R   I  L  K   H  C  L   R  L  D  P   S  I  S   S  D  G   T  I  E  G   I  E  V   L  R  H
1201    AACGTTGGCT TGCGACCCGC ACGTCGAGGC GGCCCCCGCG TCGAGGCAGA ACGGCTCGTC CTCCCTCTCG ACCGGTCCAA ATCGCCCCTC TCGCTTGGCA
P281     N  V  G  L   R  P  A   R  R  G   G  P  R  V   E  A  E   R  L  V   L  P  L  D   R  S  K   S  P  L   S  L  G
1301    AGGGCACGAC AAGGGCGGCA AAGGAGAAGG AGGTGACACT CGTGCATGCG TATGGGTTCT CGAGCGCCGG GTACCAGCAG AGTTGGGGCG CGGCGGAGGA
P315    K  G  T  T   R  A  A   K   E  K  E   V  T  L   V  H  A   Y  G  F  S   S  A  G   Y  Q  Q   S  W  G  A   A  E  D
1401    TGTTGCGCTG CTTGTCGAGG AGGCGTTCCA GCGGTACCAC GGCGCTGCAA GGGAGTCGAA GCTGTAGGGC GAGGCTTGAG TGGCTGTATT GGGGCATTTC
P348      V  A  L   L  V  E  E   A  F  Q   R  Y  H   G  A  A  R   E  S  K   L  *
1501    ACCAGGTTCG CCGCgtatcg agacgagagt gtggcgaccc ttcgcaccgt ctgaccttcg caacccgtca actccttgcc ctttggcctg cttgcttcct
                    ↑ PolyA site
1601    cggtctctcc tggatttcgc aagctcgctc atcgacgctg tcactcgcca ggccatccct tcaagctgtc gtcgcccctt tcctcctcct cctccttctc
```

Addition file 1. Structure of *R. toruloides DAO1 gene.* Nucleotide positions are defined from the translation start codon "ATG" (in which the position of "A" is +1). Protein positions are labeled with the prefix "P". The translational start codon within *Nco*I site (CCATGG) in the intron 1-containing promoters are and the ATG to ATC mutation are boxed. Exons are shown in capital letters and introns are in lowercase italics; the putative CAAT box is highlighted in grey; DRE1, *ct* box and IES1 are indicated with shaded text. The putative coenzyme binding consensus sequence (GXGXXG, where "X" is any amino acid) is underlined; the dimerization loop is double underlined; the amino acid residues H-bonded to the flavin O-2 atom (**YQ**) are in bold and italics; **TG** repeats upstream of the polyA site are highlighted in grey; the residues interacting with the

-carboxy and -amino groups of the substrate (Y_{223}, Y_{238} and R_{285}) are boxed and highlighted in grey; the C-terminus peroxisomal targeting signal (**SKL**) is highlighted in grey. Abbreviations: *ct* box: pyrimidine-rich region; *tsp*: transcriptional start point; *PolyA site:* polyadenylation site.
