# Supplementary material for: Engineering an efficient and tight d-amino acid-inducible gene expression system in Rhodosporidium/Rhodotorula species
Source: Microb Cell Fact. 2015 Oct 26;14:170. doi: 10.1186/s12934-015-0357-7 (PMC4624585; doi:10.1186/s12934-015-0357-7)
Supplement: Supplementary file 2 — 10.1186/s12934-015-0357-7 Basidiomycetous DAO1 genes and phylogenic analysis of their proteins. (a) Schematic diagrams of DAO1 genes from Pucciniomycotina and Ustilagiomycotina subphyla. Introns are shown in white bars. (b) Phylogenetic tree analysis of putative D-amino acid oxidases from Pucciniomycotina and Ustilagiomycotina subphyla. The phylogenic tree was constructed by MEGA version 6 program (http://www.megasoftware.net/) using Neighbor-Joining algorithm and tested by Bootstrapping. The DAO1 sequences are listed in GenBank under accession numbers KR183638-183695. [file 12934_2015_357_MOESM2_ESM.pdf]

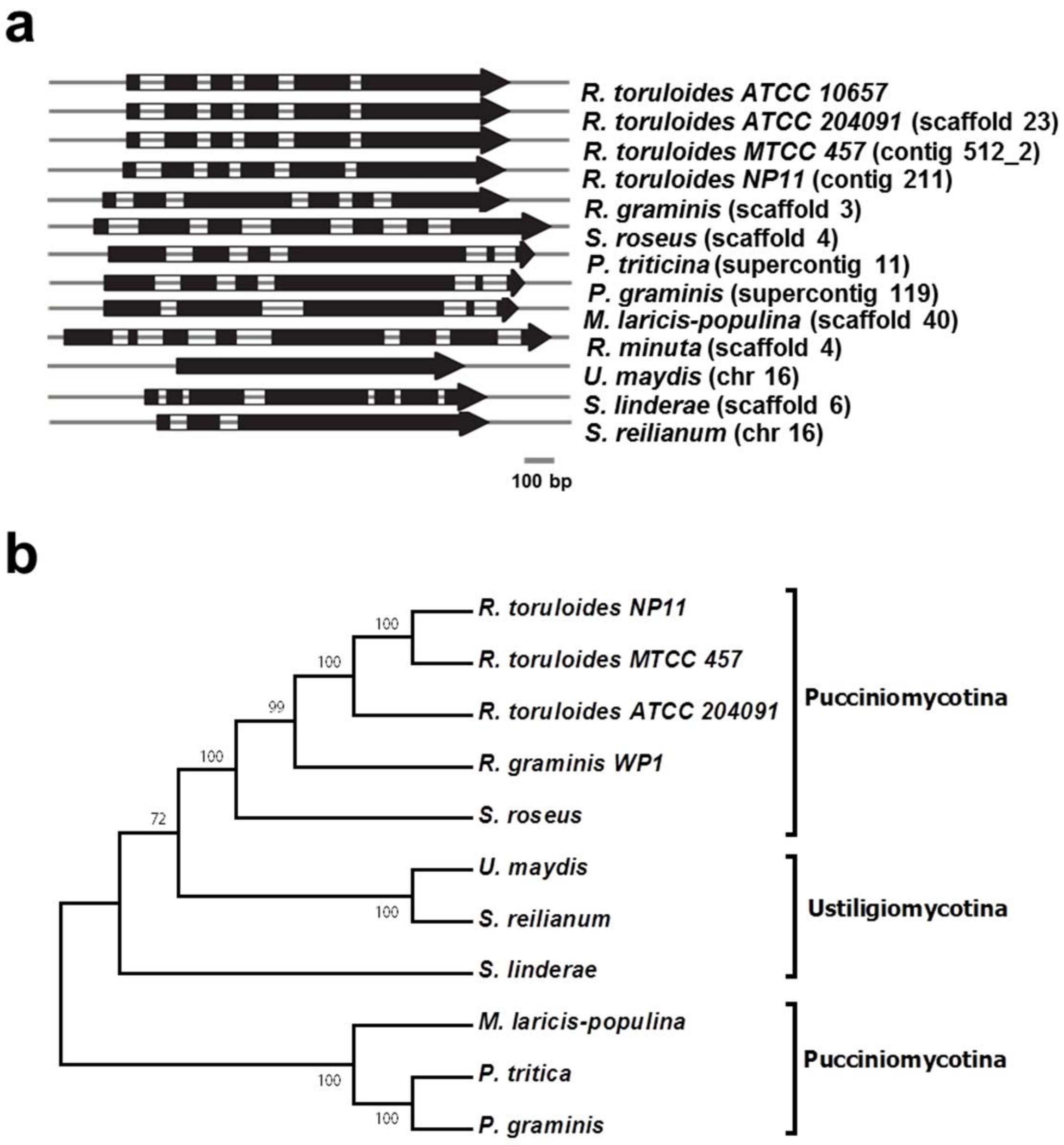

Additional file 2. Basidiomycetous *DAO1* genes and phylogenic analysis of their proteins. (a) Schematic diagrams of *DAO1* genes from *Pucciniomycotina* and *Ustilagiomycotina* subphyla. Introns are shown in white bars. (b) Phylogenic tree analysis of putative D-amino acid oxidases from *Pucciniomycotina* and *Ustilagiomycotina* subphyla. The phylogenic tree was constructed by MEGA version 6 program (http://www.megasoftware.net/) using Neighbor-Joining algorithm and tested by Bootstrapping. The *DAO1* sequences are listed in GenBank under accession numbers KR183638-183695.
