# Supplementary material for: Engineering an efficient and tight d-amino acid-inducible gene expression system in Rhodosporidium/Rhodotorula species
Source: Microb Cell Fact. 2015 Oct 26;14:170. doi: 10.1186/s12934-015-0357-7 (PMC4624585; doi:10.1186/s12934-015-0357-7)
Supplement: Supplementary file 3 — 10.1186/s12934-015-0357-7 Residual carbon and nitrogen sources during lipid accumulation. R. toruloides was cultured in the lipid accumulation medium Y4 [23] with some modifications (see Materials and Methods) for 5 days. (a) Residual ammonium concentration in cell culture. (b) Residual glucose concentration in cell culture. [file 12934_2015_357_MOESM3_ESM.pdf]

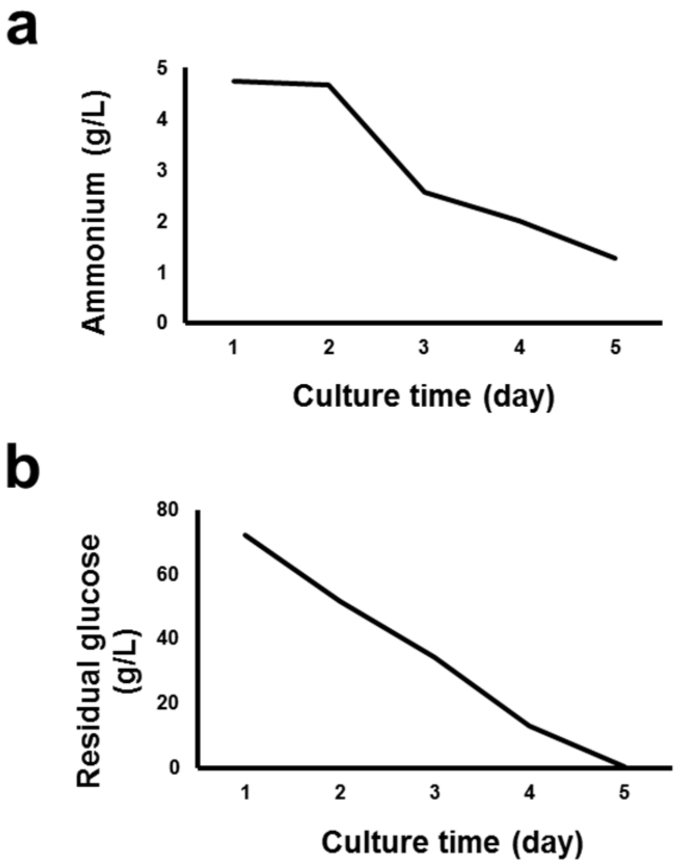

Additional file 3. Residual carbon and nitrogen sources during lipid accumulation. *R. toruloides* was cultured in the lipid accumulation medium Y4 with some modifications [23] for 5 days. (a) Residual ammonium concentration in cell culture. (b) Residual glucose concentration in cell culture.
