# Supplementary material for: Engineering an efficient and tight d-amino acid-inducible gene expression system in Rhodosporidium/Rhodotorula species
Source: Microb Cell Fact. 2015 Oct 26;14:170. doi: 10.1186/s12934-015-0357-7 (PMC4624585; doi:10.1186/s12934-015-0357-7)
Supplement: Supplementary file 4 — 10.1186/s12934-015-0357-7 Codon usage in R. toruloides DAO1. [file 12934_2015_357_MOESM4_ESM.pdf]

**Additional file 4. Codon usage in *R. toruloides DAO1***

| | | | | | | | | | | | |
|---|---|---|---|---|---|---|---|---|---|---|---|
| TTA [a] | L [b] | 0.3 [c] | TCA | S | 0.5 | TAA | - | 0.0 | TGA | - | 0.0 |
| TTC | F | 1.9 | TCC | S | 2.2 | TAC | Y | 2.4 | TGC | C | 1.4 |
| TTG | L | 1.6 | TCG | S | 3.0 | TAG | - | 0.3 | TGG | W | 2.2 |
| TTT | F | 0.3 | TCT | S | 0.8 | TAT | Y | 0.5 | TGT | C | 0.3 |
| CTA | L | 0.0 | CCA | P | 1.1 | CAA | Q | 0.8 | CGA | R | 1.4 |
| CTC | L | 5.1 | CCC | P | 2.4 | CAC | H | 1.4 | CGC | R | 3.5 |
| CTG | L | 1.1 | CCG | P | 1.4 | CAG | Q | 3.0 | CGG | R | 2.2 |
| CTT | L | 1.1 | CCT | P | 1.1 | CAT | H | 0.8 | CGT | R | 0.3 |
| ATA | I | 0.0 | ACA | T | 0.5 | AAA | K | 0.3 | AGA | R | 0.3 |
| ATC | I | 3.5 | ACC | T | 1.1 | AAC | N | 1.9 | AGC | S | 1.6 |
| ATG | M | 1.1 | ACG | T | 3.5 | AAG | K | 4.6 | AGG | R | 1.1 |
| ATT | I | 5.4 | ACT | T | 0.5 | AAT | N | 0.0 | AGT | S | 0.3 |
| GTA | V | 0.5 | GCA | A | 1.6 | GAA | E | 1.6 | GGA | G | 0.5 |
| GTC | V | 4.3 | GCC | A | 1.9 | GAC | D | 3.5 | GGC | G | 6.0 |
| GTG | V | 2.4 | GCG | A | 3.3 | GAG | E | 4.3 | GGG | G | 1.6 |
| GTT | V | 1.1 | GCT | A | 1.6 | GAT | D | 1.1 | GGT | G | 1.4 |

[a] Triplicate codon.
[b] Amino acid residue.
[c] Frequency of codon usage (%).
