# Supplementary material for: Engineering an efficient and tight d-amino acid-inducible gene expression system in Rhodosporidium/Rhodotorula species
Source: Microb Cell Fact. 2015 Oct 26;14:170. doi: 10.1186/s12934-015-0357-7 (PMC4624585; doi:10.1186/s12934-015-0357-7)
Supplement: Supplementary file 5 — 10.1186/s12934-015-0357-7 DAO1 gene sequences (Submitted GenBank sequence record ahead of release). (a) List of sequence ID, organisms and GenBank accession numbers of DAO1 and ACT1 genes. (b) Genome sequences of DAO1 genes (start at -2000). (c) Protein sequences of Dao1. [file 12934_2015_357_MOESM5_ESM.pdf]

Additional file 5. *DAO1* gene sequences (Submitted GenBank sequence record ahead of release)

(a) List of sequence ID, organisms and GenBank accession numbers of *DAO1* and *ACT1* genes

Sequence_ID	Organism	GenBank acc. no.
Seq1	*Rhodosporidium toruloides* ATCC 10657 *DAO1*	KR138683
Seq2	*Rhodotorula glutinis* ATCC 204091 *DAO1*	KR138684
Seq3	*Rhodotorula graminis* WP1 *DAO1*	KR138685
Seq4	*Rhodosporidium toruloides* MTCC 457 *DAO1*	KR138686
Seq5	*Rhodosporidium toruloides* NP11 *DAO1*	KR138687
Seq6	*Sporobolomyces reseus DAO1*	KR138688
Seq7	*Puccinia tritartica DAO1 DAO1*	KR138689
Seq8	*Puccinia graminis DAO1 DAO1*	KR138690
Seq9	*Melampsora laricis-populina DAO1 DAO1*	KR138691
Seq10	*Rhodotorula minuta DAO1*	KR138692
Seq11	*Sporobolomyces linderae DAO1*	KR138693
Seq12	*Ustilago maydis DAO1*	KR138694
Seq13	*Sporisorium reilianum DAO1*	KR138695
Seq14	*Rhodotorula glutinis* ATCC 204091 *ACT1*	KR138696

(b) Genome sequences of *DAO1* genes (start at -2000)

>Seq1 [organism=Rhodosporidium toruloides ATCC 10657] D-amino acid oxidase gene, complete CDS
ggaagacatccccctcttcctcgctcagattgccgctctcctgcaaggccagcagtggggcgtcggtgcgtgcccaactcgtctgaagtggccaaatggctgaca
gtgctcccgcaggtcacgacgctgccgccatccttcgcggtcttcggcagcggaagctttgattctttcttgcctctctcggttgtacaatcgtcgtgctcgccagtttg
ttgttgatatccctagccttgccttccatactgaacaatctacaactcgtccttcttgcgcggcggaaagcgcttcagcacctcgttctgcacgtcttgcggcaagtact
ccccacccggtcctgcccacatcaccggcagatctgtccgcttctcatacactgctggtgaggcaggttgcgaggcgagagagcgctggaccgcctcgacttctt
ccattacgcgaaggaggttgccgccgatcaagtctgctaggtcgtcttccgtccatcctcgtcgcaacaactccgcaatctgcagagacgaggtagtcagcacatg
tcatagctgaagaagcaagcgatgcgcaccaagttcggaaacttcgacgcgtcctcaagcccctcaacactcgagcgcataccgtcaaagtcagacccgagacc
gacgtgcttcttgccgcagacggaggcgatgtactcgatatgatcggcgacacggatttgcgtggcgttcgtagggtcgataaaggaagggtagaagacgacca
ttctgcgagacgcgaaggtcagccggaaacttctgacgacaggaagtaacgtcgtacgcacacgatgccgtggttctggtgctcgcctggcccgatcagctgaa
gaacttcctctgggacgtttcgcgggtggtcgtggatcgctcgcgcgccagagtgcgagaagatgacgggcgcaacgctgagttcgagggcctgctcgcaaatc
gtcagctaaagctcgaaagtgttgaagcgcggtgcgcacgtcgagcatggtctgatcgcttgtgtgcgagaggtcgaccatcatgccgagcctgttcagctccgg
tacaagctcgcgcccgaaggcggtcaggccgttcccgtcgtgtacaggctcgataggagagccgtccccggcggaagaggcgaaggctgtgagaaaaaccc
gtcaacagagcggaagaaggccgagaggtcgcctcgcctcacaagtgtggcaggtgtgcgtgagcgtaaggtaccgcacgccgagttgctgaaagaggcgta
ggatggccagcgagttcatcaggtgatgcgagctgtaggaagagcgggttagcgggaggatgtcggtagagactgttcgtacaaacccctctagcccaatcaag
gacgcgatcttcccctccgcgaacgcactccgcacctcgtccgccgttcgagcgagcgccatctcctcaggatagtgctccaccatccgatgaataaggtcgacg
ctctcgagtgcccactcgaccgcgttcgtgggctcaaggaagtcctcccccgtcggctgcgcatcacacggagcatacgcaacatggaacaagcctcctacgcg
tccttgacgcaacttcgggaggtcgacatggcctgcaagaccggtcgcaagttcagggaggacgtcgagcggtctccggcagaggtggcgagcgacgtaggg
gaggtcgacgtgtccgtctatgagtgggtggcgtttcaggatcccgcgggcctcagcgagcaaggactcgttgggcaaagtgaaggtgtactgatgagcggttg
cgaggccgcagagcagcgctgcgacgacgggaagcttcggcacgagcatgactgtgagtagtagtccaaggagaacagcgcagagtcggcaggagggcac
atggaggcagagcgtggggcggaggaggcagatggggagtcgcgctgggggacgagagggtgccgctcgaccaactgctctctttcgctcttgctgctgcttg
tactgctcgaacgacgccatgcattcacagaagcgcgtcgttgtcctcggatcaggcggtgcgtctttccctctcctccccacacccgacagttctcgaggaggag
tacagcagcgagcgaggctgccgagggggatctgggttgacgcagctcttgattatacagtgattggcctgagcagcgccctcatcctcgctcggaagggctac
agcgtgcacattgtggcccgcgacttgccggaggacgtctcgagccagactttcgcttcgccgtgggctgtaggtcgtctcgttcgggctctcagatgtccctgtga
gctgaccacttccatgtgtgacgcagggcgcgaactggacgccgttcatgtctctcacggacggtcctcgacaagcaaagtgggaagaattgactttgtgcgcttc
ccttctgtgtctctttttcacttcgtgctaaccaagctcgtgctacacagcaagaagtgggtcgagcttgtaccgaccggccaggtcatgtggctcaaggggacgag
gcggttcgcgcagaacgaggatggcttgctcgggcattggtacaaggacatcacgcccaacgtgggttcaccgctagttctgctgctcctcaacctcaccaggtc

actgacgaccctccttccgtcgcacgcagtaccgcccctcccttcctccgaatgtcccccaactcgatcggcgtaacctacgacaccctctccgtccacgcacc
aaagtactgccagtacctcgcgaggggattacagaagcttggcgcgacgtttgagagacggacggtcacgtcggttgagcaggcgttcgagggggtggatttgg
tggtcaacgctacgggtctcggtgcgtcgtaccttgtctctcatgcgtcaagcagcttactaacagtcctctcgcaggcgccaagtcgattgctggcatcgacgacc
aagccgccgagccaatccgcggtcaaaccgtcctcgtcaagtccgcctgcaagcgctgcacgatggactcgtccgacccctcttctcccgcgtatatcatcccac
gaccaggtggcgaagtgatctgcggcgggacgtacggcgtgggcgattgggacttgtccgtcaaccctgagacggtccagcggatcctcaagcactgcctgcg
actcgacccgtccatctcgagcgacggcacgatcgagggtatcgaagtcctccggcacaacgttggcttgcgacccgcacgtcgaggcggcccccgcgtcga
ggcggaacggctcgtcctccctctcgaccggtccaaatcgcccctctcgcttggcaagggcacgacaagggcggcaaaggagaaggaggtgacactcgtgca
tgcgtatgggttctcgagcgccgggtaccagcagagttggggcgcggcggaggatgttgcgctgcttgtcgaggaggcgttccagcggtaccacggcgctgca
agggagtcgaagctgtagggcgaggcttgagtggctgtattggggcatttcaccaggttcgccgc
>Seq2 [organism=Rhodotorula glutinis ATCC 204091] D-amino acid oxidase gene, complete CDS
ggaagacatcccccctcttcctcgctcagattgccgctctcctgcaaggccagcagtggggcgtcggtgcgtgcccaactcgtctgaagtggccaaatggctgaca
gtgctcccgcaggtcacgacgctgccgccatccttcgcggtcttcggcagcggaagctttgattctttcttgcctctctcggttgtacaatcgtcgtgctcgccagtttg
ttgttgatatccctagccttgccttccatactgaacaatctacaactcgtccttcttgcgcggcggaaagcgcttcagcacctcgttctgcacgtcttgcggcaagtact
ccccacccggtcctgcccacatcaccggcagatctgtccgcttctcatacactgctggtgaggcaggttgcgaggcgagagagcgctggaccgcctcgacttctt
ccattacgcgaaggaggttgccgccgatcaagtctgctaggtcgtcttccgtccatcctcgtcgcaacaactccgcaatctgcagagacgaggtagtcagcacatg
tcatagctgaagaagcaagcgatgcgcaccaagttcggaaacttcgacgcgtcctcaagcccctcaacactcgagcgcataccgtcaaagtcagacccgagacc
gacgtgcttcttgccgcagacggaggcgatgtactcgatatgatcggcgacacggatttgcgtggcgttcgtagggtcgataaaggaagggtagaagacgacca
ttctgcgagacgcgaaggtcagccggaaacttctgacgacaggaagtaacgtcgtacgcacacgatgccgtggttctggtgctcgcctggcccgatcagctgaa
gaacttcctctgggacgtttcgcgggtggtcgtggatcgctcgcgcgccagagtgcgagaagatgacgggcgcaacgctgagttcgagggcctgctcgcaaatc
gtcagctaaagctcgaaagtgttgaagcgcggtgcgcacgtcgagcatggtctgatcgcttgtgtgcgagaggtcgaccatcatgccgagcctgttcagctccgg
tacaagctcgcgcccgaaggcggtcaggccgttcccgtcgtgtacaggctcgataggagagccgtccccggcggaagaggcgaaggctgtgagaaaaaccc
gtcaacagagcggaagaaggccgagaggtcgcctcgcctcacaagtgtggcaggtgtgcgtgagcgtaaggtaccgcacgccgagttgctgaaagaggcgta
ggatggccagcgagttcatcaggtgatgcgagctgtaggaagagcgggttagcgggaggatgtcggtagagactgttcgtacaaacccctctagcccaatcaag
gacgcgatcttcccctccgcgaacgcactccgcacctcgtccgccgttcgagcgagcgccatctcctcaggatagtgctccaccatccgatgaataaggtcgacg
ctctcgagtgcccactcgaccgcgttcgtgggctcaaggaagtcctcccccgtcggctgcgcatcacacggagcatacgcaacatggaacaagcctcctacgcg
tccttgacgcaacttcgggaggtcgacatggcctgcaagaccggtcgcaagttcagggaggacgtcgagcggtctccggcagaggtggcgagcgacgtaggg
gaggtcgacgtgtccgtctatgagtgggtggcgtttcaggatcccgcgggcctcagcgagcaaggactcgttgggcaaagtgaaggtgtactgatgagcggttg
cgaggccgcagagcagcgctgcgacgacgggaagcttcggcacgagcatgactgtgagtagtagtccaaggagaacagcgcagagtcggcaggagggcac
atggaggcagagcgtggggcggaggaggcagatggggagtcgcgctgggggacgagagggtgccgctcgaccaactgctctctttcgctcttgctgctgcttg
tactgctcgaacgacgccatgcattcacagaagcgcgtcgttgtcctcggatcaggcggtgcgtctttccctctcctccccacacccgacagttctcgaggaggag
tacagcagcgagcgaggctgccgagggggatctgggttgacgcagctcttgattatacagtgattggcctgagcagcgccctcatcctcgctcggaagggctac
agcgtgcacattgtggcccgcgacttgccggaggacgtctcgagccagactttcgcttcgccgtgggctgtaggtcgtctcgttcgggctctcagatgtccctgtga
gctgaccacttccatgtgtgacgcagggcgcgaactggacgccgttcatgtctctcacggacggtcctcgacaagcaaagtgggaagaattgactttgtgcgcttc
ccttctgtgtctctttttcacttcgtgctaaccaagctcgtgctacacagcaagaagtgggtcgagcttgtaccgaccggccaggtcatgtggctcaaggggacgag
gcggttcgcgcagaacgaggatggcttgctcgggcattggtacaaggacatcacgcccaacgtgggttcaccgctagttctgctgctcctcaacctcaccaggtc
actgacgaccctccttccgtcgcacgcagtaccgcccctcccttcctccgaatgtcccccaactcgatcggcgtaacctacgacaccctctccgtccacgcacc
aaagtactgccagtacctcgcgaggggattacagaagcttggcgcgacgtttgagagacggacggtcacgtcggttgagcaggcgttcgagggggtggatttgg
tggtcaacgctacgggtctcggtgcgtcgtaccttgtctctcatgcgtcaagcagcttactaacagtcctctcgcaggcgccaagtcgatcgctggcatcgacgacc
aagccgccgagccaatccgcggtcaaaccgtcctcgtcaagtccgcctgcaagcgctgcacgatggactcgtccgacccctcttctcccgcgtatatcatcccac
gaccaggtggcgaagtgatctgcggcgggacgtacggcgtgggcgattgggacttgtccgtcaaccctgagacggtccagcggatcctcaagcactgcctgcg
actcgacccgtccatctcgagcgacggcacgatcgagggtatcgaagtcctccggcacaacgttggcttgcgacccgcacgtcgaggcggcccccgcgtcga
ggcagaacggctcgtcctccctctcgaccggtccaaatcgcccctctcgcttggcaagggcacgacaagggcggcaaaggagaaggaggtgacactcgtgca
tgcgtatgggttctcgagcgccgggtaccagcagagttggggcgcggcggaggatgttgcgctgcttgtcgaggaggcgttccagcggtaccacggcgctgca
agggagtcgaagctgtag
>Seq3 [organism=Rhodotorula graminis WP1] D-amino acid oxidase gene, complete CDS

tctcgtccctcgccctcgttgactgacgcagtcacctctcgcagatcgagcacggctggcggacagccaagcgcccgggcgccgactacttcctcgcctacctgt
cgcagttctacgagatcgtcctcttcaccaccctcccgtcttatgtgcgtccctctctctctcccgtcccgccctctccggtcacaaagacgtcgtcctgactctcgc
gcactccctcgcccacagctcgccatcccgcagatcgagcagatcgacccgtacggcgcctacatcccgtggaagctgtttaaggaggcgacgcggtacaaga
acaacgcgcacatcaaggacctgtcgtacctgaaccggccgatcgagcgcaccgtcatgctcgacgtcgacccggagcggacccagctccagcccgacaact
cgatcgtgctcaagcggtgggagggcacccgcggcgacgcgaccgccaacgagctcgtcgccatgatcccgttcctcgaagctctcgccatcaagggcgtca
aggacgtgcgccccgtcatcaagcactacgagggcaagcacatcccgaccgcgtacgccgaggccgagctcaaggccaagcaggagctcctcgaccagtgg
gaggcgcagaagagcgacacgagcgtcaagggctggatcggctcgctctttggcggcctcaccaaggtgcgtccctcgctctctctctctccagccttcgtcc
cctcgagactgactctcgctctcttgtgtgcagggcactatccgcgatgccccgcccgagacggacgtcgagaaggtgcgcaagacggcgcagaagctgtacct
cgacgagcagaagtactggcgcgacaacgccgagacgatcaacgcccagatcgaggaggacaagcagcgccagctcaaggagatgtcgagctcgctcatcg
gcatgatgggcctcaagccgccggcgccgccgcagcaggcgtgagtcacgagtcgaggcgagggacgaggagcgagggagtgtgtatattcgcagaggga
gactcgcaacaagcaaatctgtacgaccgtcgccgacccattgtaccacccccctcccctcgtagtcgtctgctgcaacctgccctcctctcccatctctcggaccgt
cgtgagcggccccgagagcgcttgcgacctcgctctggctcgtgcgcttcgagttgtcgagctgcgagggtgcggcttggcgatgtcgtcggggtggaaggtgc
tgtggcggacctgcatgcgtcggagggacagggagagaaagagagtcagtgaggttgctcgggacgaggagaacggctgcttgtgctcttgaagcgggagg
ggggccaaaggatcagatctggcctaatgtgtcgccatgtcgagcgcctcgtcctcgccgatccagtcgagcgcaccgcgccgccggaacgcacagcgcgag
ggaagggttggcgacggcgagtgaggtccgtcgaagagggagggcaggacgagaggcgccacgagaactcggatatccattctgtgtgtgtcatcgtgtcga
gaggggccctcctgcttcgcgttcgagtgagagagagctcacctcgaggttcgagtgcgagtcgagcggtcggggcgagggctcagccggcggggatgccg
cgcttgtggtgcttgacggcggcgaggctcatgctcatggtgacgaggacgaagccgaggaacttgaagaaggcggtgcgcgagtactcgcgctcctcgagag
gcgagcggaacgaggcgaactggacggtggggacggggtcagcgcggggcacgcacgagggaggggaggagagggtgcgcacaatgttgcgcaggttg
ggcagagccatggcggtgttggtcgagcgagtcgagtgaccgtcctggacgagacgtcgagcgacaacgacgcttggctccaccgccgcgcccgctgcagc
actagccgctcccgctgagccagcttgctccctctcgttcgcactacaatggctatcgacaagcgcgtcgtcgtcctcggcaccggcggtgagccccagccctca
cactctccccctccccccacttccgaggcctgactcttccctctcgcccctcgcctcgcagtggtcggcctttcgtgcggcctcgtcctgtcccgacaaggctacc
gcgtccacttcatcgcgcgcgacttgcccgaggactcgacctcgcaaggcttcgcctccccctgggcggtccgtctcctcggcctcaaccctcccattcttccctgt
ttatagacccgagctgaccctctcgagtacgtgcacgcagggcgccaactggacgccgttctactcgcgggacgagggccctcgccaggccaagtgggagga
ggccacctttgctcgctgggtctcgctcgtcccgtccggcctcgccatgtggctcaacgacacgcgccgctacgccgacaccgacgccggcctcctcggtcact
ggtaccgcgacacggtccgcaactaccgcgagctcccgccgagcgagctgcccaaaggcgtcgcggccggcgccgcgtacgacaccctgtcggtcaacgc
gccgctgtactgccaggcgctcgcgagggagctgcagacgctcggcgcgaccttcgagcgcaggtcggtctcgtcgatcgagcaggtgttcgagggccagga
cgacattgcgctcgtcgtcaacgccaccgggctcggtgcgtcctctttcgccgacctgtccttctcctctcctcgagcagagtcgctcaccgtgtcttcgagtgtttgc
aggcgccaagtcgatcgctggcatcgaggactcggcgtgccacccagtgcgaggccagacggtgctcgtcaagtccggctgcaagcgctgcaccatggactc
gtcgagtgcgtcccctcgcgtcctctttcggtcttggttccggacgagactgacgctgcgctcgtacagaccctgaggcgcccgcctacatcattccgaggcctgg
cggcgaggtcatctgcggcggcacctatttggtccgtcccactctctctctcgctctctcccgacgctcgtgcgagacatgccctgaccttgcttccgcccatcctca
cgcaggtcgacgactgggacctctcgccctcggcctcgaccgcgcagcgcattctcacccagtgcctcgcgctcgacccgtccatctcgaccgacggcacgct
cgacggaatccacatcctgcggcacaacgtcgggctgcgccctgcgcgcaccggcggaccgcgcgtcgaggtcggcaagctcacgctgccactcgtgcgctc
gaccgagccgggcacggcgctcgcgctcggaacggctcgccctgctcctgcgggcgcgtcgagcgaagcggtgggtgcgccgagcgaggcggtcaagcg
cgaggtgacgctcgtgcacgcgtacgggttctcgagcgccggctaccagcagagctggggcgtcgcgcaggacgtgctcgggctcgtcgagggcgagatcg
ggccgcctcgagcctggtggacgcagaggggcaagctgtag
>Seq4 [organism=Rhodosporidium toruloides MTCC 457] D-amino acid oxidase gene, complete CDS
aagacatcccgctcttcctcgcccaaatagccgccctcctgcaaggccaacagtggggcatcggtacgcagcgcctgttcgtgacttgccctgtgccattcgctga
ctaagtattcgcaggtcacgacgctgccgccatccttcgtgggctattgcagaggaagctctgattctcttcgcctctcctgggttgtccgatagtcacgctcgccagt
ctgttgttggtactcctagctttgccctgcatattgaacagtctacaactcgtccttcttgcgcggcgggaagcgcctgagcacctcattctgcacgtcctgcggaaga
tactcccctcccgggcccgcccacatcaccggcaaatctgtccgcttctcgtacacggctggagaggcaggctgcgaggcgagagagcgctggaccgcctcga
ctttttccatcacgcgaaggaggttgccgccgatcaagtccgctaggtcgtcttccgtccagcctcgtcgcaacagctctgcagtctgcgcagacaagcttgtcagc
attccatcgaggcgggagagcgaaacgacgcgcaccaagttcggaaacttcgacgcatcctcaagcccctcgacgctcgagcgcatgccgtcaaagtcggacc
cgagaccgacatgcttcttgccgcagacggtggcgatatactcgatgtgatccgcgacgcggatgtgcgttgcgttcgtagggtcgatgaaggaggggtagaag
acgaccatcctgcgtgcggcgggtcaggaagtgttcactcatgaaagaagcaaggcgaacgtacacgatgccgtggttttggctctcgcctggcccgataagctt
gaggacctcgtccgggacgttgcgaggatggtcgtggatcgctctcgcgccggagtgcgagaagatgacgggtgcggcactgagctcgagggcctgctagcc
aagtcgtcagcgaagtctcggaagtgttgcagcgaagtgcgcacatcgagcatggtctgatcactggtgtgcgagaggtcgaccatcatgccgagcctgttcagc

tccggtacgagttcgcggccaaatgcagttaggccattcccgtcatgcacaggctcgataggagagccatcgccggcggaagaggcaaaggctgcgggagca
ttgggtcagcaagctaaccgaagtgaaggactggatccgctcacaggtgtggcaggtgtgcgtgagcgtgaggtatcgcacgccaagttgctggaagagtcgca
ggatggcgagcgagttcatcaggtgatgggagctgttcgcaagagccagtcagcgggacgaagtcgggatagactgttcgtgcacacccttccaagccaataag
cgacgcaattttgccctccgcaaacgcactccgcacctcgtccgctgtccgagcgagtgccatctcttcaggataatgctcgaccatccgatgaatgaggtcgaca
ctctcgagcgcccactcgaccgcattcgtcggctcgaggaagtcctctcccgtcggttgcgcatcgcagggagcatacgcgacatggaacaagcctcctacgcc
tcctcgacgcagtttcgggaggtcgacatggcctgcgaggccggttgcgagttcagggaggacgtcgagcggtctccgggagaggtggcgggcgacgtagg
gaaggtcgacgtgtccgtcgatgagtgggtggcgtttcaggatcccgcgggcttcagcgagcaaggagtcgtcgggtaaagtgaaggtcgactggtaagcgttt
gcaaggccgcagagcagcgctgcgacgacgggaagcttcggcacgagcatgagtgtgaatgatggtccaaggaggacagcgcagagtcaacaggagggca
catggaggcagagcgtggggcggaggaggcagatggggagtcgcgctgggggacgaggggggtgtcgctcgactaacagctctctatcgctcttgctgctgctt
gtactactcgaacgacgccatgcactctcagaagcgcgtcgttgtcctcggatcaggcggtgcgtcttttccctctcctccccacacccgacagtcctcgacgaggt
gtaggacggcgagcaaagctgccgagggcgatctgggctgactgagcgctcgagtgtacagttatcggtctgagcagcgccctcatcctcgctcggaagggct
acagcgtgcatattctcgcgcgcgacttgccggaggacgtctcgagccagactttcgcttcaccatgggctgtgcgtcgtctcactgtagttggaggatgtcagcg
agagctgagcaatctcgtcatccccgcagggcgcgaattggacgcctttcatgacgcttacagacggtcctcgacaagcaaaatgggaagaatcgactttgtgcg
tctccttctacctcattcttggcctcgagctgacgagtgtatgatacacagcaagaagtgggtcgagttggtcccgacgggccatgccatgtggctcaaggggacg
aggcggttcgcgcagaacgaagacggcttgctcgggcactggtacaaggacatcacgccaaatgtgcgcccacattcactcttcccttcgcatgtctccgtttact
gacccgccctctttcgccgtgcgcagtaccgcccccctcccatcttccgaatgtccacctggcgctatcggcgtaacctacgacaccctctccgtccacgcaccaaa
gtactgccagtaccttgcaagagagctgcagaagctcggcgcgacgtttgagagacggaccgttacgtcgcttgagcaggcgttcgacggtgcggatttggtggt
caacgctacgggacttggtatgtcccgaactgcccctctctacctgcaattttgctgattgatatgctcgcaggcgccaagtcgattgcgggcatcgacgaccaagc
cgccgagccaatccgcggccaaaccgtcctcgtcaagtccccatgcaagcgatgcacgatggactcgtccgaccccgcttctcccgcctacatcattccccgacc
aggtggcgaagtcatctgcggcgggacgtacggcgtgggagactgggacttgtctgtcaacccagagacggtccagcggatcctcaagcactgcttgcgcctc
gacccgaccatctcgagcgacggaacgatcgaaggcatcgaggtcctccgccacaacgtcggcttgcgacctgcacgacgaggcggaccccgcgtcgaggc
agaacggatcgtcctgcctctcgaccggacaaagtcgcccctctcgctcggcaggggcagcgcacgagcggcgaaggagaaggaggtcacgcttgtgcatgc
gtatggcttctcgagtgcgggataccagcagagttggggcgcggcggaggatgtcgcgcagctcgtcgacgaggcgttccagcggtaccacggcgcggcgc
gggagtcgaagttgtag
>Seq5 [organism=Rhodosporidium toruloides NP11] D-amino acid oxidase gene, complete CDS
aagacatcccgctcttcctcgcccaaatagccgccctcctgcaaggccaacagtggggcatcggtacgcagcgcctgttcgtgacttgccctgtgccattcgctga
ctaagtattcgcaggtcacgacgctgccgccatccttcgtgggctattgcagaggaagctctgattctcttcgcctctcctgggttgtccgatagtcacgctcgccagt
ctgttgttggtactcctagctttgccctgcatattgaacagtctacaactcgtccttcttgcgcggcgggaagcgcctgagcacctcattctgcacgtcctgcggaaga
tactcccctcccgggcccgcccacatcaccggcaaatctgtccgcttctcgtacacggctggagaggcaggctgcgaggcgagagagcgctggaccgcctcga
ctttttccatcacgcgaaggaggttgccgccgatcaagtccgctaggtcgtcttccgtccagcctcgtcgcaacagctctgcagtctgcgcagacaagcttgtcagc
attccatcgaggcgggagagcgaaacgacgcgcaccaagttcggaaacttcgacgcatcctcaagcccctcgacgctcgagcgcatgccgtcaaagtcggacc
cgagaccgacatgcttcttgccgcagacggtggcgatatactcgatgtgatccgcgacgcggatgtgcgttgcgttcgtagggtcgatgaaggaggggtagaag
acgaccatcctgcgtgcggcgggtcaggaagtgttcactcatgaaagaagcaaggcgaacgtacacgatgccgtggttttggctctcgcctggcccgataagctt
gaggacctcgtccgggacgttgcgaggatggtcgtggatcgctctcgcgccggagtgcgagaagatgacgggtgcggcactgagctcgagggcctgctagcc
aagtcgtcagcgaagtctcggaagtgttgcagcgaagtgcgcacatcgagcatggtctgatcactggtgtgcgagaggtcgaccatcatgccgagcctgttcagc
tccggtacgagttcgcggccaaatgcagttaggccattcccgtcatgcacaggctcgataggagagccatcgccggcggaagaggcaaaggctgcgggagca
ttgggtcagcaagctaaccgaagtgaaggactggatccgctcacaggtgtggcaggtgtgcgtgagcgtgaggtatcgcacgccaagttgctggaagagtcgca
ggatggcgagcgagttcatcaggtgatgggagctgttcgcaagagccagtcagcgggacgaagtcgggatagactgttcgtgcacacccttccaagccaataag
cgacgcaattttgccctccgcaaacgcactccgcacctcgtccgctgtccgagcgagtgccatctcttcaggataatgctcgaccatccgatgaatgaggtcgaca
ctctcgagcgcccactcgaccgcattcgtcggctcgaggaagtcctctcccgtcggttgcgcatcgcagggagcatacgcgacatggaacaagcctcctacgcc
tcctcgacgcagtttcgggaggtcgacatggcctgcgaggccggttgcgagttcagggaggacgtcgagcggtctccgggagaggtggcgggcgacgtagg
gaaggtcgacgtgtccgtcgatgagtgggtggcgtttcaggatcccgcgggcttcagcgagcaaggagtcgtcgggtaaagtgaaggtcgactggtaagcgttt
gcaaggccgcagagcagcgctgcgacgacgggaagcttcggcacgagcatgagtgtgaatgatggtccaaggaggacagcgcagagtcaacaggagggca
catggaggcagagcgtggggcggaggaggcagatggggagtcgcgctgggggacgaggggggtgtcgctcgactaacagctctctatcgctcttgctgctgctt
gtactactcgaacgacgccatgcactctcagaagcgcgtcgttgtcctcggatcaggcggtgcgtcttttccctctcctccccacacccgacagtcctcgacgaggt
gtaggacggcgagcaaagctgccgagggcgatctgggctgactgagcgctcgagtgtacagttatcggtctgagcagcgccctcatcctcgctcggaagggct

acagcgtgcatattctcgcgcgcgacttgccggaggacgtctcgagccagactttcgcttcaccatgggctgtgcgtcgtctcactgtagttggaggatgtcagcg
agagctgagcaatctcgtcatccccgcagggcgcgaattggacgcctttcatgacgcttacagacggtcctcgacaagcaaaatgggaagaatcgactttgtgcg
tctccttctacctcattcttggcctcgagctgacgagtgtatgatacacagcaagaagtgggtcgagttggtcccgacgggccatgccatgtggctcaaggggacg
aggcggttcgcgcagaacgaagacggcttgctcgggcactggtacaaggacatcacgccaaatgtgcgcccacattcactcttcccttcgcatgtctccgtttact
gacccgccctctttcgccgtgcgcagtaccgccccctcccatcttccgaatgtccacctggcgctatcggcgtaacctacgacaccctctccgtccacgcaccaaa
gtactgccagtaccttgcaagagagctgcagaagctcggcgcgacgtttgagagacggaccgttacgtcgcttgagcaggcgttcgacggtgcggatttggtggt
caacgctacgggacttggtatgtcccgaactgcccctctctacctgcaattttgctgattgatatgctcgcaggcgccaagtcgattgcgggcatcgacgaccaagc
cgccgagccaatccgcggccaaaccgtcctcgtcaagtccccatgcaagcgatgcacgatggactcgtccgaccccgcttctcccgcctacatcattccccgacc
aggtggcgaagtcatctgcggcgggacgtacggcgtgggagactgggacttgtctgtcaacccagagacggtccagcggatcctcaagcactgcttgcgcctc
gacccgaccatctcgagcgacggaacgatcgaaggcatcgaggtcctccgccacaacgtcggcttgcgacctgcacgacgaggcggaccccgcgtcgaggc
agaacggatcgtcctgcctctcgaccggacaaagtcgcccctctcgctcggcaggggcagcgcacgagcggcgaaggagaaggaggtcacgcttgtgcatgc
gtatggcttctcgagtgcgggataccagcagagttggggcgcggcggaggatgtcgcgcagctcgtcgacgaggcgttccagcggtaccacggcgcggcgc
gggagtcgaagttgtag
>Seq6 [organism=Sporobolomyces roseus] D-amino acid oxidase gene, complete CDS
ttacatcccttggaaactgttcaaagaagcgactcggtacaagaacaagcaactgatcaaagacttgtcatacctgaaccgacccctcgaacgtaccatcatcctcg
acactgacccttcgcatttccaactccaacccgagaacggaatcacgttgaaaccgtggttgggaacgaaaggggacgacgctacgacgagagagttgatcgcc
atgattcccttttttggaagcgttggcggtcaaaggcgtcaaggatgtccgacccgtgatcaagttttacgagggtaaacacattcctacggcgtactatgaagcaga
gttgagggcaaagagagaattggaagacaagtggacaagggaaaagacggacaatagtttcaaaggttggatttcttccctctttggtggtttgacaaaggtaatac
tttttctctttctctgatgtctgttttccttttttggaggaggagaagtgctgattccaccctctctctctctctctctcgaacacgcctcctgccgttgtagactactatg
cgagattctgctccagagacggacgttgaaaaagttcgcaagtcggcacaaaagctctacctcgacgaacaaaagtactggaaagacaacgaagaggtcatcaa
caagcaaatcgaagaagacaagcaacgtcaaatgaacgagtgagttctttctctttctctctcggaatgaggggcatctcttgacaggattgactctgatttctctcgg
ttgttgtgttataaacaaaaacaggatgaggagttctttgttgggaatgatcggattagttcctcctactcctccttcggccgctcctactgcataaattttcgttctctccc
cccctcgctctgtttgtatcataaatgtcccttttgcccctccctctcctacctactgcaaacctcggaggaaagggattcgattacctctttttgcagattatctgaacttg
aaaaagccccccctctcctaccctagctatcaagtgattgaactctcgaggcgaaggaaagagacagagatagagagcgagagcgaggtgtgtcggaggagcg
cttggattgactagtaggaaggtgtcgatttgatcgcctcgtctcgttggcggagaaaggaggggggtgccgtctgcgcgcgtggggcacggatgaacgaacga
aactgttggacccgcccaagccggaaacaaccaagttttcggatcgccccgaagatcctgcctgtcgcccgaacgaagaagggaacgagtttttttagtcagctct
atttggatcgtctctgggaggtcgcgaacgtggttccgtagaaaagtaggttcagatctggccttgtttctttcttcgccatgagagaggaacaatgagaaacctcgc
ccttgtacatctccaacctggtccccactcatcctttctccgattcctctagagacgcacactccgaggagagggttccttgagagagaggaagagagcgaggcgg
ttgaatgaaggacgacaaaagacgagatctcagattgttcgagagagtgtgtgtgtgaatgtccgatatccattctgttgctatattgctcatcctgtactttgggtatac
gctgccgacgctagcgagctagagagagcgagagagagagaatgctcaccttttggttcatcaacgcaggaggatcgatagatggttgcgtttgttctttgtcgcg
atcaaactcatgctcatcgtgacgaggacccagccgaggaacttgaagaaggccgtacgagagtactcgcgttcttcgagcggagatcgaaacgatgcaaactg
atcgatccacgggaacccggcgcgtcagtctcgatccaacagttggaagagtaccaccagactcacaatgttgcggaggttgggtagagccatgtttcagagtga
ggaatccgaggacaaacgatcagcttgactcttgacacttgactcttcactcttgtttcccttcgatcatgccgacgcgtgagcctcagagagtagtcgtactaggcg
caggaggtatgtagcaacggatttccacttcggctactcccgaggttttcctcccccacttgttttagccgccaaccgacctctccttatctcgcgctgcactctgctga
tgcactcgagggaactcggacagtcatcggattaacttgcgccctttcactcgtcgaacaaggctacacggttcacgttgtcgcaagagaccttccggaagacacc
gactctaccgcgttcgcttctccctgggccggcgccaacgtatgtccattcaagagtctcgaggaaggtccgagagaagcgagatgggaaaccgtcactttgtca
gttgttacttgcctctcctctcccccgagtcgacccgaactctctcattcgactttttgcactttcccacttttctagcaaaaaactttcaaccatgatcccatctggcttgg
ccatgactctcaaaggtactcggaggttcgcacacgaagaatcccagttgttgggccactggtacaaagacgtcgttccgaacgtgagcttttcttccttcttttcttcc
ccccgacctcgtaactccgtctcctctctgagctcgagtctcgctcgaagcaaattacttcgtgtgttctgatctccgctttgtgtgagttgattagtaccgacacttgaa
tcgcgaagaatgtccacccaatgcgatcggagtcgaattcgataccatctcggtcaacgcaccgaaatattgtcactacctcgctcacgagttgaggtcaaaggga
gtcacgatcgagaggaggtacgtcaagtctattgaagaggtttttcgagaaacgtttggagcgagaaacgatttcgtcgtcaatgcgactggattaggtactattttcc
ctttcccgactctctctctctctctctgtctgtctttcctcataccgaactcgttgtattgactgatttgctcgtggttataggagcaaaaagtatcgcaggagtagaaga
tcaagaagtgagaccgattcgaggtcaaaccgtcttgatcaaatcggattgcgttcggtgtacgatggattcgagtggttgagttctttgtctttctccctataccccttt
ccccgaaccttgagtgttgcattcaatcgctaaataacgttcttgtgtgcgggttttcttctttcttcagatccaacctcttccgcttacatcatccctcgacctggaggtg
aagtcatttgcggtggctcttacggtgtacgtctgttacgttttccctctatctctcccgaggaatcaaagtcccaaactcatttttttttctttctctttttgggtctcttctgttc
gtcgacaggtggacgactgggacttgtcggtttctcccgatcatgcgaaacgtatactctctcactgtctacgactagacccttcgatctctcgagatggtaccctcg

aaggcatcgaaatcttgaggcacaacgtcgggctgagaccttctaggagttctggaccgagagtcgaaaaggaaatcatcgagctgaaccacgaaaagagtcc
gattcggatcgggtctcgtctcgacgatgcgaccaaggggaagagtgcgaagacacgtggagtggtggtacacgcttacggagtgggaccggcgggatacca
gcagagttggggaatcgcacaagacgtcgtcaagttgatacaagaaggagaagcacgagaatccaagttgtag
>Seq7 [organism=Puccinia triticina] D-amino acid oxidase gene, complete CDS
gttggtcgtcgcactcttctcgccgctcgagctgatccggacgcggctccaatcaacaccggtagcccgcccgcagctgctccggcttgcgccgttcgacccgaa
cggccggggtggcgtggcgagtccgcgcccgatactcggcacgctactaagctcagtgcgctcgacgggtctccgaactctctacacaggacttccagcgacg
ctgtggcgggatgtgccttttagcgccctttactggtcctcatacgaggcctcccgacggatcctcagcggtggactcggcttcggcgaggctgatagtcgggcta
gcgtccaccagcttgccctccagagcttcttcgccggctccctcagcggcgctgtcgccgctactctcactaaccccttcgacgtcgtcaaacccgcagacagg
cttccccttactcgccttcctcatcctcccacactccaaggccccctgggactatccgtattatccttcagatcgctcgctctgaaggcattagaaatggactcatgag
aggcctctcgccccggctggctaaagttatccccggctgtggaatcatgatcgtacgtcctcacttctaactattactctactttttctgatcttcatggtcttttgcctcac
tcgacaggcctcgtatgaaggactcgctcaagtactctcaaagtactagcttcacacacgagttttggacataactcgcttctctaatgacctttcgcgattacctttcc
atcctgctcatgcagtatcacatggacattttcttttgctgtacttgatgtaaccttcatcttttttggttagggtttgtttcgttctctttctcacgcaggattgactttttcatg
aatagactcatcatcatcatcgtaattttccatcgtcgatacgttcaattgctcaaccaactaatcgagcagtccataaacaaaaaagtacatatctcttgtagacttctct
caatttgtttgcccctcctctgtctttccaaagaaaaaagaaaacagtatttacgcaaaaagaataaatgaacgctggtcaggttactgaccagcttaatttttacttcctt
ggtttgtctaggatatatagtggtttgataagaaactggctttcgctttcaatagcatcacatggcgtatggtacaaattaccacaaccattaaatctggtgcctgtctac
cgtagtaccacgtcagaaggaaaaggactgagtaagtttgattatccactggctctgaaggctctgatccggacaccgcagatccgtcgaaatgcggcaaccctc
cagacccgatccgtattcgggacggagctgatgaaatctaggatccggccgtaaaagtgaggctttgggtaaaccccggaaagccgcctcagatcggggattcc
ttcggggaattcctgggaatctcgcagcacgagcttattgttgtacttttgtttttgcaataaatatcaaaaatagccacattgaaatccagttgaatccgaccggattct
ggatacgaccagggagagggtgcgggataggtgatatattccagctccgaggcaccggattggaccctcaaaatttattctggctcgaaggagacggcgatgga
agcttcactgtgtgatggctaccattcagttctccaccaggaagtgaggtttggcaacagtttcagccgatagcatatcatgccctcatctgtatatcctcttcagcatc
ggtggtctttaaccctggtggaccaagcatggatggatgcactccatggtttgatttgaaagacatgtttgtaatataccttgtctccgaggaggtttccccgctcctgc
tgtgcaatcaggtgcctttatgtattttgaggaccatcattcctggtctcaagccattcagttaacatctcatcaagtgcccacccccaaggacatattatcgccccacg
aatctggtccttcaatcctcgagttgctagtgatctctggtcatgtccgccccaccgagccaagagccgccgtgcattgtcaacgtcatcggatccggtgtactcggt
ctcacctgtgcactcgagctcgccaaacagccgggctcaccatatcgtgtcaatgtggtcaccgccgaaagcgcgctgactgcatggtctcaagacccgtcgacg
aagctcccggtggaaccagatttgcttccccttgggcggtacgtgtttcaaatgagacacatttctttcattcattttcattcatttggcagagaaaaaaaaattatgagc
cggaggaagaagtgacttgacctcttttctgattgcgcatgtagggtgctttctggcaccccttcactcctcaaccagagaccgagctgcagaaaagggtagccgg
atgggagacgaagtcgtttaagcatctttgggagattgctgagcaagatccttcggttgtcatggttagcggctcagacggctcaagccctccccatccacaccctc
caagatctgctgaaaaccttcgtttctcgggggctattgtagaaagctgacttcaagaaatattatgaccatcggttgactgaattaccgtggtatatggacctatgtcc
ggaggtgaattatccgttggtttatatctggacactccaatctttccaattgagtaaactgacttgagagcgcacgtgattgaaaggctcgaaaaatgaacgacgaag
aattagcgaaggcgccagaaggaaaagtagatggaataatctgtaaatccgtttccttgaacccaatccgttacctggattacctcagaaagcaactggtcaaatac
gaagtccaaatcattcaccatcgtcttgaaaccgtctcggaggcttttctgggggatcccaagtttggcatcccggcggcgaaaatagtagtaaatgcgtcaggtct
cggcgcgggcagcttgtgtggtgtgtcagacgaattgatggagccgattcgtggtcaaacgatcctcatcaggccccctcaaccgatccaactaatcacacggga
tgacgataaatgtatctacatctgctcgcgcccacctaccatacccggcgaaggcgaagaggtcatcttgggtggctcatatgaacctggcaactcctctttatccat
cgacaacggaatcgccgataggatcttgaccgaagcgctcaagctccggcccgatcttagccgtgacggcacgtccaacggcatcgaaatattggatcatattgc
ggctttgaggcccagtcgcaaggatggtcctcgggttgaaagggaaaatttcacgatttctaagaaaggagaaactcaggatgaaactgggctcttggtccattgtt
atggaattgggtaaatttcttccctcgttgtcctaacctggtaattccttagcaaaccggtggctcaaatttacttccgtattacactttttgcattgatcagaggaggagg
ctttcaggtgcgtgaatgaaggcatttgatttctgcctgagcgtaaagtgaagagcatctcatcggtgatgatttttttcttgcttcccgtgcacgtgaaaggcgagctac
ggggcagcggcagaagttagaagcttgatagagactcagaaaaacatctcttga
>Seq8 [organism=Puccinia graminis] D-amino acid oxidase gene, complete CDS
gtatcttctgtatcactttccattatttgtccattgtggtgtgttttgatttgtattccaggattacagtttctatagttgtgttctctgtctatacaatgaatgtcctcccaggtat
aatcttcaattgagctaaatctattagtttaagttgtacttttaccgttttgagccatgtaatgtcttgttttggctgccagaaagtagcagacaattcagtgcagttgagcta
aaagttttgagcaggatcagaagcacattcttggagaaggcagatctgtgcaactgatgctttcttctttttggaatgagtgcttgatggggtggttaaggtcagacatt
gtattgtcactggggttctagttttcttgggtggcataactacagaggctcaagtgtgaggcttggaaaatgaaagagatgacattgagtggttgggtgagatgggtt
ggtctgattatatcctgttgacccgctcaatgttgcaaggtgagctgactgtggggaggggggcttctagcttatcacaatcccctcgttccagagggccgggggaa
ccccagcatgccgggggcaccttgcaaagcgagcctctgaaagaggagcttgggagggaagtcggaaatttggtctggaagcttgggctttttgtggcttttctct
cctctttcacatttaaaacctcattactactaggccatcccaccaaatggttagcggaaaatttccccaagaagctgagcacctcaccttgttgatcacttttttttgtcac

atttttattaaacaataatccatctgatggatgttaagtggggcaccagatcccagctcaaaagcagctaatcacatataatgtggtacaacacatagtattagccttac
agttagcataggtatgtgtcatacctagaaggtatgtagccataaatgtgtacagaaaaagtattgggcaagatcaaggaatccgtttggtgaaactggaatactgtc
atcaaactcccaagaaaatgtccatttttcaagggggggtggggcccaataaccggcctgtgtacctaaagtgttacacagaaagatagagaagatctgggggatttt
gtctgtctaaaaggcagagattcattccagtggaaagggagggaaatggttggaggaaactttttccctatcaacagctcccagcttgcacacaaaaacacagacc
ttcagggcaagtccctcctttggaggggtgcccggtgctttgcaccagcctgatctcaggccctccgattgaggggcttgtggttaggaatcaaaaaacagggattt
gtatacaacagcatttgatccagtttgatgtgaatttcagacctgattcctgttcctggagggttttcctagacaggagcacagtttcacaagtggtctttctctttactgg
gttttgacctgcacaggctcttgaggcagttttgaaacccccctattgggttaggtggtgaacaggaatgatcttttcattactttcaaaaaatcatagaaggggaaccaa
gctggcagatgaggagatgagaccacctgccaaatgaaagcttggtcctaaaagttcaatctggctctgagccaggctcgaggcctgtagagcattataaggcta
aggagccagggccttaacaaattaatccattcacttgccagccatttgcatttgatgtcccaaccaaaaaatttggcttggggagttgtgatatgtaagtgaggaagg
acttggttaagtcaggctcggctcgagaaacagccgactgcgtaactccattggcatttggatgagttgatagtggaatggccagtgctcatgggttccagtgaaat
cgattgatctctccaaagaaatcttaaatattagcctgaagaaacataagcatcactgctcacttaaaatcatccttaacaagtcattcatatcgaattcctaccccccctg
aatctgatcggttgagccgccagtatctcttgtcatgtccaatgtcaacgtcatcggatccggtgttcttggcctcacctgtgctctcgagctcgccaaacagcccgg
atcaccataccgtgtcactgtggtgaccgccgaaaacgctatggctagatggtctcaagacccgtccacgaagcttccagtggagcccgattttgcttctccttggg
cggtatgtcataaatagactttcggttcatcagtttgaatggaaaaaatgaaaagtgatgaacagggtaaagaaagaaccattttctgatctcgggatgttctctaaattt
agggcgctttcttccacccccttcactccacaacccgagaccgagctacagaaaagggtggcctcatgggagaggaagtcgtttaagcctctttgggagatcgctg
agcaagatccttcggtcgtcatggtcagcggtctcagacggctaaaactctctatcttcatcctccatcaacacgtgctgaggttcttcatctctcggtggctactagaa
aaccgacttcaaaaagtattacaaccatccgttgtccgaattaccgtggtatatggatctgttcccagaggtgaagaaatcattggatgcgctagattctctctctacgc
aagtgaactgacttgaggctaaacattttcactggaaggctcgaaaaatgaacgccgaagaaatagcgaatggacccgaaggaaaagtcgacggcctgatctgc
aaatcggtgaccctgaacccgatccggtacctagattaccttcgacaacaactagccaaatatgacgtccgcatcattcaccatcgtctcgagaccgtcactgaggc
ttttgagggccatcccgagtgcggtctaccggcggcaaagatagtgatcaatgcgtgtggtctaggcgcgggcaagatagggggcgtgtcggacggaatgatg
gagccgattcgcggccagaccatgctcatccgcccccctcaaccgctccaactgatcacacgggacgacgagaaatgtatctacatctgttcccgtccacccacg
atcccgggagaaggcgaagaggtcgtcctgggaggctcttatcaacctggcgactcctcattggagatcgatgaccaaatctctcataggatcttgtccgaggcac
tcaaaatcaggcccgatctcagccatgacggcacggttgaaggcgtccaaatagtggaacatgtcgcggctctgagacctcataataagaatggccctcgtgtcg
aacgtgaagatttccacattaaatctactagtcaaggagagactcagaattcaactgggatattggtccattgttatggaatcgggttagtcttctatcgtctcatccttcc
tcttcaatcaaattgatgaattaaaattgacctctttcctgctcacaatctttgtatcgagcaggggaggaggattccaagtgagtgaatcaaaaaaagaccgtgttcct
tcttgtgacctctgtttgaacttgggattagctcttctttgctgacatattcctggttattttgtttgtttgaaaggcaagctatggggttgcagaagaagtgagaaatctagt
agaacaaggccggtcttga
>Seq9 [organism=Melampsora laricis-populina] D-amino acid oxidase gene, complete CDS
ttcttgatcactcagtcacaaacgctctaaaactaagttttaaggccttttaaaaaaagggccaacgtactccgacgctcatacctcctttttttctgcacccaacggttc
ttctttagtcctctgccctgtttctacttgccttggtgaagttcaacggcttgttttagcagtcaattatggagtttttcgcgaaagaatccatagctaatgagaaaaaactc
tcacatccaggtacaccaaaaaacaaaacaaaaatggaagatgatctttttcatcattcggacctgacagtcataagaaatttaagcaaaattgagtgattgcaaaac
tgtgaaccacctctagaggtggttcgcgaacttataattatacttaaaatttaaagcacccgaacaaaacataaaatttaaaaaggggtgtaaaagctcttcttgaacc
accctattggtaagtcctgttcctttgctgtattgcctgctacttggtttacaagccagctcattcagttctatgcctttgtggccttgtgtccaataactgtggatactggtg
ttcttagacattttcaaatgtgtatctggattaggatccgctggactgtatttaatttgggcatatcattatgacaccctgattatcactgaaaatggaaagggcagtagga
gttggtcttagtacttcaaatagattgctagatcaattggctgctaatagtaggccaagcaacttggtttcatggtgtgaaaggctgttgttacagacaattgcttgggct
gggctaattatcttggttaccaccactgcaccccattcctcttgagtaactgagccttcagtgaagataattactgtctttcaatactttcttcatcatcaatttctacaggg
atgtgctttgatgctgcttctttgttgtgctctgttgaatatttcacctatgcactttgcccatggtgctattaggtaggcacattttgttttggttccttctgatgcaagtgactt
gagctggttgtagttggctgctttatgaatagtccctgggtgatgtttgggctggtgatgtagttcatttttgacaaaaatacctgttgggtgggtatctgaggatgttaat
cttttcaggatcaagttgtgtgtgatgtctccgctttgataaccatgtgaatggtgtgagatatgtgtagtggctttcatttgtttgaagtgcatttgaaagctctaacaaat
aattcattttctgcgttttgtctcttttctgatagtctgtttactttcatgttgttcagttatgtgtctgggggtgggcatctggctgtagactaccccccaaaagtaggctctcct
ccagtagggctctggggaaggtgggagggcatataaaaagttgaggatttgtgttccaagagcacccactatggttgtgttaacaacatgatttagggtcagtctcct
gctaaaatacagacaggtccactctagacctgacttagacctgcggtccatacaactttgtcaaatcttgcaccctgcgtgttcaaatctacaaccacctacaacttga
tttatagtaggaactaggtaaagcagatgacccatagtggatttcatgtggttaccaacttaggtaaaacatgcatgttcaaattttgcgttgaggaacgcaaaatttac
gatcaaacactcttaccatagtggattctctaagagctattgagtgtgtgctggaacttgtgatacgcggtatacgaaaagcgcgcattgagatttccaaccaatatct
gagcggcccggtactcgggtctgcgacttttcggctcacgggagttatccgcgctcccaagctcaggggtccggggaacattcggaagtcgtcgtgcttttggac
atccgtggcatacagggcgacataccattacagcctgtcggcacttgacactacttgacttcacttgactctttctagtcgaatcgctcgaaagcgtgatagtatcaat

gagcttcaatccagagcataagagcgagcacttgaatatcatcggttcaggagtcctcggtctcacttgcgctctagaaatagcgaaagaaaaaggacgttacact
ataaccatcatcactgcggatgccgattcatccacttggtcaccatcggctccaccaaaaattgagaaagtcgccgaggactttgcttcaccttgggcagttgagtaa
acctcatcgaaccaaaatctgtactcctgcctaactttcgttcaagttttgccttgaactttcaagggcgcatattggcaatcatttgtagcgactagagatcctaagacg
ttcaacgaaaaaagattacaagattgggagaaaacttccttcaaagaattatggaaaatctcagagacggacaaatcaattgtgatggtgaaatatttgattcttggag
ttctcagcccaagggatgtgttcgttgaaacatctcaacactattccagcaaaccgattgtcacgaatattttgatgaagaacttagtgaccatgagttaccttggtaca
ccgatttgtgtccagaggttggctaaagtctttgcgattcaattatgccaggtaaacaaaaagctaatggccactaccctcaatccccaaccacaagtttcgacgact
aacctcatcagagcttgcaagtctgccccgcccccgacaagcagggatttcattcaagacgatctcactcaatccgatgatgtatctaacttttctgcatactcaattat
ccaagatgggcgttcgaatcgttcaccatcgcttgaactcgcttgcggaagccttcgaaggcaataacactcttcaaattccgcgcgctgacatcgtcatcaatgcgt
caggtctaggggctgctaccttacttggtgtagaagacaaatctgtacatcccattcgtggccagctagttctagtcaaaccacctcagccaatatgtttctcgacaag
agattcttcaaggaagacttacatcatctctcgtccttccgttgacccggagattgacgaggaagtaatactaggagggtgctaccaggctgataattttgacctttca
gttgaccccgatctgactaatcacattctgtgtgaggctttccagacacggccagacctaagtagtgatggtaccctacagggaatacatgtattgaaagaggttgta
gcgcttagacctgctagaaaggatggagctcgacttgaggtagagaaagtcgtcatttctggggaaaacaagcatgctgtacattgctatggaattgggtacgtccc
ttgtctatttagtggtcaaggcttccatacactttctatgcatcccttcaagtgaattactgctgagccatgtcttgatcgcttcttacagtggtgctgggtttcaggtaaga
gatgtccctctctttcatggcccttttgtcctccgaatttaccatatttttctgcttgtgggtgcgaaacaattgccactcatttttccttcagtcaagttatggtatggcaca
ggaagcattgggattgattaaggcacttcgagagggtcgggaaactgtttaa
>Seq10 [organism=Rhodotorula minuta] D-amino acid oxidase gene, complete CDS
ttcacattgcctcctcctgctactccaccgtgcatcgctatagcctgctcgagcacgtgaaggttatttccactctgatcaaagataggcgaagcttgttggctgcttga
tgatgatttcgtgcgagatggtgcactgccagcgttccctcttcctcctcttgcactggcactaccccttccacgtcctcttgttgctgttgttgcattactacctgtcttca
cggagggtctcgctactgtggaactcgatgcactcggatttgttgctgttgctgctgtcgacaacgagtatgggaaggtcgcagaagtgctcgttattggagatgct
gtattgctggtacttcctcctctgtttggtgaatctagcgtgtagctgctgttgtcttgattaggatgatagattgctacttcgcgtttagcgggtggcctgggtgggtgtg
tgtgtacaaggaagtcagctattatgcgtacttcttcaatgcaatgcgagctgaactgtaagtccttactgtggatctcctactgcactcttacgctttctccctctgttatg
tttgagtaccctttgcaaccgtacaaagtaattagagctgttggcagtttcagtgtggaaataggtggtacacgactcactcgcaagtcaggccttgatgcacgcatct
cgagcaaggaaaaggactctctggacttttaggcacgcatttgatcttgtgattgcggcactgtgagcctgtttttcgtatttcattgatgaatgtacgtttcgaagttga
agtgtagatttcacgtaggtatggtatggcgagcggaggatcggaggggtcagcatagatgcggtagtggatcagcaccgttcacgcagcaatgagtccgcatta
aatataagaatccacccacaagcctgagcaccacgctgtattggcctctgcagctttctcggttccttgctgttgctgttcgccgcgcctgaagaaggaccacctcca
gcggtactgctcgtggcatttgctccattgactgatccatcgtctgttccctcaatagatccctctcttgtccttgtagtgtcatcctctactatgtgctggtcctctccttgc
tgaggatgttgccctccctcattctccctggaatgctgctcttccatgctaccttccgtatcgctcttctccttcttcaaacgcagcgacgagctagctgactactactact
cttctggatcaggtctcttatctaataatgaacgctttcaaagcatgcttttatacaccccggtgactacggtgatggatgtgatatctttctcggtcatcaaagagaaga
gccgcctcagaatgactgttcgaaagagagctaactcggtcatttggagaaatgacacacattcacaaagacaccgactcttttgcctttctggccagcaataaaca
actacaggtatacatctgcattctaacgtgcattgatggagtggacagcagctcaacaatgccaatgcaggccatacgaagtccgcctaccgcagctgccaacgc
cagctttgaagatcacgagaacgttgtagtcagagctagatagggcaccgtgctcctctgaggccatgcagaccttctgaagactttagcagtcatatatacctgcct
catgctgccttaggagcccattgctgccttccgtacacaataagcggtttcctcgcctcagacctgtactttggaagacgtatgccactattccgctgggtcatccatg
tgggcgccggaaaacccacacgatgcaagcgtgcgagcagaatgctaatcaaagccagattgctatacattacgagtcaaagccgtatccgctagctgtgcgag
cgaccacgcagaaatgtcaatgctttcaagataagctatctttggcttcttagcaggccgatcgggcgtctctcaaacaagttcaaacggaccggaagcgataacct
gcttttcttcttcataaccacacattctgacaagcatgacgctaccacttctcgaagatgcacctcgagcattggacgccgtcattatcggcgcaggcgtcactggcct
cagtatcggactggagcttgtaaaaagaggctacaagattgcagtcgtagcgaaggacttgcctgaagatgatcagagtcctggctttgcaagtccatgggctgtg
aggagtcttttccttcatctatcgttccttttgacatgcctactgacgcactggctgtgacattgcaggggtgcaactggtgctctttcgcaggtatgcccctccaatcttc
cgaaagattagcttcgcggggacggcaaaaaaagatcctcgcaagatgtcataagctgaagaagcgcttcccttcccccactttccttagacaacaacaagagag
agcaagaatgggatcgtaaaacataccaagccctctttaacgtagctgaaaaccatcccaacctatgcgaggtgagctaggctttcgcttagagagagagatcacg
tggtgtacaaggacgaagtgtgaaggaaccgcttcaatatagtttatgatagctgactctcttgcttttggcttgactacagctcattcagttccactcgtatgaaagcg
acaagttacctgaaccttggttcaaagatctcgtgaaagatgtaagctacctgtttttccgctacttcctacgataaattggggaatgcaaataagaaccattccgcaa
gacagtagttgacgaagctataacctctttaccctcgcgttctcaatgtcacctttcacatttctgaccacccatcagtacaaagtcacgaaggagggcacgaaacca
ggcgaaagatgcataagctacaaatccttcatcatgcacgcgcccaattatctacgtcacctagcgcagcagcttcgctcagcgggcgttccgatccagcggaaa
cgcctgacatcgctcgaagaagcgtacaacctcccttcatacggccgcgtcaatctagtgatcaacgctacgggtctaggtgctcgttcgttactgggcaataagg
atccaagcgatatatatcccatcaggggacagactgtccttgtcaaagtaccgcagggatggaagaaaacctgcttcatgatggcgagcgaccatccggacgag
aagacgacgaaagatacggaaatcccagaaccgacttatattattccgagacctggtccagagggacatgtagtactgtgagtgacgagcttcattcttaatattgtg

cactcgaggaagtctggtcttatgctggtctcatcgtagaggaggttcattccaagtcaacaattgggaatatgcaccagactacgcattagcggaacgcattctgaa
gcgtaactacgagttgtgccctgaactggcaggtccaaatggaaaatcgtggaaagacagtaaggcttgattcctatgtgacagaattggtgttacgtactgatagc
actttactatttctacagttgaaatcgtagcccacaatgtcggattcagacctagccgacaaggaggatgcaggcttgatctcgagcctattgagctaggtaacgcta
aacaagaaggaatgcaactcgctcccaaatcgacaattaccgaacctaggactggagctatattgcatgcctatggaataggtgagccacactcatctgcatcaca
gtgtggtaaaatgctccacttaaatactgatgttgcgcttcctcttttgtgtatctctgatacaaacgatgaaataggcccggccggattccaagcttccctcggatacg
ctctagaagctggagatatggtcgactcatacttcaagaagggcgacgcgaagaagcgaagttcgaagctgtaa
>Seq11 [organism=Sporobolomyces linderae] D-amino acid oxidase gene, complete CDS
gaaacgcgccgatcgacgccgaggccgggcgaccgcgtggtgccgtcgcgagttgcgaagctccacggctcgtttgcctagcgtgtccagctctggcaagtct
ggacctgctgctgcggacctgctgcgcacgccctggctcgcgcactcgctctacgacgacgaccggacgcgggtcggttcacgtcagcttggtcgggctgcgg
acggagggtcttgagggcgtcgggagcggacgtcggacgcgatgccgggcggaccgggcgctggcttacaagccgcgccgccaaagccgcgcatccagct
gcccagcatccaggcgatctttgggctgtcacgcaatggtccgtgaacattgcacgaggcctggctgaccgcgcagactcgctggagcagctgagtccgccgc
atacggcaccgcgacccccgtcgcccgcgccgtcgaacgcgtcgtcccagcgttcgcgggcatcccgcgcatcggccgatctgcggaccttctcgctcagccg
gcatcaatcgtacgacgagtcggccgcggcgtcgcggcaggtgccttttcccgcgggcctcgtcggcgcggcgtccccgcggcccgcagcgccgccgcggt
cgagctcactgcggagctcgagcagtcgcgaggagccggacgcgtggaggcgaccgcggacgcccgagctgttccgagcggcggtgcgtctagctcatctct
gctcaactgacgccgcagctggacgacggcgagagctcggatgggcattcgtcgatggaccacagctcggcgcacggcaccgcgtcgccatttggccagacg
gcgccgatcgcatcgacatcgacgcatcgcgcgacgtcgtacgcctcgtccgtccgatcggccgacacgtacttctcgcggcacagccaggcgtccacggcgg
cgtcgtcgatgcattcggtgcagagcggcaaagcgcacgacgacttgcccttgcgcccaccgttcggccctccagcgcatccgtcgagccatcaggccgacctc
gaccgggcgcgcttcgatctcagatcagccgggccgccgacgatggagtaccggagtcggtcggtcccgcaggcgccggggtattggccgcagccgccgtc
gttgccgccgagccatccggcgcaggcgatgatggcgccgccgctgatcgcgccgccgccgctcgggcgacgagcgaccgtgccatcgccgtcgcattcgct
cggcgggccgtggacgcatcaggcgccgccatcggcctacacgcatccgcaggccgcggccttctactcgccgccattgccgacgtcgccctactcgcccgc
ggcctcgcccatgtcgccgtccgattcgaccacgtcggcgggcgacagcgacctgccgcgctacgcgtgtacgcagtgtccgaagcgattctcgtgcgctcgtc
gttcttcttcgcaacgctgacctcgcagtcgcccctcttcgctgcgcattcaccagtacagccactcgggcgagcggtgcgtcggcgatgcatcgggcgatctgac
catctaggcccttcgtctgcgaagagtgcggtcgcggcttcagcgtgcaggtgcgtttgcgctgcagtgccttgatctgaccggctctcagagcaatctgcgtcgg
cacggccgcgtccatcagcccaagtcgggcgtgccgccttcgcccgctgcgtccaccgtcacgatcgtcgaggaggacgactacgacgaggatgaggacgc
gatgcaggtgggatgaggcggcgacgcggcgctgtcaatggcgctgtcggagcgaagttcacgttcgatcgtccattcgacgatacccacgccaatctgtgtac
aacacatgctcctccgttaccctgtgcactatcatacaagtctttcacacttgtccacgcggcccaagcttgtctgcgcggcaacccgagcgcgcgagcgcgcgag
cgacgtttccgagtcacccccctctccgcccgctctcccagcagcatggctcctgctgcctctccacgtgtgctcgtcatcggagccggcgcgtcgcccgcgctcg
cgcgcgctcgactgacttggcaggcgtcgtgggcctaacgacggccgtggagatccagcgcgctctgcctgagggtgcgcagtcgctgcgctgcccgagctg
aggcctcagccgccgtgaccatcttcgcggcgcagacagccgaggatctcaagtcgacgcgctacaccagctcgtgggccggcgcgcatcacgtcacgctgg
agggccccggtctgcagcgcgactgcgaggtggacacgttcaaggcgctctgggcgctcgcgcacgacgatccgagcgtgccgttgctcgtgtgcccacaga
ccgaggtgctgtctgaccgccgcgcccgcttgctcgctccagcgatcggctgagcgagctgtacgcctcggcatgagccagctgacgccgatcaggtcttcgag
gatgcgtccaaggccgacttcatgcgcaagaatctcagctcgttcatgcccgacgtcaagccgctcgaaccttcagcgctgcccgaaggctgcacagcggggct
gtcgttcacgaccatcgcgctcgacacgccaaactatctgccatggctcgcgcagcgattccgcgcggccggcgggaccgtcgtccgtcgcgagctcggcaag
ctgagtgacgcgccgctcgacgagttcgacgccgtcgtcaactgctcgggcctaggcgcgctcacgctcgtcggcgacgacagcatgtacccgatccgcggcc
agaccgtgctcgtccgcgcgccgtggatccgttccggcatcacgcgatccggcagcgacaactggagtgcgttgccgtttcgttgtcgagctgatcgcgcagcat
acatcattccgcgcaagcagggcgacgtgattgttggcgggacacgcggcatcgacgactggtccgtcggcgcgcctggccagactgacgctgcaggcacga
gcagcctcgacccgaaacggcgtcggagatcctcgcgcgcggcctcaagctgtgccctgcgctgctgcccgaggcgaagcgggcgtcaatgcgcgtcgagg
acatcgacgtcgtggagcacggctggtccgttccgcttcgtcggccgcgctgacctcgcagcggcttccgaccggcgcgcaaaggcggcgtccgcatcgagct
cgaccacgtcgcggcgcccgacggtcggtcactgccgctcgtgcacaactacggccacgctggcgccggcttccagatgtcgatcggctcggcgactcgcgct
gtggatctgctGCGGTCTGCGCTCGCGTCCTGA
>Seq12 [organism=Ustilago maydis] D-amino acid oxidase gene, complete CDS
ttttacatattaaaattataactaactagatgagggtgagcaactgcgtgtggctcgataggaagatgcaagctccacacaccatccagaaacctgaccggttcgtta
tgaccataactacacgaatgccaatgagagcaatttgaacaagcgagcatgacgaccaagttctctctgaaccttgtgatttgttggctgttgtgacggaagcgagg
ataaccctattttcttggttacgatccgtgactgcatgcacagcgtcgcaatcgagcgtctgtgctctcgctttgggattcacgattttcgagaacgcactgtgaactca
ccactacacaattctgggtcacgatactcgtgactaaacatgcctggtttcatcattcgtgattcgtaatttgagggtcctgacacgcgagaaaagagggcttcaacc
atcggattccactaacagcttcgttcagatcatcaagggtttcctccgatcgcaaaagccaatctcccgatcgatttgagtttgctcagactgcctgttcctctctgctcc

gtaactctgcagattgcatgctaaactatcatctggcattgcctgaacggatcatgatctggcaactcgatagctcgctctcaaacgagcgcgcaaatgctgaccgc
ctgtcagctcagcgacgttcgagacacctttagccacgcatcgcttttggaattcgtctctgtccatcaaattaccttgacacctttgccgcacattctccgagatttcca
gtggtatcagccgagcatcttcgtctggctgacaaaagtatgctactgagaaacgagaaccactctcggactcgctgcggggcagcaccactcgatgctgtgtcg
aacggaacattccggcgctccaaaactccatcgcatcgcccacgttgaccacgatgcatccttttttgaggcggaatgtcaacccactggcccggctgcatttcgact
tgcagcccaccaacgtccttttgaaagagtaacgtgcaggagccgtagtccgagtgagaaccagcacggatgcttgtagttgccgatgtgtcagtagatgaatcgc
aaacggcagtcggaggatagtgaatcagccgcaatcgatcatgctggccatgatgtgaatcggcaaagtatctcgtaggcagatctatcgcctccgcaaagcctg
ctaaaactgtatcgcagacacccttgcatttttcgatgaaggtcgcaatcgcttctcgatgctgtgacagtgttggcggtagcgtctgtgaaggcggtcgatactgctc
ctcaactgtcgacgatccccccttcagctcgagagaggccagcgaggtagaacgactctttgagatcgccttcagccttgctctttggatcaagacgctcttgcatcat
cgatgtgtagcccgtattgttattccggtcctggtggctctggcgctcagcttccgactcatttaggaagaactcggcactttgactgaagagcagatcgatttcgtcct
ggttgataccatggtgggtgaggtagaagaagccggtcttgacgacggcatccaagatccgtgcaccgaggggtgctccagcgtcgtcgctatggagtgaaatg
ataggaatcggagttgcttcggccattctgatagcaaatgggttaaacttcagcggtccgctcggcctcgatctttggagaaaagtagaagagacagtcacgagta
acacagtgacagcgatcaagcaagaaacgttgagcaaacagaggtaaggtcccttggaactgcgctttgttttagtttttctgcctcaacaagcaactgtgcgccca
ctgaatttcccggtggaaaagccggcctgatacggcagttaccaagccaatgagatcaattcagaaaccacatatctccttggagccaacgtcgctcccgaccatc
gccacgcctactactcgcgtacctcgccacgctactacctctgatcagcattgccatctagcgcgatgacaaatcaacttcacgtagtggtcctcggcgctggagtg
cttggactgaccgatgcctttgagctgcgcgaacgcggctacagagtgaccatcctcgcacgcgatctacctttcgactctttctcccagaccttcgccagtccttgg
gctggagccaactggtgttccttcgctactctcgacgacaagccagcacagcgtcgagacgaaatcacgtttaagaaatggctcaagcttcacacgcgtttaccag
aggaagtaatggcaataatggagtttaccgatatcggcctcaccaagcgagataccaaggatgtatggttcagcaacctgactcctgaattcagcgtcctgccaga
cgccgatgaaatgggagcgcatgcaatcaagtacaaatcttttactattagcgtaccgctgtacacgcgctggttggtgtcggagttgacttcgaccaagcccattct
ccttgatgctacgcgagccggtccgccagtagaaatccgacgatgctccacgctaaccagcttatccgctgtacgatcactcgtaccaggctgcgatttggtagtca
atgcgacaggcgtaggagcggcagacttggccgacgtacgcgatcccaacgtctacccaattcgtggacaaaccgttcttatcaatgtaccatccttcgcctcccc
caatcgcgctgctcgctgcgttatgaagctctccaagcccaacgcctactacgtcattccacgagcaaggtctgggcaagtcatcctcggtggaagctttgaacttc
ggcaatcatccaccacccctgatcgcaacttggcggaaaggatcatggaagagtgcgcaaagctggtgcctgaaattgtacccgagggaaagacatggaaaga
tatcgacgttgtttctcacaatgtcggcttgagacctgctagggagaatggggctagggttgagttggagaggttgggaggaaatgggttgacggtggttcattcgt
atggaatcggtccggcaggctatcaggctagttttggtatcgcaaaggaggtggctgatctggtggataagcatgctggtcgccagtcgaggctgtag
>Seq13 [organism=Sporisorium reilianum] D-amino acid oxidase gene, complete CDS
cgtcgcaatcgagcatccgtccgcattttcgcaacgctcaagatcccggtcaccttttttcctctcttggaggtcggagtgtagaagagcagtatccaacaacagcc
gcagccatcgagagcacgtttgacagcttctgcatcgatttttcgggcgcttcctcggtcgctgtccgacgtcggctgtcatgagccggcttgtcgagcagcgggc
atcggctgcgtacagacggctgtcattgcgtccattctcgcttcaacgagtgcattgtgatggatgcgaatgatctgactgttgaggcgaggcgcgctcgtcatcatc
aggaacgaccttgcactcgaccaccacgcagcaagctgaacagacgaagctctgtggttggctcgagactgacgaagaccaaggagttccgtcgacgagctac
tgttttcgtgatattgttgtgcacgcgccatacagccttgcgacagcggatgtcctgttgtctaaaaaaagcacactcgtagcctcgaaacaaacaaagaccctgaat
gctcgcaaagcgtcttgtcgccgatgcatcgacgggctgtgtcccgatcgtctagtcgcttcggcgtcgaacttgtctctttaggcaacacccgcttgcggcagcgc
tggcgggatctctttcgtggatgcattgttcatgaggttcggcatcattcgatactacaagtagcttgcgttcagacgggcacgcagatgctgcccacccgtcaactc
ggcgacgtcggcgacgccctttgcaacgcatctcttgtgaaactcctctcggtccatcaagttgccttgatacttttgccgcaccgcatccgagatctccaacggaac
cagacgagcgtcttcgtccggctggcagaagtacgcgaccgagaagcgagagccgctctcactctcgctacgaggcagcaccacacgatgttgcgtcgagcg
gaacagtccagcgctccaaaactccatcgcatcgccgacgttgaccacaatgcatcccctttgcggtggaatgtcgacccactggcccggcttcatctcgacttgc
agcccgccgacgtccttttggaagagcagcgtacacgagccgtagtccgaatgagaacctgcacggatgcttgtcgtcgcagcgtcgtcgctcgatacgggagtt
ggaggatagtggatgaggcgcaatcgatcatgctgtccatggtgcgagtcggcaaagtaccgcgagggcagatcgatcgcttccgcaaagccggccaggatcg
tatcgcagacgcctttgcacttttcgatgaatgtcgcgagcacctctttgtgctgcgacagtgtgggcggcaacgactgtgacggaggtcgatagcgctcttctgcg
ggagacgagcctccttccaccctggccaggcccacgagatagaatgactccttgaggtcgccttccgccttgctccccgtatccagccgctccgacttcatcgccg
tatagcccgtgttgttggcgcggtcttggcattgtagccgctcgtgttccgcttcgtggaggaagaactgcgcgctctgatcgaacagcaggttgatctcatcctggc
ggatgccgtggtcggcgaggtagaagaagcccgtcttgacgacggcgtccaagatccgtgggcccaacgctgactcgtcgctgccgccggtgcggagggag
atgattggaatcggtgtcgagtccgacatctttgttgtgcgatgtgtcgaggaggacgagaaagcgggacagaagcagcgacgaacagacgagaggacccttg
acggagctttattttttgcttggctcgataagagagagagacggcccgctgattttcccggctgaaaagacggcgagaaacgattgtgcggacccaatcagaaga
agaaaagcgggactcgtacagctccgcaccaccttaccatcatccatttgtcgaccccattcctcgccgagctcgatacatcatacaacactgcaagaatgaccgg
acagtctcacgtcgtggtgcttggagccggaggtaagcaacgcttcagtagccagttgatgatgatcagcgcaactcactcgatttccgcttcctcgtgttgttcgac
agtgctcgggctgaccgacgcctttgagctgcgagagcgaggctacaaggtcaccatcctcgcccgcgatctgccgcaggactccttctctcaaacgttcgccag

tccttgggcggtgagtagcaaccacgacctcccaccagtcactcattgaagatcaccactgacacacctcaccttttcaccgcacgacacagggagccaactggt
gctcgttcgcaacgctcaccgacacaccagcccagcgtcgcgacgagatcaccttcaaaaagtggctccagctccacacccgattgccaccgcaagtcatggcc
atggtcgagttcaccgacatcagccccgtcaagcgcgaagccaaggacgtatggttcagccgactcactcccaacttcgccgtcatccccgaaggcgacggtac
gggcgcacacgcgatcaggtacgactcgttcaccatcagcgtgccgctgtacacgcagtggctggtctcggagctcacatccccgaagcccgtgctgttggacg
cggcgcgagcaggaccgccggtcgagatccgacgctgctcgacgctggcgtcgctggcggctgtgcggtcgctggtgccggggtgcgacgtggtcgtcaatg
cgacgggggtcggtgcgggcgatctggccgacgtgcgcgatcccgacgtgtatccgatccgcggacagacggtgctcgtgtccgtaccggcgttcaagagcc
ccaacaacggcgctcggtgcgtcatgaagctcggcagccccgccaagtacgtcatcccacgagcgcggtccggccaggtcatcctcggcggttcgtttgatgtt
cgacagtccagcaccacgcccgataaagcgctggcggacaagatcctgcaggactgcgccaagctcgtgccggagatcgtaccggagggcaagacgtggcg
cgagatcgatgtcatctcgcacaatgttggattgaggccaggcagggacaatggagccagggtggagctggagcacatccaacccaaggacggcgctgccag
gttgacggtggtccattcgtacggcatcggtccggcgggatatcaggccagctttggcatcgcgaaggaggtggctgacttggttgacggacacctcgctcgtca
agctcgattgtaa

(c) Protein sequences of Dao1

```
Feature for Sequence 'Seq1'
     gene            2001..3467
                     /gene='DAO1'
                     /note='D-amino acid oxidase gene'
     CDS             join(2001..2040,2149..2261,2326..2396,2456..2573,
                     2647..2848,2905..3467)
                     /gene='DAO1'
                     /EC_number='1.4.3.3'
                     /codon_start=1
                     /product='D-amino acid oxidase (Dao1)'
                     /translation='MHSQKRVVVLGSGVIGLSSALILARKGYSVHIVARDLPEDVSSQ
                     TFASPWAGANWTPFMSLTDGPRQAKWEELTFKKWVELVPTGQVMWLKGTRRFAQNEDG
                     LLGHWYKDITPNYRPLPSSECPPNSIGVTYDTLSVHAPKYCQYLARGLQKLGATFERR
                     TVTSVEQAFEGVDLVVNATGLGAKSIAGIDDQAAEPIRGQTVLVKSACKRCTMDSSDP
                     SSPAYIIPRPGGEVICGGTYGVGDWDLSVNPETVQRILKHCLRLDPSISSDGTIEGIE
                     VLRHNVGLRPARRGGPRVEAERLVLPLDRSKSPLSLGKGTTRAAKEKEVTLVHAYGFS
                     SAGYQQSWGAAEDVALLVEEAFQRYHGAARESKL'

Feature for Sequence 'Seq2'
     gene            2001..3467
                     /gene='DAO1'
                     /note='D-amino acid oxidase gene'
     CDS             join(2001..2040,2149..2261,2326..2396,2456..2573,
                     2647..2848,2905..3467)
                     /gene='DAO1'
                     /EC_number='1.4.3.3'
                     /codon_start=1
                     /product='D-amino acid oxidase (Dao1)'
                     /translation='MHSQKRVVVLGSGVIGLSSALILARKGYSVHIVARDLPEDVSSQ
                     TFASPWAGANWTPFMSLTDGPRQAKWEELTFKKWVELVPTGQVMWLKGTRRFAQNEDG
                     LLGHWYKDITPNYRPLPSSECPPNSIGVTYDTLSVHAPKYCQYLARGLQKLGATFERR
                     TVTSVEQAFEGVDLVVNATGLGAKSIAGIDDQAAEPIRGQTVLVKSACKRCTMDSSDP
                     SSPAYIIPRPGGEVICGGTYGVGDWDLSVNPETVQRILKHCLRLDPSISSDGTIEGIE
                     VLRHNVGLRPARRGGPRVEAERLVLPLDRSKSPLSLGKGTTRAAKEKEVTLVHAYGFS
                     SAGYQQSWGAAEDVALLVEEAFQRYHGAARESKL'

Feature for Sequence 'Seq3'
     gene            2001..3550
                     /gene='DAO1'
                     /note='D-amino acid oxidase gene'
     CDS             join(2001..2040,2120..2232,2312..2714,2791..2895,
```

```
                     2960..3027,3110..3550)
                     /gene='DAO1'
                     /EC_number='1.4.3.3'
                     /codon_start=1
                     /product='D-amino acid oxidase (Dao1)'
                     /translation='MAIDKRVVVLGTGVVGLSCGLVLSRQGYRVHFIARDLPEDSTSQ
                     GFASPWAGANWTPFYSRDEGPRQAKWEEATFARWVSLVPSGLAMWLNDTRRYADTDAG
                     LLGHWYRDTVRNYRELPPSELPKGVAAGAAYDTLSVNAPLYCQALARELQTLGATFER
                     RSVSSIEQVFEGQDDIALVVNATGLGAKSIAGIEDSACHPVRGQTVLVKSGCKRCTMD
                     SSNPEAPAYIIPRPGGEVICGGTYLVDDWDLSPSASTAQRILTQCLALDPSISTDGTL
                     DGIHILRHNVGLRPARTGGPRVEVGKLTLPLVRSTEPGTALALGTARPAPAGASSEAV
                     GAPSEAVKREVTLVHAYGFSSAGYQQSWGVAQDVLGLVEGEIGPPRAWWTQRGKL'

Feature for Sequence 'Seq4'
     gene            2001..3461
                     /gene='DAO1'
                     /note='D-amino acid oxidase gene'
     CDS             join(2001..2040,2150..2262,2327..2397,2454..2571,
                     2641..2842,2899..3461)
                     /gene='DAO1'
                     /EC_number='1.4.3.3'
                     /codon_start=1
                     /product='D-amino acid oxidase (Dao1)'
                     /translation='MHSQKRVVVLGSGVIGLSSALILARKGYSVHILARDLPEDVSSQ
                     TFASPWAGANWTPFMTLTDGPRQAKWEESTFKKWVELVPTGHAMWLKGTRRFAQNEDG
                     LLGHWYKDITPNYRPLPSSECPPGAIGVTYDTLSVHAPKYCQYLARELQKLGATFERR
                     TVTSLEQAFDGADLVVNATGLGAKSIAGIDDQAAEPIRGQTVLVKSPCKRCTMDSSDP
                     ASPAYIIPRPGGEVICGGTYGVGDWDLSVNPETVQRILKHCLRLDPTISSDGTIEGIE
                     VLRHNVGLRPARRGGPRVEAERIVLPLDRTKSPLSLGRGSARAAKEKEVTLVHAYGFS
                     SAGYQQSWGAAEDVAQLVDEAFQRYHGAARESKL'

Feature for Sequence 'Seq5'
     gene            2001..3461
                     /gene='DAO1'
                     /note='D-amino acid oxidase gene'
     CDS             join(2001..2040,2150..2262,2327..2397,2454..2571,
                     2641..2842,2899..3461)
                     /gene='DAO1'
                     /EC_number='1.4.3.3'
                     /codon_start=1
                     /product='D-amino acid oxidase (Dao1)'
                     /translation='MHSQKRVVVLGSGVIGLSSALILARKGYSVHILARDLPEDVSSQ
                     TFASPWAGANWTPFMTLTDGPRQAKWEESTFKKWVELVPTGHAMWLKGTRRFAQNEDG
                     LLGHWYKDITPNYRPLPSSECPPGAIGVTYDTLSVHAPKYCQYLARELQKLGATFERR
                     TVTSLEQAFDGADLVVNATGLGAKSIAGIDDQAAEPIRGQTVLVKSPCKRCTMDSSDP
                     ASPAYIIPRPGGEVICGGTYGVGDWDLSVNPETVQRILKHCLRLDPTISSDGTIEGIE
                     VLRHNVGLRPARRGGPRVEAERIVLPLDRTKSPLSLGRGSARAAKEKEVTLVHAYGFS
                     SAGYQQSWGAAEDVAQLVDEAFQRYHGAARESKL'

Feature for Sequence 'Seq6'
     gene            2001..3749
                     /gene='DAO1'
                     /note='D-amino acid oxidase gene'
     CDS             join(2001..2046,2174..2357,2440..2557,2682..2898,
                     2996..3100,3205..3272,3372..3749)
                     /gene='DAO1'
                     /EC_number='1.4.3.3'
```

```
                     /codon_start=1
                     /product='D-amino acid oxidase (Dao1)'
                     /translation='MPTREPQRVVVLGAGVIGLTCALSLVEQGYTVHVVARDLPEDTD
                     STAFASPWAGANVCPFKSLEEGPREARWETVTFKKLSTMIPSGLAMTLKGTRRFAHEE
                     SQLLGHWYKDVVPNYRHLNREECPPNAIGVEFDTISVNAPKYCHYLAHELRSKGVTIE
                     RRYVKSIEEVFRETFGARNDFVVNATGLGAKSIAGVEDQEVRPIRGQTVLIKSDCVRC
                     TMDSSDPTSSAYIIPRPGGEVICGGSYGVDDWDLSVSPDHAKRILSHCLRLDPSISRD
                     GTLEGIEILRHNVGLRPSRSSGPRVEKEIIELNHEKSPIRIGSRLDDATKGKSAKTRG
                     VVVHAYGVGPAGYQQSWGIAQDVVKLIQEGEARESKL'

Feature for Sequence 'Seq7'
     gene            2001..3629
                     /gene='DAO1'
                     /note='D-amino acid oxidase gene'
     CDS             join(2001..2210,2328..2453,2539..2610,2693..3363,
                     3457..3472,3567..3629)
                     /gene='DAO1'
                     /EC_number='1.4.3.3'
                     /codon_start=1
                     /product='D-amino acid oxidase (Dao1)'
                     /translation='MSAPPSQEPPCIVNVIGSGVLGLTCALELAKQPGSPYRVNVVTA
                     ESALTAWSQDPSTKLPVEPDFASPWAGAFWHPFTPQPETELQKRVAGWETKSFKHLWE
                     IAEQDPSVVMKADFKKYYDHRLTELPWYMDLCPEARKMNDEELAKAPEGKVDGIICKS
                     VSLNPIRYLDYLRKQLVKYEVQIIHHRLETVSEAFLGDPKFGIPAAKIVVNASGLGAG
                     SLCGVSDELMEPIRGQTILIRPPQPIQLITRDDDKCIYICSRPPTIPGEGEEVILGGS
                     YEPGNSSLSIDNGIADRILTEALKLRPDLSRDGTSNGIEILDHIAALRPSRKDGPRVE
                     RENFTISKKGETQDETGLLVHCYGIGGGGFQASYGAAAEVRSLIETQKNIS'

Feature for Sequence 'Seq8'
     gene            2001..3602
                     /gene='DAO1'
                     /note='D-amino acid oxidase gene'
     CDS             join(2001..2183,2295..2420,2506..2577,2656..3332,
                     3424..3439,3546..3602)
                     /gene='DAO1'
                     /EC_number='1.4.3.3'
                     /codon_start=1
                     /product='D-amino acid oxidase (Dao1)'
                     /translation='MSNVNVIGSGVLGLTCALELAKQPGSPYRVTVVTAENAMARWSQ
                     DPSTKLPVEPDFASPWAGAFFHPFTPQPETELQKRVASWERKSFKPLWEIAEQDPSVV
                     MKTDFKKYYNHPLSELPWYMDLFPEARKMNAEEIANGPEGKVDGLICKSVTLNPIRYL
                     DYLRQQLAKYDVRIIHHRLETVTEAFEGHPECGLPAAKIVINACGLGAGKIGGVSDGM
                     MEPIRGQTMLIRPPQPLQLITRDDEKCIYICSRPPTIPGEGEEVVLGGSYQPGDSSLE
                     IDDQISHRILSEALKIRPDLSHDGTVEGVQIVEHVAALRPHNKNGPRVEREDFHIKST
                     SQGETQNSTGILVHCYGIGGGGFQASYGVAEEVRNLVEQGRS'

Feature for Sequence 'Seq9'
     gene            2001..3585
                     /gene='DAO1'
                     /note='D-amino acid oxidase gene'
     CDS             join(2001..2207,2283..2597,2770..3296,3397..3412,
                     3514..3585)
                     /gene='DAO1'
                     /EC_number='1.4.3.3'
                     /codon_start=1
                     /product='D-amino acid oxidase (Dao1)'
                     /translation='MSFNPEHKSEHLNIIGSGVLGLTCALEIAKEKGRYTITIITADA
```

```
                    DSSTWSPSAPPKIEKVAEDFASPWAGAYWQSFVATRDPKTFNEKRLQDWEKTSFKELW
                    KISETDKSIVMVKYLILGVLSPRDVFVETSQHYSSKPIVTNILMKNLVTMSYLGTPIC
                    VQRLAKVFAIQLCQMGVRIVHHRLNSLAEAFEGNNTLQIPRADIVINASGLGAATLLG
                    VEDKSVHPIRGQLVLVKPPQPICFSTRDSSRKTYIISRPSVDPEIDEEVILGGCYQAD
                    NFDLSVDPDLTNHILCEAFQTRPDLSSDGTLQGIHVLKEVVALRPARKDGARLEVEKV
                    VISGENKHAVHCYGIGGAGFQSSYGMAQEALGLIKALREGRETV'

Feature for Sequence 'Seq10'
     gene           2001..3866
                    /gene='DAO1'
                    /note='D-amino acid oxidase gene'
     CDS            join(2001..2177,2249..2273,2383..2468,2586..2654,
                    2801..3222,3293..3420,3487..3657,3760..3866)
                    /gene='DAO1'
                    /EC_number='1.4.3.3'
                    /codon_start=1
                    /product='D-amino acid oxidase (Dao1)'
                    /translation='MTLPLLEDAPRALDAVIIGAGVTGLSIGLELVKRGYKIAVVAKD
                    LPEDDQSPGFASPWAGCNWCSFADNNKREQEWDRKTYQALFNVAENHPNLCELIQFHS
                    YESDKLPEPWFKDLVKDYKVTKEGTKPGERCISYKSFIMHAPNYLRHLAQQLRSAGVP
                    IQRKRLTSLEEAYNLPSYGRVNLVINATGLGARSLLGNKDPSDIYPIRGQTVLVKVPQ
                    GWKKTCFMMASDHPDEKTTKDTEIPEPTYIIPRPGPEGHVVLGGSFQVNNWEYAPDYA
                    LAERILKRNYELCPELAGPNGKSWKDIEIVAHNVGFRPSRQGGCRLDLEPIELGNAKQ
                    EGMQLAPKSTITEPRTGAILHAYGIGPAGFQASLGYALEAGDMVDSYFKKGDAKKRSS
                    KL'

Feature for Sequence 'Seq11'
     gene           2001..3328
                    /gene='DAO1'
                    /note='D-amino acid oxidase gene'
     CDS            join(2001..2043,2085..2135,2173..2372,2463..2847,
                    2884..2947,2983..3114,3151..3328)
                    /gene='DAO1'
                    /EC_number='1.4.3.3'
                    /codon_start=1
                    /product='D-amino acid oxidase (Dao1)'
                    /translation='MAPAASPRVLVIGAGVVGLTTAVEIQRALPEAAVTIFAAQTAED
                    LKSTRYTSSWAGAHHVTLEGPGLQRDCEVDTFKALWALAHDDPSVPLLVCPQTEVFED
                    ASKADFMRKNLSSFMPDVKPLEPSALPEGCTAGLSFTTIALDTPNYLPWLAQRFRAAG
                    GTVVRRELGKLSDAPLDEFDAVVNCSGLGALTLVGDDSMYPIRGQTVLVRAPWIRSGI
                    TRSGSDNWTYIIPRKQGDVIVGGTRGIDDWHEQPRPETASEILARGLKLCPALLPEAK
                    RASMRVEDIDVVEHGCGFRPARKGGVRIELDHVAAPDGRSLPLVHNYGHAGAGFQMSI
                    GSATRAVDLLRSALAS'

Feature for Sequence 'Seq12'
     gene           2001..3098
                    /gene='DAO1'
                    /note='D-amino acid oxidase gene'
     CDS            2001..3098
                    /gene='DAO1'
                    /EC_number='1.4.3.3'
                    /codon_start=1
                    /product='D-amino acid oxidase (Dao1)'
                    /translation='MTNQLHVVVLGAGVLGLTDAFELRERGYRVTILARDLPFDSFSQ
                    TFASPWAGANWCSFATLDDKPAQRRDEITFKKWLKLHTRLPEEVMAIMEFTDIGLTKR
                    DTKDVWFSNLTPEFSVLPDADEMGAHAIKYKSFTISVPLYTRWLVSELTSTKPILLDA
                    TRAGPPVEIRRCSTLTSLSAVRSLVPGCDLVVNATGVGAADLADVRDPNVYPIRGQTV
```

```
                    LINVPSFASPNRAARCVMKLSKPNAYYVIPRARSGQVILGGSFELRQSSTTPDRNLAE
                    RIMEECAKLVPEIVPEGKTWKDIDVVSHNVGLRPARENGARVELERLGGNGLTVVHSY
                    GIGPAGYQASFGIAKEVADLVDKHAGRQSRL'

Feature for Sequence 'Seq13'
     gene           2001..3267
                    /gene='DAO1'
                    /note='D-amino acid oxidase gene'
     CDS            join(2001..2040,2119..2231,2314..3267)
                    /gene='DAO1'
                    /EC_number='1.4.3.3'
                    /codon_start=1
                    /product='D-amino acid oxidase (Dao1)'
                    /translation='MTGQSHVVVLGAGVLGLTDAFELRERGYKVTILARDLPQDSFSQ
                    TFASPWAGANWCSFATLTDTPAQRRDEITFKKWLQLHTRLPPQVMAMVEFTDISPVKR
                    EAKDVWFSRLTPNFAVIPEGDGTGAHAIRYDSFTISVPLYTQWLVSELTSPKPVLLDA
                    ARAGPPVEIRRCSTLASLAAVRSLVPGCDVVVNATGVGAGDLADVRDPDVYPIRGQTV
                    LVSVPAFKSPNNGARCVMKLGSPAKYVIPRARSGQVILGGSFDVRQSSTTPDKALADK
                    ILQDCAKLVPEIVPEGKTWREIDVISHNVGLRPGRDNGARVELEHIQPKDGAARLTVV
                    HSYGIGPAGYQASFGIAKEVADLVDGHLARQARL'
```

Sequence S2. *ACT1* gene sequences (Submitted GenBank sequence record ahead of release)

```
LOCUS       Seq14                  2400 bp    DNA     linear       15-APR-2015
DEFINITION  Actin gene (ACT1), complete CDS.
ACCESSION   Seq14
VERSION
KEYWORDS    .
SOURCE      Rhodosporidium toruloides ATCC 10657
  ORGANISM  Rhodosporidium toruloides ATCC 10657
            Unclassified.
REFERENCE   1  (bases 1 to 2400)
  AUTHORS   Liu,Y., Koh,C.M.J. and Ji,L.
  TITLE     Engineering an efficient and tight D-amino acid-inducible gene
            expression system in Rhodosporidium/Rhodotorula species based on a
            D-amino acid oxidase gene promoter
  JOURNAL   Unpublished
REFERENCE   2  (bases 1 to 2400)
  AUTHORS   Liu,Y., Koh,C.M.J. and Ji,L.
  TITLE     Direct Submission
  JOURNAL   Submitted (15-APR-2015) Biomaterials and Biocatalysts Group,
            Temasek Life Sciences Laboratory, 1 Research Link, National
            University of Singapore, Singapore, Singapore 117604, Singapore
COMMENT     Bankit Comment: TAX: No, not new species/combinations; SEE
            ATTACHMENT.
            Bankit Comment: ALT EMAIL:hydrolase@gmail.com.
            Bankit Comment: TOTAL # OF SEQS:1.

            ##Assembly-Data-START##
            Sequencing Technology :: Sanger dideoxy sequencing
            ##Assembly-Data-END##
FEATURES             Location/Qualifiers
     source         1..2400
                    /organism='Rhodosporidium toruloides ATCC 10657'
                    /mol_type='genomic DNA'
     gene           <1..>2400
```

```
                     /gene='Actin gene (ACT1)'
     mRNA            join(<1..614,748..856,915..1022,1084..1656,1716..>2400)
                     /gene='Actin gene (ACT1)'
                     /product='Actin'
     CDS             join(118..614,748..856,915..1022,1084..1656,1716..2357)
                     /gene='Actin gene (ACT1)'
                     /codon_start=1
                     /product='Actin'
                     /translation='MSSPPRFAPGSTSPTQSRPRATHSSPTQGPFATPARPGASSAAH
                     QPDPSTSTAGAASLLTSSPHYTTSLRSRHSLYGTEDRVVLDLGSRIWKVGFSGEPQPR
                     ECRSVVSELAHERAGRRAGPSIGARGDDDEEDCFWALEKAEPSEEEWLIREERVKRLL
                     RKIWFENLMIDPKTRKVIVVENPLLSTRVKEMIARVLFDNLQIPSLSFASAPLLALMA
                     AGTVTGLVVDVGNLETTVLPVFHARPLFPSLTTTPRAGSRLNRRLRSLLLAFGSYAPP
                     PSSLNSMTPPAIGRIPKELLTEELIEEIKTRLCFVGEEVPLDASRGEREASAFSGSAM
                     SVDTASARDNFDDPSDPDNALLKELYSRFAATSTAKPVSFRIPNLSQPAIANGTGRGW
                     IQVPGWIRERAAEVLWEEDGDGDERGLAAVVLDCLLKLPLDLRKPMASSILLTGGTAM
                     LPGFFPRFKAALLAQLDRSHPPSPPPSPPLPAASVEPPSSDPAGPMSTDTAASPAPSR
                     SSEVNAKRRRKHALATRLHNLRHSPRYAPLVPLARHLAILNHPSPNSSASSTAPSSTL
                     ARQREGSAPSFSPALQSWIGGSLAGALKTGGPEIAREQWDAGLRFAEAEEAEGEEGEE
                     FEEREVIRPALPDWTRIA'
BASE COUNT      413 a    828 c    696 g    463 t
ORIGIN
        1 tcctctggcc tctctcgctc cgttcgcctg gccgcgcgcc caaaccgctt tgttgacgct
       61 atcagcctcc cgcgagcgct agcaggctct cttagccgcc tcacgcctac ctcgcgaatg
      121 tcctccccac ctcgcttcgc gcccggttcg acctcgccca cgcagtcgag gccgcgtgcg
      181 acgcacagca gcccaacaca aggtcccttc gcaacgcctg cgcgcccagg tgcgagctca
      241 gcggcgcacc aacccgatcc ttcaacctcg acagcaggcg ccgcctccct cctcacgtct
      301 tctcctcact acacgacttc gctgcgctct cgacactcgc tttacggcac ggaagaccgt
      361 gttgtgctgg atctcggctc gcggatatgg aaggtcgggt tcagcgggga gccgcagccg
      421 cgcgagtgcc ggagcgtcgt gagcgagttg gcgcatgagc gggctgggcg aagagcaggg
      481 ccgtccatag gcgcgagagg ggacgacgac gaagaggact gcttttgggc gctcgagaag
      541 gccgagccga gcgaggagga gtggttgatt cgcgaggaga gggtgaagcg attactgcgc
      601 aagatctggt tcgagtgagt tcaaccggtc tttcttttga taccaggact tgcgctcgcc
      661 aacctgggcg gcgctttctg ctaagaggct cgagtacgaa ccggacaagc ggagtctttt
      721 ccgccggctg acttcgttct cccgcagaaa cctcatgatc gacccgaaga cgcgcaaagt
      781 catcgtcgtg gagaacccac tgctgtcgac gcgcgtgaag gagatgatcg cgcgggtctt
      841 gttcgacaac ttgcaggtaa gccatccgcc gcttcgccag gccgttcacc cgtactgacc
      901 gcttcgctac gcagatcccg tcgctcagct tcgcttccgc ccccttgctc gccttgatgg
      961 cggccggcac agtgaccggt cttgtggtcg acgtcggaaa cctcgagacg accgttcttc
     1021 ccgtgagttc gccttccgct ctcgcctgct tactaaactt gcgctgatgt agagctacaa
     1081 caggtctttc acgctcgccc gctctttccc tccctcacca caactcctcg cgcgggctct
     1141 cgcctgaacc gccgcctccg ctctctcctc ctcgcattcg gctcatacgc acctcctccc
     1201 tcctccctca actccatgac gcctcccgct atcggacgga taccgaagga gctcttgacg
     1261 gaggagctca tagaggagat caagacgcgg ctgtgttttg tcggtgagga ggtcccgctc
     1321 gatgcgagcc ggggcgagag ggaggcgtcc gcgttcagcg gatcggccat gagcgtcgac
     1381 actgcatcag cacgcgacaa ctttgacgac ccttccgacc ccgacaacgc actactaaag
     1441 gagctttact cccgcttcgc cgcgacatcg accgccaaac ccgtttcctt ccggataccc
     1501 aacttgtccc aacctgcgat cgcgaacggt acaggtcgag ggtggatcca ggtgcccggc
     1561 tggatcaggg agcgcgccgc cgaggtgttg tgggaggagg atggtgatgg agacgagcgt
     1621 gggcttgccg ccgttgtcct tgactgccta ttgaaggtgc gcaagctgac cacttcgctc
     1681 gagcgaagta gctgacactg cacccgttcc cccagctccc ccttgacctt cgaaagccga
     1741 tggcctcgtc aatcctcctc acgggcggca ccgccatgct ccccggcttc ttcccacgct
     1801 tcaaggccgc tcttcttgcc cagctcgacc gctcgcatcc tccttcccct ccgccttccc
     1861 cgcctctgcc agcagcatcc gtcgaacctc cttcttccga ccctgccgga cccatgtcga
     1921 cggacacggc ggcatcgcct gcgccctctc gctcgagcga agtcaatgcg aagcggcggc
     1981 gaaagcatgc cctcgcgact cgactgcaca acctgcggca ttcgcctcga tacgccccgc
     2041 ttgtccctct cgctcgacac ctcgccatct tgaaccaccc atctccgaac tcctcggcat
```

```
2101 cgtcgacggc gccctcatca accctcgctc gacagcgcga aggttcggct ccgtctttct
2161 cgcccgcctt gcaaagctgg atcggcggca gcctcgcagg agcgctcaag acgggcgggc
2221 ctgagattgc gagggagcag tgggatgcag ggttgcggtt tgcggaggca gaagaggcgg
2281 agggagagga aggagaggag tttgaggagc gggaagtgat ccggccggct ctgccggact
2341 ggacgaggat cgcgtaggat gatggtgtat cacgcgtgtt gccaagacag ttttacaggc
//
```
