# Supplementary material for: Engineering an efficient and tight d-amino acid-inducible gene expression system in Rhodosporidium/Rhodotorula species
Source: Microb Cell Fact. 2015 Oct 26;14:170. doi: 10.1186/s12934-015-0357-7 (PMC4624585; doi:10.1186/s12934-015-0357-7)
Supplement: Supplementary file 6 — 10.1186/s12934-015-0357-7 Sequence of the mutation promoter PDAO1m5-in1m1 (renamed as PDAO1int). [file 12934_2015_357_MOESM6_ESM.pdf]

Additional file 6. Sequence of the mutation promoter $P_{DAO1m5\text{-}in1m1}$ (renamed as $P_{DAO1int}$)

>$P_{DAO1m5\text{-}in1m1}$ (-509~+159, mutation was labeled in red font)

cgttcgtgggctcaaggaagtcctcccccgtcggctgcgcatcacacggagcatacgcaacatggaacaagcctcctacgcgtccttga
cgcaacttcgggaggtcgacatggcctgcaagaccggtcgcaagttcagggaggacgtcgagcggtctccggcagaggtggcgagcgac
gtaggggaggtcgacgtgtccgtctatgagtgggtggcgtttcaggatcccgcgggcctcagcgagcaaggactcgttgggcaaagtga
aggtgtactgatgagcggttgcgaggccgcagagcagcgctgcgacgacgggaagcttcggcacgagcatgactgtgagtagtagtcca
aggagaacagcgcagagtcggcaggagggcacatggaggcagagcgtggggcggaggaggcagatggggagtcgcgctgggggacgaga
gggtgccgctcgaccaactgctctctttcgctcttgctgctgcttgtactgctcgaacgacgccat**c**cattcacagaagcgcgtcgttg
tcctcggatcaggcggtgcgtctttccctctcctccccacacccgacagttctcgaggaggagtacagcagcgagcgaggctgccgagg
gggatctgggttgacgcagctcttgattatacagtgattg**ccatg**
